# Paediatric differentiated thyroid carcinoma: a UK National Clinical Practice Consensus Guideline

**DOI:** 10.1530/ERC-22-0035

**Published:** 2022-06-23

**Authors:** Sasha R Howard, Sarah Freeston, Barney Harrison, Louise Izatt, Sonali Natu, Kate Newbold, Sabine Pomplun, Helen A Spoudeas, Sophie Wilne, Tom R Kurzawinski, Mark N Gaze

**Affiliations:** 1 Centre for Endocrinology, William Harvey Research Institute, Barts and the London School of Medicine and Dentistry, Queen Mary University of London, Charterhouse Square, London, UK; 2 Department of Paediatric Endocrinology, Barts Health NHS Trust, London, UK; 3 Whipps Cross Hospital, Barts Health NHS Trust, London, UK; 4 Retired Endocrine Surgeon, Sheffield, UK; 5 Department of Clinical and Cancer Genetics, Guy’s and St Thomas’ NHS Foundation Trust, London, UK; 6 Department of Pathology, University Hospital of North Tees and Hartlepool NHS Foundation Trust, Stockton-on-Tees, UK; 7 Department of Clinical Oncology, Royal Marsden Hospital Foundation Trust, London, UK; 8 Department of Pathology, University College London Hospital NHS Foundation Trust, London, UK; 9 Department of Paediatric Endocrinology, Great Ormond Street Hospital for Children NHS Foundation Trust, London, UK; 10 Department of Paediatric Oncology, Nottingham University Hospital’s NHS Trust, Nottingham, UK; 11 Department of Endocrine Surgery, University College London Hospitals NHS Foundation Trust, London, UK; 12 Department of Paediatric Endocrine Surgery, Great Ormond Street Hospital for Children NHS Foundation Trust, London, UK; 13 Department of Clinical Oncology, University College London Hospitals NHS Foundation Trust, London, UK; 14 Department of Clinical Oncology, Great Ormond Street Hospital for Children NHS Foundation Trust, London, UK

**Keywords:** thyroid, papillary thyroid cancer, follicular thyroid cancer, paediatric cancer, differentiated thyroid cancer

## Abstract

This guideline is written as a reference document for clinicians presented with the challenge of managing paediatric patients with differentiated thyroid carcinoma up to the age of 19 years. Care of paediatric patients with differentiated thyroid carcinoma differs in key aspects from that of adults, and there have been several recent developments in the care pathways for this condition; this guideline has sought to identify and attend to these areas. It addresses the presentation, clinical assessment, diagnosis, management (both surgical and medical), genetic counselling, follow-up and prognosis of affected patients. The guideline development group formed of a multi-disciplinary panel of sub-speciality experts carried out a systematic primary literature review and Delphi Consensus exercise. The guideline was developed in accordance with The Appraisal of Guidelines Research and Evaluation Instrument II criteria, with input from stakeholders including charities and patient groups. Based on scientific evidence and expert opinion, 58 recommendations have been collected to produce a clear, pragmatic set of management guidelines. It is intended as an evidence base for future optimal management and to improve the quality of clinical care of paediatric patients with differentiated thyroid carcinoma.

## Introduction

Differentiated thyroid cancer (DTC), the most common endocrine cancer in children, occurs in only a small number of patients in the United Kingdom. Approximately, 13 cases per year are seen in children aged less than 15 years and about 105 cases per year in those aged 15–24 years (children, teenagers and young adults UK cancer statistics report 2021: http://www.ncin.org.uk/cancer_type_and_topic_specific_work/cancer_type_specific_work/cancer_in_children_teenagers_and_young_adults/). It increases in frequency with age: of the total cases seen in those aged 0–24 years, 1% occur in those aged 0–4 years, 2% in those 5–9 years, 8% in those 10–14 years, 28% in teenagers 15–19 years and 61% in young adults 20–24 years. There is a female predominance which increases with age: 69% of cases aged 0–14 years and 80% of cases aged 15–24 years are female (children, teenagers and young adults UK cancer statistics report 2021: http://www.ncin.org.uk/cancer_type_and_topic_specific_work/cancer_type_specific_work/cancer_in_children_teenagers_and_young_adults/). The sex ratio is nearly equivalent in children under 10 years of age (Harach & Williams 1995). The overall incidence of thyroid cancer in the 0–24 age group is 1.2 per 100,000 in females and 0.5 per 100,000 in males (National Registry of Childhood Tumours 1991–2010). The incidence appears to be increasing (Vergamini *et al.* 2014, Golpanian *et al.* 2016) and this is largely thought to be due to better detection of asymptomatic disease through the increasing use of medical imaging. Less than 1% of thyroid cancers are linked to a risk factor such as previous thyroid irradiation (Lazar *et al.* 2009). Familial non-medullary thyroid cancer (NMTC) (is described in 3–9% of cases presenting at any age (Moses *et al.* 2011, Peiling Yang and Ngeow 2016). Genetic predisposition syndromes account for around 5% of NMTCs (Vriens *et al.* 2009, Richards 2010, Peiling Yang and Ngeow 2016); 95% is accounted for by non-syndromic forms (Peiling Yang & Ngeow 2016, Kamani *et al.* 2022); 80–90% of DTCs in children are papillary carcinoma and 5–20% follicular carcinoma (Hogan *et al.* 2009, Papendieck *et al.* 2011, Mussa *et al.* 2013, De Jong *et al.* 2021).

In all age groups in the United Kingdom, overall DTC mortality has decreased by 46% since the early 1970s (Lee *et al.* 2019). Although the risk of aggressive and recurrent disease in children and young people (CYP) with DTC is higher than in adults (Alessandri *et al.* 2000, Jarzab *et al.* 2000, Hay *et al.* 2010, Al-Qahtani *et al.* 2015, Lee *et al.* 2015), the 10-year cause-specific survival is better (Brink *et al.* 2000, Chow *et al.* 2004, Steliarova-Foucher *et al.* 2006, Huang *et al.* 2012, Shayota *et al.* 2013). No deaths were reported in children, teenagers and young adults with DTC from 2012 to 2014, and the age-standardised mortality rate for all thyroid cancer per 100,000 population is 0 for the 0–24 age group (data from the National Registry of Childhood Tumours). However, deaths from DTC in childhood may still occur after this age range.

In order to promote best practice standards for the diagnosis and management of thyroid cancers, the American Thyroid Association (ATA) (Haugen *et al.* 2016), the American Association of Clinical Endocrinologists (Gharib *et al.* 2016), the National Comprehensive Cancer Network (NCCN) (NCCN 2016) and the British Thyroid Association (BTA)/Royal College of Physicians (Perros *et al.* 2014) have published guidelines specifically addressing the evaluation, treatment and follow-up of thyroid nodules and DTC in adults.

In most cases, the evaluation, treatment and follow-up of children with thyroid cancer have followed adult guidelines. This approach results in excellent short- and intermediate-term outcomes but may have resulted in overtreatment for a disease with excellent prognosis in CYP, with significant late effects including second malignancy. More recently, specific paediatric guidelines have been provided by the ATA (Francis *et al.* 2015) and from the Netherlands (Lebbink *et al.* 2020), but many areas of the treatment of CYP with DTC remain controversial. With recent progress in the management of CYP with this condition, there is an ongoing need for up-to-date age-appropriate guidelines for the management of DTC in CYP that acknowledges the specific needs of this patient group.

## Methods

**Figure 1:**
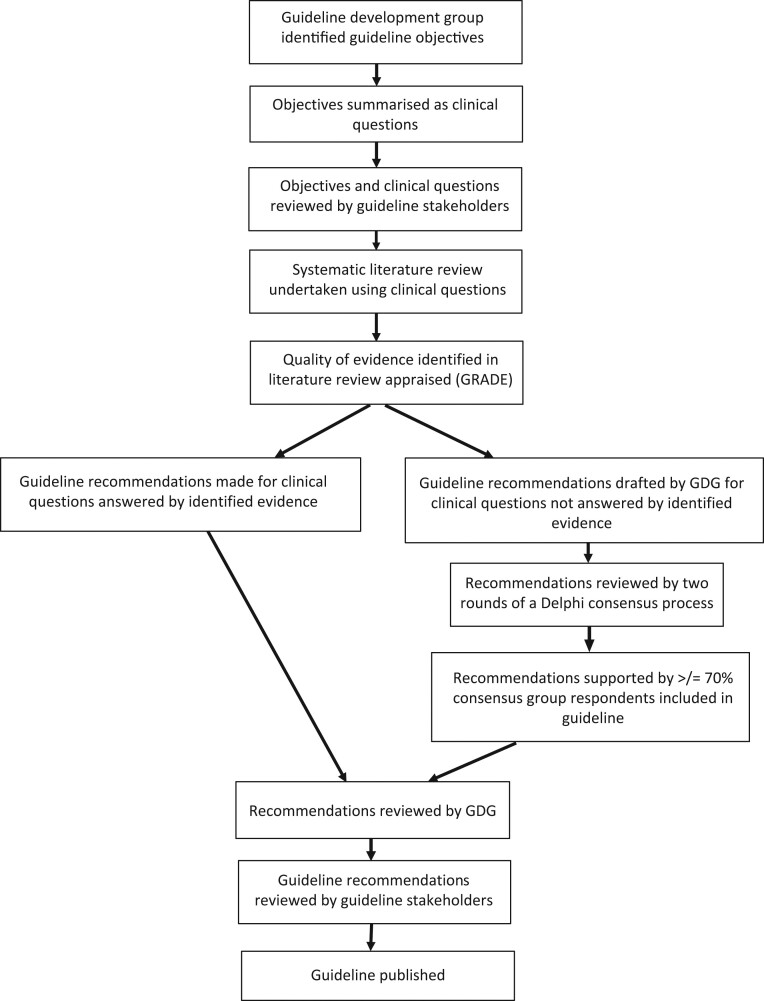
Guideline development process. GDG, Guideline Development Group; GRADE, Grading of Recommendations, Assessment, Development and Evaluations (Guyatt *et al.* 2011).

A multidisciplinary Guideline Development Group (GDG) oversaw guideline development. The GDG members, as well as stakeholders and Delphi Consensus panel, are listed in Supplementary Appendix 1 (see section on supplementary materials given at the end of this article). This guideline was developed in accordance with The Appraisal of Guidelines Research and Evaluation Instrument II criteria, as specified in the Children’s Cancer and Leukaemia (CCLG) guideline development standard operating procedure, version 6 (https://www.cclg.org.uk/write/MediaUploads/What%20we%20do%20section/CCLG_guideline_SOP_v_6.pdf). The methodology is summarised in Fig. 1. Different stages of the guideline development process were overseen and appraised by the Quality Improvement Committee of the Royal College of Paediatrics and Child Health (RCPCH).

## Conflicts of interest

All GDG and Delphi Consensus group participants were asked to declare any conflicts of interest as per the National Institute of Health and Care Excellence (NICE 2018) conflicts of interest policy. Conflicts were reviewed and no relevant conflicts were identified. The CCLG provided administrative support throughout the guideline, and the RCPCH provided advice and appraised the guideline at different stages.

## Developing the clinical questions

The GDG devised the scope and, subsequently, the Population, Intervention, Comparison, Outcome (PICO) questions (Akobeng 2005), which were sent out to stakeholders to ensure no relevant area had been omitted. Feedback from stakeholders was taken into account by the GDG in the finalisation of the PICO questions, which were used to direct a systematic literature search (Supplementary Appendix 2). *Stakeholder involvement* *:* Views from the target population (DTC patients, survivors and their families) were sought via the stakeholder consultation process through various patient support groups including the Royal College of Physicians Young Adult and Adolescent Steering Group. Stakeholders were given the opportunity to comment on the PICO questions being asked and on the final guideline recommendations made to facilitate brevity, clarity and fairness. Recommendation 22 and the section on health benefits (Recommendation 38) are specific to the needs of children and their parents.

## Identifying the evidence

Literature searches were conducted as detailed in Supplementary Appendix 3. Of 2565 papers found using this search strategy, 238 papers met the inclusion criteria and were included in the guideline evidence base. The search strategy was limited by prior agreement of the overarching Project Board for all eight National Rare Paediatric Endocrine Tumour Guidelines to publications pertaining to CYP with DTC-related pathology before 19 years of age, including fully published case reports and case series. Full inclusion/exclusion criteria can be found in Supplementary Appendix 3. An additional 26 papers were included following the peer-review process prior to publication.

## Reviewing and synthesising the evidence

The quality of evidence identified in the systematic search was appraised using the Grading of Recommendations, Assessment, Development and Evaluations criteria (Guyatt *et al.* 2011). Details of this process can be found in Supplementary Appendix 4.

## Developing recommendations

Where the literature search identified evidence to answer the PICO questions, the GDG made a guideline recommendation. The strength of the recommendation was determined by the trade-off between the potential benefits and potential harms of the recommendation, taking into account the quality of the underpinning evidence. Where an evidence base to formulate recommendations was lacking (i.e., no evidence, contradictory evidence or very low-quality evidence), an expert consensus was necessary. These recommendations were evaluated using a formal Delphi Consensus process (Supplementary Appendix 5) (Okoli & Pawlowski 2004). A recommendation was deemed to have achieved consensus if 70% or more of the Delphi respondents (excluding those who indicated inadequate expertise in the posed question area to be able to comment) supported the recommendation as framed, or with minor modification.

The evidence supporting each recommendation is summarised following the recommendation. In situations where no or low-quality evidence was available, but a Delphi Consensus was not achieved and there was no possibility of near-future comparison trials, the recommendation was made by the GDG Consensus. If there was additionally a clear widespread clinical best practice, that was also used to support strengthening the recommendation. We followed a consistent NICE terminology, using the verbs ‘offer’ and ‘consider’ for strong and less strong interventions/actions, respectively and the verbs ‘should’ for strong and ‘may’ and ‘consider’ for moderate recommendations. All recommendations were reviewed by the Project Board and four selected peer experts, prior to guideline publication. Areas highlighted by the literature review and consensus process in which the GDG felt further research would be valuable are reported under the heading, Research Recommendations (Supplementary Appendix 6).

## Recommendations

The GDG made 40 recommendations based on the identified evidence. Thirty further recommendations were made based on GDG expert opinion, and these were reviewed by two rounds of a Delphi Consensus process (Supplementary Appendix 5). Following this, 18 recommendations achieved consensus and were included in the guideline. Each recommendation is directly followed by a section discussing the related evidence and citations for this recommendation. One recommendation was made on the basis of GDG Consensus only. Areas highlighted by the literature review and consensus process where the GDG felt further research would be valuable have been proposed as research recommendations (Supplementary Appendix 6).

Health benefits, side effects and risks have been considered for all recommendations on the diagnosis, management and follow-up. The relevant factors are discussed for each recommendation in the general text. Examples include recommendations to assess vocal cord function pre-operatively; diagnosing and managing post-operative complications; safety precautions when using radioiodine, including fertility preservation; and how to follow-up these patients safely to balance over-investigation with timely diagnosis of recurrent disease. The guideline also highlights the need for surgery to take place at high-volume tertiary centres.

## External review

The guideline was then externally peer-reviewed by four independent reviewers to improve the quality and applicability of the guideline (see Supplementary Appendix 1). The RCPCH, via the Quality Improvement Committee Clinical Leads for Evidence Medicine and Appraisals, provided advice on guideline development and appraised the draft for quality at different stages. Feedback from the endorsing body and experts was used to complete the final document.

## Results

### Differentiated thyroid cancer: presentation

1. **Refer CYP with a diffusely or focally enlarged thyroid to an age-appropriate centre with expertise in the management of thyroid disease that can undertake all necessary investigations and treatment** *(Strong Recommendation, Delphi Consensus 93%)*
2. **Care for CYP with suspected or proven DTC in an age-appropriate tertiary centre linked to a paediatric or teenage and young adult oncology centre. A designated specialist clinician who has expertise in the investigation and treatment of patients with thyroid cancer should coordinate multidisciplinary care (endocrinology, surgery and oncology)** *(Strong Recommendation, Delphi Consensus 100%)*
  Recommendations 1 and 2 are based on national policy endorsed by NICE Improving Outcomes in Children and Young People with Cancer Guidelines, 2005 (NICE 2018).
3. **Investigate CYP with the following presentations for DTC:**
  - **a solitary thyroid nodule (whether symptomatic or incidentally identified on imaging of the neck)**
  - **an enlarged nodular thyroid** *(Strong Recommendation, Very Low-Quality Evidence, Delphi Consensus 100%)*
  Less common presentations of thyroid cancer include cervical lymphadenopathy, dysphonia, dyspnoea and stridor, as reported in several retrospective cohort studies (Papendieck *et al.* 2011, Lazar *et al.* 2009). There is no reported association between nodule size and malignancy risk (Corrias & Mussa 2013, Chiu *et al.* 2012).
4. **Clinicians should have a higher index of suspicion for DTC in CYP with a history of prior head and neck irradiation or family history of DTC (see Recommendation 10)** *(Strong Recommendation, Very Low-Quality Evidence, Delphi Consensus 100%)*
  Retrospective cohort studies of CYP previously treated for cancer with head and neck radiotherapy, especially those where the thyroid gland is included in the treatment field, have been shown to be at increased risk of developing DTC (Sigurdson *et al.* 2005, Brignardello *et al.* 2008, Lazar *et al.* 2009, Taylor *et al.* 2009, Bhatti *et al.* 2010, Papendieck *et al.* 2011, Veiga *et al.* 2012, Kiratli *et al.* 2013, Goldfarb & Freyer 2014, Klein Hesselink *et al.* 2016). Those at risk include patients treated for Hodgkin lymphoma, leukaemia, CNS tumours (Soberman *et al.* 1991, Solt *et al.* 2000, Sigurdson *et al.* 2005, Metzger *et al.* 2006, Taylor *et al.* 2009, Rose *et al.* 2012) and neuroblastoma patients treated with ^131^I-mIBG (Clement *et al.* 2015). Environmental radiation, such as occurred after the Chernobyl accident, significantly increases the risk of DTC in CYP (Nikiforov & Gnepp 1994). Endemic hypothyroidism secondary to iodine deficiency may also predispose to DTC (Santos *et al.* 2017).

### Differentiated thyroid cancer: assessment of CYP with thyroid enlargement

5. **Consider testing thyroid function in CYP presenting with a thyroid nodule or enlargement** *(Moderate Recommendation, Low-Quality Evidence, GDG Consensus)*

Assessment of thyroid function is standard practice in CYP presenting with thyroid abnormalities such as a nodule or goitre, in order to diagnose hypo- or hyperthyroidism. However, most children with DTC are euthyroid at diagnosis (Papendieck *et al.* 2011, Suzuki *et al.* 2016). A correlation between higher TSH levels and risk of malignancy has been identified in one low and four very low-quality cohort studies of children with thyroid nodules (Chiu *et al.* 2012, Mussa *et al.* 2013, 2015, Papendieck *et al.* 2015, Ly *et al.* 2016) and between TSH levels and cervical lymph node metastases in a very low-quality cohort study of children diagnosed with differentiated thyroid carcinoma (Papendieck *et al.* 2011). In a CYP with a thyroid nodule found to have a low serum TSH, thyroid scintigraphy Iodine 123 or Technetium 99m pertechnetate can help to determine whether the nodule contains autonomously functioning tissue (Niedziela *et al.* 2002). Scintigraphy should be reserved only for children whose serum TSH is low. The majority of patients present with normal or high serum TSH thyroid, with ultrasound (US) being the optimal modality to confirm or refute the presence of thyroid nodule (see Recommendation 11).

6. **Consider measurement of thyroid autoantibodies in CYP with thyroid enlargement, including those undergoing investigation for a thyroid malignancy** *(Moderate Recommendation, Delphi Consensus 73%)*

Investigation of a CYP with thyroid enlargement will involve the measurement of thyroid autoantibodies to diagnose autoimmune disease. However, children with differentiated thyroid carcinoma may present with co-existing autoimmune thyroid disease (Lazar *et al.* 2009, Papendieck *et al.* 2011, Baş *et al.* 2012). Additionally, a high pre-operative thyroid autoantibody titre in an euthyroid patient indicates an increased risk of hypothyroidism post-surgery (Verloop *et al.* 2012).

7. **Do not routinely measure calcitonin in CYP undergoing investigation of thyroid abnormalities** *(Strong Recommendation, Delphi Consensus 91%)*

Calcitonin should be measured if a diagnosis of medullary thyroid cancer (MTC) is suspected or diagnosed, or if the patient has a genetic predisposition to MTC. Routine measurement of calcitonin in patients who present with a thyroid abnormality is not recommended by these Guidelines, in view of the very low likelihood of detecting MTC in the absence of other clinical indicators and risk of false-positive or indeterminate results, and the associated anxiety provoked by such results.

8. **Discuss CYP diagnosed with DTC in both the adult thyroid and paediatric/Teenager and Young Adult (TYA) multidisciplinary team (MDT)s, or in a properly constituted all-age thyroid MDT** *(Strong Recommendation, Delphi Consensus 87%)*

DTC is rare in children. The combination of specialist expertise in the diagnosis and treatment of thyroid cancer in the adult thyroid MDT, in addition to expertise in the care of CYP with malignancy in the paediatric/TYA MDT will provide the best care for young patients.

9. **Take a three-generation family history for relevant conditions in CYP with DTC** *(Strong Recommendation, High-Quality Evidence)*
10. **Refer to a clinical geneticist for all CYP who have syndromic features (see Table 1), a family history of DTC or a family history of syndromic features associated with DTC *(Strong Recommendation, High-Quality Evidence)***

**Table:** Genetic syndromes associated with differentiated thyroid cancer.

Syndrome	Germline pathogenic variant and mode of inheritance	Type of thyroid cancer	Syndromic features noted on clinical examination	Further clinical features
*PTEN* hamartoma tumour syndrome (Includes Cowden syndrome, Bannayan-Riley-Ruvalcaba syndrome and PTEN-related Proteus syndrome. These overlapping phenotypes are all known to be due to pathogenic *PTEN* variants)	*PTEN* (autosomal dominant)	Multinodular goitre, adenomatous nodules and follicular adenomas Papillary thyroid cancer (classical and follicular variant) Follicular thyroid cancer	Macrocephaly (OFC >97th centile) and dolichocephaly, learning difficulties, autism and developmental delay, lipomas, vascular features including haemangiomas and arteriovenous malformations, gingival hypertrophy, oral papillomas, facial papules, acral keratoses, palmoplantar keratosis, trichilemmomas, pigmented macules of the glans penis and overgrowth of tissues.	Benign and malignant tumours of the breast, colon, endometrium and kidney, adult Lhermitte-Duclos disease due to cerebellar dysplastic gangliocytoma.
Familial adenomatous polyposis (FAP) (includes Gardner syndrome and Turcot syndrome. These overlapping phenotypes are all known to be due to pathogenic *APC* variants)	*APC* (autosomal dominant, with 20% cases arising *de novo*)	Papillary thyroid cancer including cribiform pattern subtype	Congenital hypertrophy of the retinal pigment epithelium (CHRPE), congenital absence of teeth, delayed eruption of teeth, dentigerous cysts, supernumerary teeth, odontomas, epidermoid cysts, fibrous dysplasia of the skull, mandibular osteomas, fibromas, desmoid tumours and pilomatrixoma.	Hepatoblastoma, medulloblastoma, multiple adenomatous polyps throughout the gastrointestinal tract, principally affecting the colon with high likelihood of malignant transformation, as well as upper GI tract adenomas and adrenal adenomas.
Carney complex	*PRKAR1A* (autosomal dominant, with 30% cases arising *de novo*)	Papillary thyroid cancer, follicular thyroid cancer and follicular adenoma	Pale brown to black lentigines of skin, lips and oral mucosa, soft tissue myxomas, schwannomas and epithelioid-type blue nevi.	Benign adrenal tumours (primary pigmented nodular adrenocortical disease), pituitary tumours (often somatotropinomas), large cell calcifying Sertoli cell tumours, breast ductal adenoma, osteochondromyxoma and Psammomatous melanotic schwannoma of the nerve sheath.
DICER1	*DICER1* (autosomal dominant)	Multinodular goitre and papillary thyroid cancer	None	Pleuropulmonary blastoma, ovarian Sertoli-Leydig cell tumours, cystic nephroma, ciliary body medulloepithelioma, botryoid-type embryonal rhabdomyosarcoma, nasal chondromesenchymal hamartoma, pituitary blastoma, pineoblastoma, Wilms tumour and juvenile intestinal hamartomas.
Werner	*WRN* (autosomal recessive)	Papillary thyroid cancer, Follicular thyroid cancer and anaplastic thyroid cancer	Short stature (lack of pubertal growth spurt), cataracts, premature aging, tight atrophic skin, ulceration, hyperkeratosis, pigmentary alterations, regional subcutaneous atrophy, and characteristic ‘bird-like facies’, hypogonadism, secondary sexual underdevelopment, premature greying and thinning of scalp hair, pes planus and abnormal voice.	Malignant melanoma, meningioma, soft tissue sarcomas, leukaemia and pre-leukaemic conditions of the bone marrow, primary bone neoplasms, osteoporosis, soft tissue calcification, evidence of premature atherosclerosis and diabetes mellitus.

Approximately 5% of CYP with familial DTC will have an underlying syndromic genetic predisposition (Peiling Yang & Ngeow 2016). There is high-quality evidence for the association between certain syndromes and the development of DTC (Yamashita & Saenko 2007, Morrison & Atkinson 2009, Richards 2010, Lauper *et al.* 2013, Rutter *et al.* 2016) – Cowden syndrome (PTEN hamartoma syndrome) (Marsh *et al.* 1998, Ngeow *et al.* 2011, Smith *et al.* 2011), Familial Adenomatosis Polyposis (FAP) (Kennedy *et al.* 2014), Carney complex (Stratakis *et al.* 1997) and Multinodular goitre families (DICER1 pathogenic variants) (Rio Frio *et al.* 2011, De Kock *et al.* 2014, Stewart *et al.* 2019). Putative low-moderate penetrant NMTC susceptibility genes have recently been described in dominant papillary thyroid carcinoma (PTC) families (Fallah *et al.* 2013, Zivaljevic *et al.* 2013, Peiling Yang & Ngeow 2016). Werner syndrome, a rare autosomal recessive disease, is known to predispose to DTC (median age of onset 20 years) (Lauper *et al.* 2013). A CYP with DTC and syndromic features and no family history of the condition may represent a case of *de novo* presentation or somatic mosaicism. A *de novo* presentation of FAP is known to occur in 20–25% of cases (Aretz *et al.* 2004).

Clinical examination of a CYP with DTC should include measurement of their height, weight and head circumference (maximum occipito-frontal diameter), with the results plotted on an appropriate growth centile chart. The clinical features associated with rare syndromic forms of DTC predisposition are summarised in Table 1. Clinicians may be able to identify those patients with an underlying syndromic cause for their DTC by careful examination. However, as the penetrance of these features is variable and many clinical features will develop in older childhood or adulthood, the diagnosis may only become apparent if other family members are also examined. Family history should include details of previous genetic testing if cases of DTC or syndromic causes of DTC, multinodular goitre or thyroidectomy are identified in the patient’s relatives. All possible efforts should be made to establish the precise thyroid tumour pathology and that of other relevant tumours previously diagnosed in the family.

The UK Genomic Medicine service strongly supports diagnostic testing in the clinical setting, that is, within the paediatric endocrine, surgical or oncology teams as well as within the genetics service. There are now several genetic panels that can be tested for in patients with paediatric DTC or multinodular goitre within the current test directory (https://www.england.nhs.uk/publication/national-genomic-test-directories/ (v3 April 2022)). Therefore, in the United Kingdom, referral to the clinical genetics service is not a requirement to carry out genetic testing on children with DTC or multinodular goitre, and the MDT can direct early genetic testing with additional referral to clinical genetics if there are variants of interest identified.

### Differentiated thyroid cancer: imaging of CYP with thyroid enlargement

11. **Undertake neck US in all CYP with thyroid enlargement with normal thyroid function if malignancy is suspected** *(Strong Recommendation, High-Quality Evidence)*

**Figure 2:**
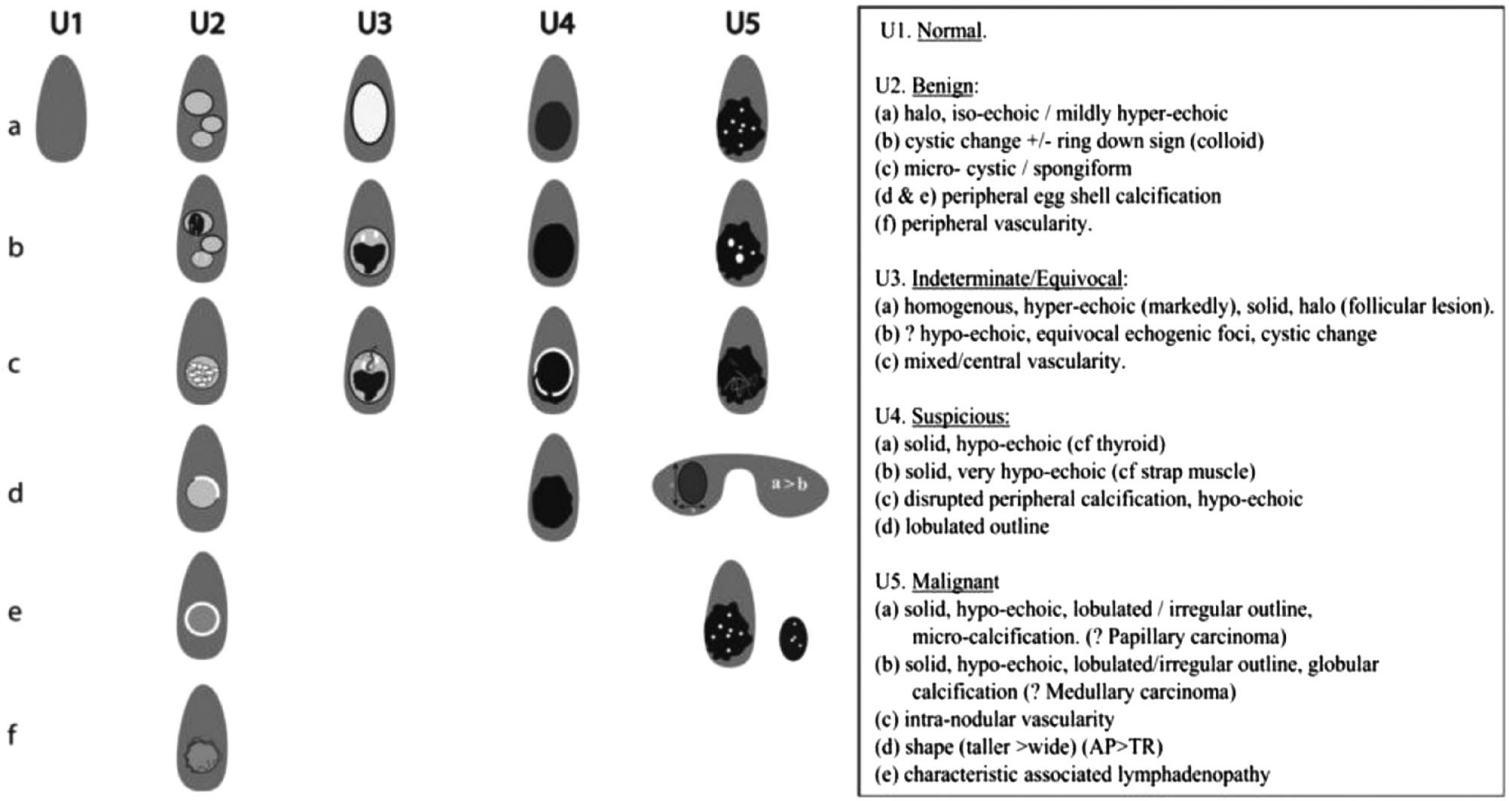
British Thyroid Association 2014 classification ultrasound scoring of thyroid nodules. Reproduced, with permission, from Perros *et al.* (2014). Copyright 2014 John Wiley and Sons.

US characteristics, as identified by an experienced head and neck or thyroid radiologist, are important in the differentiation of benign from malignant disease in CYP (Lyshchik *et al.* 2005, Corrias *et al.* 2010, Saavedra *et al.* 2011, Goldfarb *et al.* 2012, Gupta *et al.* 2013). US findings that predict an increased risk of thyroid cancer (Fig. 2) include solid nodules, nodules with internal calcification, the presence of enlarged or abnormal lymph node/s, irregular nodule margins, nodules that are taller than wide on transverse view (Al Nofal *et al.* 2016), hypoechoic nodules and increased intranodular blood flow on colour Doppler (Mussa *et al.* 2015, Koltin *et al.* 2016, Moudgil *et al.* 2016). US can assist to distinguish enlarged reactive lymph nodes, preventing their unnecessary biopsy, from DTC lymph node metastases, which have characteristic US appearances (Abbasian Ardakani *et al.* 2018).

The differential diagnosis of multinodular goitre in CYP includes diffusely infiltrating papillary thyroid cancer that presents with enlargement of a lobe or the entire thyroid, very frequently associated with microcalcifications on neck US (Wang *et al.* 2022). Multinodular thyroid enlargement in CYP, especially if associated with palpable cervical lymph nodes, in a CYP with normal thyroid function therefore requires investigation by neck US.

The benefits of this recommendation are that the US provides excellent visualisation of structures in the neck without exposure to further ionising radiation and facilitates cytological examination of abnormal findings. There are no anticipated risks or side effects of following this recommendation.

12. **Report neck US using the U1–U5 US reporting system as recommended by the British Thyroid Association** *(Strong Recommendation, Moderate Quality Evidence, GDG Consensus)*

The use of the U1–U5 scoring/grading system is recommended for assessing the risk of malignancy in adults with thyroid enlargement (Fig. 2) (Perros *et al.* 2014). The GDG agreed it was appropriate to apply the same system to CYP undergoing investigation for DTC.

### Differentiated thyroid cancer: pre-operative investigation of patients with known DTC

13. **Use US-guidance in CYP undergoing fine-needle aspiration (FNA) for the investigation of DTC** *(Strong Recommendation, High-Quality Evidence)*

The BTA and ATA paediatric guidelines both recommend US-guided fine-needle aspiration cytology (FNAC) to minimise rates of indeterminate samples and reduce rates of unsatisfactory samples (Izquierdo *et al.* 2009, Perros *et al.* 2014, Francis *et al.* 2015). As the use of US-guided FNAC is well established in the adult population to accurately identify targets for cytological assessment, it is logical to apply US-guided FNAC in CYP.

14. **Offer sedation or general anaesthesia if FNA is required, depending on the needs of the individual child** *(Moderate Recommendation, Low-Quality Evidence, GDG Consensus)*

There is no literature to clearly address the question of age cut-off for tolerating FNAC under local anaesthesia. In children under 10 years of age, and/ or in CYP who are unable to tolerate FNAC under local anaesthesia for other reasons, who require thyroid/neck FNA, sedation or general anaesthesia should be considered (Redlich *et al.* 2012). While general anaesthesia in a specialist centre carries a very low risk of harm, the associated risks and potential side effects should be discussed with patients and families as for any other procedure requiring general anaesthesia.

15. **Undertake US-guided FNA on a thyroid nodule reported on US as U3- indeterminate, U4- suspicious or U5- malignant** *(Strong Recommendation, High-Quality Evidence)*

The utility of pre-operative FNAC of thyroid nodules in CYP in differentiating benign and malignant disease has been evaluated via multiple studies including one meta-analysis (Raab *et al.* 1995, Khurana *et al.* 1999, Hosler *et al.* 2006, Stevens *et al.* 2009, Altincik *et al.* 2010, Bargren *et al.* 2010, Hoperia *et al.* 2010, Kapila *et al.* 2010, Gutnick *et al.* 2012, Monaco *et al.* 2012, Redlich *et al.* 2012, Cole & Wu 2014, Rossi *et al.* 2014, Buryk *et al.* 2015, Norlen *et al.* 2015, Partyka *et al.* 2016, Trahan *et al.* 2016). In CYP, pre-operative FNAC has high sensitivity and specificity, as well as high-positive predictive and negative predictive values for the diagnosis of malignancy (Stevens *et al.* 2009, Moudgil *et al.* 2016), although there is variability in the interpretation of cytopathology findings between institutions (Jia *et al.* 2021, Vuong *et al.* 2021, Cherella *et al.* 2022). In CYP, as in adults, the finding of follicular cytology on FNAC is not able to distinguish follicular adenoma from follicular carcinoma (Smith *et al.* 2013), and thus diagnostic hemithyroidectomy will be required. Observation with an interval US as an alternative to FNAC can be offered if a solitary sub-centimetre U3 indeterminate lesion is identified.

16. **Report cytology samples using the Thy 1–5 grading system. Thyroid cytology must be reported by a cytopathologist with expertise in thyroid disease** *(Strong Recommendation, Delphi Consensus 93%)*

The Royal College of Pathology document recommends thyroid cytology be reported in prose, together with an allocated Thy category (Thy 1−Thy 5) (https://www.rcpath.org/resourceLibrary/g089-guidancereportingthyroidcytology-jan16.htmln) (Table 2). If FNA sampling is inadequate (Thy1), in the absence of clinical or radiological features of concern, US-guided FNA may be repeated between 3 and 6 months (Perros *et al.* 2014, Francis *et al.* 2015).

**Table 2:** Royal College of Pathologists thyroid cytology reporting system.

Thyroid 1	Non-diagnostic for cytological diagnosis
Thyroid 1c	Cystic lesion
Thyroid 2	Non neoplastic
Thyroid 2c	Non neoplastic, cystic lesion
Thyroid 3a	Neoplasm possible-atypia/non-diagnostic
Thyroid 3f	Neoplasm possible, suggesting follicular neoplasm
Thyroid 4	Suspicious of malignancy
Thyroid 5	Malignant

17. **Consider diagnostic hemi-thyroidectomy in CYP if there is a discrepancy between clinical and/or radiological features and cytology** *(Moderate Recommendation, Delphi Consensus 71%)*

Benign cytology (Thy2) that does not correlate with clinical/radiological findings of concern, or repeated findings of indeterminate cytology (Thy3f), indicates a need for diagnostic surgery (hemithyroidectomy) (Lale *et al.* 2015). If cytology demonstrates Thy3a, but clinical/radiological suspicion is assessed by the MDT to be low, US-guided FNA may be repeated between 3 and 6 months. This recommendation is supported by adult thyroid cancer guidelines (ATA & BTA) and ATA guidelines for children with thyroid nodules (Perros *et al.* 2014, Francis *et al.* 2015, Haugen *et al.* 2016). The MDT will need to review the management of patients with cytology that repeatedly demonstrates Thy3a on an individualised basis. There is growing evidence of the potential utility of somatic oncogene analysis to aid management decisions in FNA with indeterminate cytology (Mostoufi-Moab *et al.* 2018, Pekova *et al.* 2021, Mollen *et al.* 2022) (see Recommendation 34).

18. **Consider the use of MRI neck with contrast prior to surgery in CYP with known DTC with evidence of local invasion or lymph node metastases** *(Moderate Recommendation, Low-Quality Evidence, GDG Consensus)*

In CYP with DTC and features suggestive of extrathyroidal invasion, lymph node involvement is common (Jarzab *et al.* 2000, Koo *et al.* 2009, Hay *et al.* 2010). In the presence of clinically or radiologically detected nodal disease, for example, bilateral cervical lymphadenopathy, especially with the involvement of lower cervical and supraclavicular nodes, a large or fixed thyroid swelling, or symptoms of vocal cord paralysis (stridor, hoarse voice), further imaging with MRI contrast should be performed pre-operatively. This is particularly due to the importance of imaging the lateral neck and upper mediastinum, as knowledge of the extent of lymph node metastases is vital prior to surgery. Furthermore, local invasion of a tumour into local structures (trachea, oesophagus), while rare, is essential to guide the surgery (e.g., length of surgery, appropriate specialist backup).

Although the incidence of lung metastases in CYP presenting with DTC is much higher than in adults at approximately 12–23% (Vassilopouloii-Sellin *et al.* 1993, Bal *et al.* 2004, Leboulleux *et al.* 2005, Vali *et al.* 2015, De Jong *et al.* 2021, Nies *et al.* 2021), these will be accurately imaged at the staging radioiodine scan (Kim *et al.* 2011, Mostafa *et al.* 2016, Jiang *et al.* 2021), which now includes a SPECT/CT as well as planar scintigraphy as a matter of course. Rarely, large volume metastases visible on a plain chest radiograph may require cover with steroid therapy to avoid issues with flare/oedema with RAI (Fard-Esfahani *et al.* 2014).

19. **Consider the use of pre-operative laryngoscopy to assess vocal cord function in CYP with DTC who have voice symptoms or extra-thyroidal disease or who have had previous neck surgery** *(Moderate Recommendation, Moderate Quality Evidence)*

The Clinical Practice Guideline on improving voice outcomes after thyroid surgery (Chandrasekhar *et al.* 2013) recommends pre-operative examination of vocal fold mobility in patients with a normal voice when there is thyroid cancer with suspected extra-thyroidal extension or prior neck surgery. Children under the age of 18 years were specifically excluded from the target population of the guideline although the authors state that many of the findings might be applicable. The NCCN and the BTA have recommended pre-operative laryngeal examination for patients with proven or suspected thyroid malignancy.

Reasons given to support the use of pre-operative laryngoscopy are (i) to confirm in the event of a finding of post-operative vocal cord palsy (VCP) that it is indeed newly caused by the procedure and (ii) when a pre-operative VCP is identified, to raise awareness in the patient and surgeon of the risk of bilateral palsy if contralateral surgery is planned. Routine laryngoscopy prior to thyroid surgery performed on adult patients identified a 2.3–2.8% prevalence of pre-operative VCP (Lang *et al.* 2014, Heikkinen *et al.* 2019).

Studies that include routine pre-operative (and post-operative) laryngoscopy in paediatric patients prior to thyroidectomy are rare. Machens *et al*. do not report finding VCP on routine pre-operative laryngoscopy in 230 surgically treated CYP (Machens *et al.* 2016). A recent study of complication rates after 464 paediatric thyroidectomies reports pre-operative laryngoscopy was performed on patients due to undergoing completion surgery or on those with hoarseness or dysphagia pre-operatively (Baumgarten *et al.* 2019). The patient group included 178 operations for malignant disease. The paper does not include the number of pre-operative laryngoscopies performed or a finding of pre-operative VCP.

There is increasing evidence in adults that trans-laryngeal US is as effective as laryngoscopy in screening patients pre-operatively for VCP, especially if there is no clinical suspicion (Gambardella *et al.* 2020). It has very high sensitivity and specificity and is likely to be better tolerated than laryngoscopy, especially in younger children. Moreover, there is recent evidence in favour of the use of intra-operative neuromonitoring, particularly if continuous, to reduce the risk of VCP in CYP (Ritter *et al.* 2021, Schneider *et al.* 2021).

### Differentiated thyroid cancer: surgical management

20. **Surgery in CYP with DTC must be undertaken by a high-volume thyroid surgeon (>30 cervical endocrine procedures per year in adults and children) with collaborative care between adult and paediatric surgeons** *(Strong Recommendation, Moderate Quality Evidence, GDG Consensus)*

Low-quality evidence from the United States indicates that high-volume surgeons (>30 cervical endocrine procedures per year in adults and children) have the best outcomes for CYP with DTC (Sosa *et al.* 2008, Burke *et al.* 2012, Breuer *et al.* 2013, Al-Qurayshi *et al.* 2016, Baumgarten *et al.* 2019, Maksimoski *et al.* 2022), especially in cases with active collaboration between endocrine and paediatric surgeons (Wood *et al.* 2011). In one US study, the case volume of the endocrine surgeons was an independent predictor of length of stay and costs, as well as reducing complications (Tuggle *et al.* 2008). Therefore, the risks of not following this recommendation are an increased incidence of short- and long-term complications of thyroid surgery (see Recommendation 22 for more details).

21. **The surgical team must be led by a thyroid MDT nominated all-age thyroid surgeon (with paediatric, adolescent and adult experience)** *(Strong Recommendation, Delphi Consensus 88%)*

The GDG agreed that a thyroid surgeon from the adult thyroid MDT nominated to operate on CYP with DTC should lead the designated surgical team. Due to the absence of UK evidence, this question was reviewed by a Delphi Consensus process.

22. **Discuss with patients and their carers the risks of thyroid surgery. These include hypocalcaemia (transient or permanent hypoparathyroidism), recurrent laryngeal nerve injury (transient or permanent), post-operative bleed requiring emergency surgery, wound infection and the need for lifelong levothyroxine. If lateral neck lymph node dissection is planned, risks include injury to the spinal accessory/phrenic/sympathetic nerves and lymphatic leak***(Strong Recommendation, Low-Quality Evidence, GDG Consensus)*

The most commonly reported complications in CYP undergoing thyroid surgery are post-operative hypocalcaemia associated with transient or permanent hypoparathyroidism and injury to the recurrent laryngeal nerve/s (RNL) (transient or permanent hoarse voice, aspiration on swallowing and post-operative chest infection) (Newman *et al.* 1998, van Santen *et al.* 2004, Morris *et al.* 2012). Post-operative bleeding and wound infection are rare (1%) (Hanba *et al.* 2017, Schneider *et al.* 2018). Damage to the spinal accessory/phrenic or sympathetic nerves and lymphatic leak are specific complications of lateral neck dissections. Additionally, there is a risk of injury to the external branch of the superior laryngeal nerve which produces more subtle voice changes such as weakness of voice or inability to raise or project the voice.

CYP have higher endocrine-specific complication rates than adults after thyroidectomy (Sosa *et al.* 2008). Younger children (aged 0–6 years) have higher complication rates than those in mid-childhood (7–12 years) and older CYP (13–17 years). Analysis of nationwide outcomes (from the United States) after thyroidectomy in children <1 year old revealed infection rates of 29.9%, respiratory sequelae in 35.2% and a high incidence of VCP of 14.3% (Hanba *et al.* 2017).

The incidence of transient hypoparathyroidism ranges from 4.5 to 35%, permanent hypoparathyroidism from 0 to 32%, RLN injury from 0 to 25%, tracheostomy 8% and Horner’s syndrome 2–8% (Newman *et al.* 1998, van Santen *et al.* 2004, Savio *et al.* 2005, Massimino *et al.* 2006, Burke *et al.* 2012, Morris *et al.* 2012).

Consent for surgery should be obtained as detailed in Section 3.5.1 – Establish and maintain partnerships with patients in ‘Good Surgical Practice’ (page 40–43, The Royal College of Surgeons of England 2014; https://www.rcseng.ac.uk/standards-and-research/gsp/).

23. **Record in the surgical operation note whether or not the RLNs are dissected and preserved, and the number of parathyroid glands identified, preserved and autografted** *(Strong Recommendation, Delphi Consensus 100%)*

Minimum standards for the content of operative notes are detailed in ‘Good Surgical Practice’ (page 21–22, The Royal College of Surgeons of England 2014; https://www.rcseng.ac.uk/standards-and-research/gsp/)

24. **Offer total thyroidectomy for surgical management of CYP with cytologically proven DTC** *(Moderate Recommendation, Moderate Quality Evidence)*

There are no randomised controlled trials to assess the optimal initial surgical management of DTC in CYP and there is no definitive evidence for the recommendation of more radical vs more conservative surgery. A systematic review (Jin *et al.* 2015) of seven retrospective case series (489 patients) found no evidence in CYP that overall survival is influenced by total thyroidectomy as compared to less extensive surgery such as hemithyroidectomy. Overall survival in 3861 cases from the National Cancer Database similarly failed to show an advantage from total thyroidectomy (Nice *et al.* 2015). However, selection bias may account for some of the lack of survival benefits from total thyroidectomy, and in some studies, follow-up may not have been sufficient to draw this conclusion (Nice *et al.* 2015). Nevertheless, there is some recent evidence to suggest there may be criteria by which to define very-low risk patients who may be candidates for lobectomy rather than total thyroidectomy (Kluijfhout *et al.* 2017).

*PTC*: In CYP, PTC is multifocal/bilateral in approximately 65 and 30% of patients, respectively. Lymph node metastasis at the time of diagnosis is evident in 40–90% and 20–30% present with distant metastasis (Dinauer *et al.* 2008, Rivkees *et al.* 2011). Total thyroidectomy performed by a high-volume surgeon is the optimal treatment for PTC in CYP (Welch Dinauer *et al.* 1999, Jarzab *et al.* 2000, Haveman *et al.* 2003, Kowalski *et al.* 2003, Hay *et al.* 2010, Astl *et al.* 2014). This surgical technique allows resection of the presenting macroscopic lesion in addition to the frequent microscopic foci of cancer, which can be found in the ipsilateral and contralateral lobes. Total or near-total thyroidectomy is advised to reduce the risk of local recurrence, to enable the use of radioiodine for ablation and therapy and allow subsequent monitoring of serum thyroglobulin (Bignol-Kologu *et al.* 2000, La Quaglia *et al.* 2000, Jarzab *et al.* 2005, Hogan *et al.* 2009, Mihailovic *et al.* 2014). As a significant number of CYP with PTC present with distant metastases, total or near-total thyroidectomy is advised in these patients as they will require radioiodine therapy. Management of patients who will require specific radiation protection (for example pregnant mothers) should be by an experienced paediatric molecular radiotherapy service.

Neck recurrence is more common in patients with lymph node involvement or multifocal disease at presentation, extrathyroidal invasion and distant metastases (Palmer *et al.* 2005, Demidchik *et al.* 2006, Spinelli *et al.* 2016). Low-quality studies have identified that lower recurrence rates and decreased need for reoperative surgery are associated with near/total thyroidectomy as compared to more conservative surgical approaches (Welch Dinauer *et al.* 1999, Jarzab *et al.* 2000, Haveman *et al.* 2003, Spinelli *et al.* 2004, Hay *et al.* 2010, Astl *et al.* 2014). Reoperative surgery is associated with higher rates of complications e.g. RLN injury and long-term hypoparathyroidism (Bignol-Kologu *et al.* 2000).

*Follicular variant papillary thyroid carcinoma (FVPTC)*: FVPTC in CYP has a low risk for bilateral disease and metastasis (Lerner & Goldfarb 2015*b*, Samuels *et al.* 2018). The surgical treatment of these patients should be decided on a case-by-case basis.

*Follicular thyroid cancer (FTC)*: In two retrospective series of CYP with FTC which included 50 patients (Enomoto *et al.* 2013, Spinelli *et al.* 2016) with a mean follow-up of 23.7 years and 6 years, respectively, cause-specific survival was 100% although the recurrent tumour was identified in three patients (two with distant metastases). Total thyroidectomy (single or two stages) was performed in 21 patients, external beam radiotherapy was used in eight cases and therapeutic RAI in eight cases. Only the patients in the more recent study received levothyroxine for TSH suppression. Total thyroidectomy was advised for patients with multifocal tumours, tumour diameter >4 cm, and for patients with >3 foci of vascular invasion, extrathyroidal tumour extension and distant metastases. Thyroid lobectomy and isthmectomy is appropriate treatment for patients with minimally invasive FTC and none of the above risk factors (Spinelli *et al.* 2019).

Data from observational studies suggest that the use of total thyroidectomy has increased from 50–60% to 85% in the last 20 years (Raval *et al.* 2010). Total thyroidectomy is more likely to be performed in high-volume centres (hospital factor) and if large tumours or nodal metastases are present at the time of resection (tumour factors) (Newman *et al.* 1998, Massimino *et al.* 2006, Raval *et al.* 2010, Burke *et al.* 2012).

Patients, parents and carers should be counselled that total thyroidectomy is associated with higher rates of permanent hypoparathyroidism and RLN injury (Newman *et al.* 1998, Welch Dinauer *et al.* 1999, Collini *et al.* 2006, Massimino *et al.* 2006, Canadian Pediatric Thyroid Nodule Study 2008, Enomoto *et al.* 2012) than lesser surgical procedures. This may be especially pertinent in young children (Scholz *et al.* 2011).

25. **Undertake therapeutic central neck dissection in CYP with DTC and confirmed neck lymph node metastases (N1)** *(Strong Recommendation, Moderate Quality Evidence, GDG Consensus)*

Increasing tumour size, extrathyroidal extension and multifocal disease are independent factors associated with nodal metastases in paediatric DTC. If these risk factors are present, children with DTC should undergo careful pre-operative evaluation for evidence of lateral cervical lymph node metastases, and the central compartment should be evaluated intraoperatively (Kim *et al.* 2017).

Cervical lymph node metastases (N1) are associated with reduced disease-free survival (Wada *et al.* 2009, Enomoto *et al.* 2012, Sugino *et al.* 2015, Spinelli *et al.* 2018). When therapeutic lymphadenectomy is performed, disease-free survival is equivalent to those patients without pre-operative evidence of lymph node disease (cN0) (Handkiewicz-Junak *et al.* 2007, Wada *et al.* 2009).

Central neck dissection (CND) has been demonstrated to reduce the rate of subsequent locoregional disease and increase the efficacy of radioiodine therapies in CYP with known lymph node metastases (Jarzab *et al.* 2000, Popovtzer *et al.* 2006, Handkiewicz-Junak *et al.* 2007). CND and the number of central nodes removed are significantly associated with permanent hypoparathyroidism in CYP with DTC (Ben Arush 2000, Machens *et al.* 2016) and it is important that the procedure is undertaken by an experienced surgeon.

Skip metastases (lateral neck node metastasis in the absence of central neck node involvement) in PTC are reported in adults in 7–20% (Chung *et al.* 2009, Park *et al.* 2012). On that basis, and that of low-quality evidence in CYP, CND should be considered when there is evidence of lateral neck node disease (Vassilopoulou-Sellin *et al.* 1998, Landau *et al.* 2000, Demidchik *et al.* 2006, Handkiewicz-Junak *et al.* 2007).

26. **Consider selective lymphadenectomy of the lateral compartment in CYP with DTC with confirmed lateral neck node metastases** *(Moderate Recommendation, Moderate-Quality Evidence)*

Moderate quality evidence suggests that lateral neck lymphadenectomy is associated with a significantly reduced rate of locoregional recurrence in cases of confirmed lymph node metastasis (Landau *et al.* 2000, Handkiewicz-Junak *et al.* 2007).

27. **Consider prophylactic central neck node dissection in CYP with Papillary Thyroid Carcinoma (PTC), particularly those with multifocal disease** *(Moderate Recommendation, Moderate Quality Evidence)*
28. **Do not consider prophylactic lateral neck lymphadenectomy in CYP with DTC** *(Moderate Recommendation, Low-Moderate Quality Evidence)*

CYP with DTC often have locoregional and distant spread at presentation, especially those with bilateral, multifocal or extrathyroidal disease (Ito *et al.* 2012, Jin *et al.* 2015, Balachandar *et al.* 2016, Jiang *et al.* 2016, Qu *et al.* 2016). However, it may be difficult to identify which cases are particularly at risk, in view of the observation that in CYP smaller tumour size may not correlate with reduced metastatic risk (Farahati *et al.* 1998, O’Gorman *et al.* 2010). Although the efficacy of radioiodine therapy can be increased after prophylactic node dissection, the MDT should consider carefully the morbidity of the procedure and potential benefits, particularly in younger children, with individualized decision-making (Machens *et al.* 2016). Recent evidence suggests there is no indication for prophylactic dissection in paediatric patients with FVPTC (Samuels *et al.* 2018).

29. **Monitor serum calcium levels after total/completion thyroidectomy in the peri- and post-operative period until levels are stable and within the normal range** *(Strong Recommendation, Low-Quality Evidence, GDG Consensus)*

**Figure 3:**
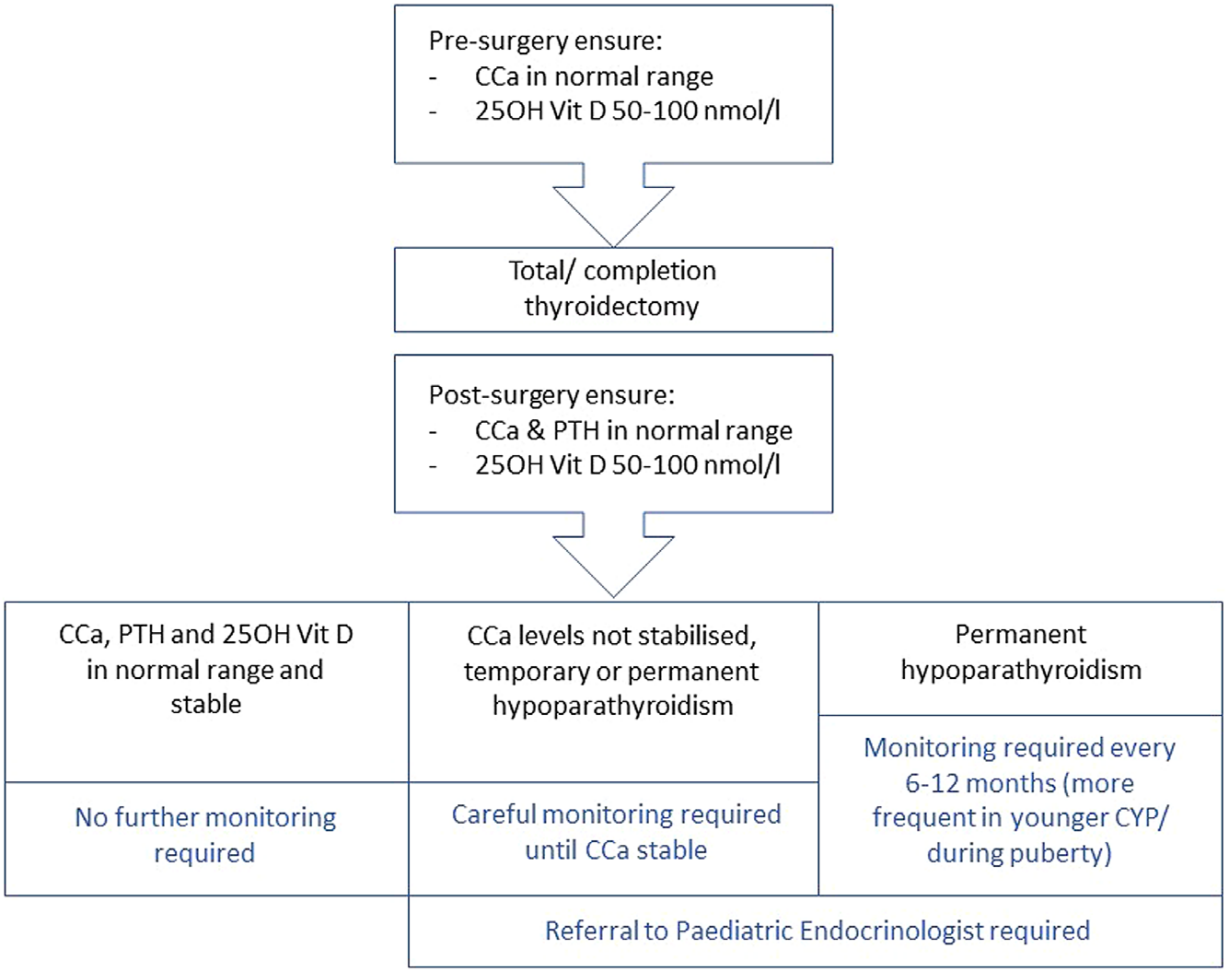
Calcium monitoring flowchart for total/completion thyroidectomy. CCa, corrected serum calcium; PTH, parathyroid hormone.

Hypocalcaemia consequent to transient or permanent hypoparathyroidism may follow thyroid surgery in CYP; monitoring of serum calcium and intact parathyroid hormone levels is advised in the peri- and post- operative periods (Astl *et al.* 2014, Freire *et al.* 2014, Patel *et al.* 2018). 25-hydroxy vitamin D levels should be measured prior to surgery, and treatment or supplementation should be given as appropriate (Fig. 3).

30. **Monitor calcium levels every 6–12 months in CYP with permanent hypoparathyroidism and stable calcium levels on treatment** *(Strong Recommendation, Delphi Consensus 92%)*
31. **Monitor calcium levels more frequently than every 6–12 months in younger patients and during puberty** *(Strong Recommendation, Delphi Consensus 92%)*

The literature search did not identify any published evidence to support the surveillance frequency of CYP with permanent hypoparathyroidism, with biochemical plasma and urine calcium monitoring, or with renal US to monitor for nephrocalcinosis. This was therefore reviewed by the Delphi Consensus group who advised 6–12 monthly assessments. More frequent monitoring is advised in younger CYP and during puberty (Fig. 3).

There is one study with low-quality evidence that calcium and vitamin D supplementation improves bone–muscle proportionality in boys (but not in girls) with hypoparathyroidism following total thyroidectomy (Handkiewicz-Junak *et al.* 2007). There was no consensus on the utility or frequency of DEXA scanning in this group. If permanent hypoparathyroidism is confirmed CYP should be referred to and regularly reviewed by a paediatric endocrinologist (Schneider *et al.* 2004). This care should be transitioned to an adult endocrinologist when appropriate.

32. **Undertake formal laryngeal examination in CYP who have a post-operative voice change following thyroidectomy and/or central neck dissection** *(Strong Recommendation, Low-Quality Evidence, Delphi Consensus 90%)*

The incidence of post-operative VCP may be underestimated unless routine evaluation is performed. Institutions that practice routine post-operative laryngoscopy report almost two times higher rates of cord palsy than those that do not (Bergenfelz *et al.* 2008). Studies that report routine post-operative vocal fold examination post thyroidectomy identify transient and permanent VCP in 5.2–12.6% and 1.1–3.3% of patients, respectively (Lo *et al.* 2000, Joliat *et al.* 2017, Heikkinen *et al.* 2019). Routine post thyroidectomy laryngoscopy in CYP has not been reported.

Studies of post thyroidectomy outcomes in the paediatric setting that did not include a routine examination of the larynx include 186 paediatric thyroidectomies over a 20-year period, in which there were three cases (1.6%) of temporary RLN injury, but none were permanent (Chen *et al.* 2015).

Intraoperative and/or post-operative concern for nerve injury resulted in laryngoscopy in 16 of 464 patients (median age 15 years: range 2–24 years) post-thyroidectomy (Baumgarten *et al.* 2019). Ten patients were identified with unilateral vocal cord paresis and one patient with bilateral paresis. Two patients had persistent unilateral RLN deficit 6 months post-operatively (0.4%). The ‘Kids Inpatient Database’ study on thyroidectomy patients aged 1–20 years identified vocal cord paralysis in 1.7% of >2000 patients over a 2-year period. In patients <1 year of age, the incidence of VCP was 14.3% (Hanba *et al.* 2017).

Guideline recommendations for the adult population state that the surgeon should document whether there has been a change in voice between 2 weeks and 2 months following thyroid surgery (Chandrasekhar *et al.* 2013, Perros *et al.* 2014, Haugen *et al.* 2016, Sinclair *et al.* 2016).

### Differentiated thyroid cancer: histology

33. **Report DTC in CYP using The Royal College of Pathologists data set for thyroid cancerhistopathology reports** *(Strong Recommendation, High-Quality Evidence)*

The Royal College of Pathologists’ data set for thyroid cancer should be used to report thyroid pathology in CYP (https://www.rcpath.org/resourceLibrary/g089-guidancereportingthyroidcytology-jan16.html; https://www.rcpath.org/profession/guidelines/cancer-datasets-and-tissue-pathways.html).

34. **Do not routinely use molecular genetic pathology testing (DNA/RNA tests looking for specific tumour mutations) in the assessment of histopathology samples for diagnostic purposes** *(Strong Recommendation, Delphi Consensus 93%)*

Molecular testing as an ancillary investigation for the diagnosis of thyroid nodules in the adult population is currently under investigation. There is increasing evidence that molecular testing for somatic oncogenes in CYP with DTC may provide additional diagnostic information to aid management (Mostoufi-Moab *et al.* 2018, Pekova *et al.* 2021, Mollen *et al.* 2022), but this should be as advised by histopathology experts within the context of the MDT.

The evidence for mutations in the paediatric population includes studies of alterations of BRAF, RAS, RET/PTC, PAX8/PPAR, ALK, p53, CLIP2 and the sodium-iodide symporter (Suchy *et al.* 1998, Beimfohr *et al.* 1999, Fenton *et al.* 2000, Patel 2002, Hess *et al.* 2011, Vriens *et al.* 2011, Sassolas *et al.* 2012, Leeman-Neill *et al.* 2013, Henke *et al.* 2014, Cordioli *et al.* 2016, Patel *et al.* 2018, Nies *et al.* 2021, Stosic *et al.* 2021). In children, the BRAFV600E mutation has been demonstrated with a variable prevalence in DTC, and it has not been associated with more aggressive tumour behaviour (Penko *et al.* 2005, Rosenbaum *et al.* 2005, Vriens *et al.* 2011, Finkelstein *et al.* 2012, Henke *et al.* 2014, Ballester *et al.* 2016, Gertz *et al.* 2016, Mostoufi-Moab *et al.* 2018). RET fusion oncogenes have wide-ranging prevalence reported in CYP with DTC (Nikiforov *et al.* 1997, Fenton *et al.* 2000, Ricarte-Filho *et al.* 2013, Ballester *et al.* 2016, Gertz *et al.* 2016), with differences in the specific types of rearrangements linked to radiation-induced and sporadic tumours (Nikiforov *et al.* 1997). Neurotrophic tyrosine kinase receptor (NTRK) fusion oncogenes are more common in paediatric than adult DTC and were found in recent cohort studies to be associated with a 100% probability of malignancy, as well as more extensive disease and aggressive pathology in CYP with DTC (Prasad *et al.* 2016, Pekova *et al.* 2021).

Potential therapeutic options for management of DTC in CYP are continuing to evolve, and the availability of molecular genetic testing, both for inherited germline variants, via panel testing (https://panelapp.genomicsengland.co.uk/panels/171/) and somatic oncogene sequencing for small sequence and structural variants on pathology samples (https://www.england.nhs.uk/publication/national-genomic-test-directories/) is expanding in the UK and elsewhere. While it is rare in CYP with DTC to require non-standard therapy, molecular genetic testing is likely to be increasingly used to direct targeted palliative therapies (see Recommendation 57). Future trials can also consider the inclusion of molecular subtypes into risk stratification (Franco *et al.* 2022).

35. **Report DTC in CYP using the TNM staging system** *(Strong Recommendation, Delphi Consensus 92%)*

The Royal College of Pathologists recommends that TNM version 8 is used for the classification of all thyroid cancer after January 2018 (https://www.rcpath.org/profession/guidelines/cancer-datasets-and-tissue-pathways.html). The TNM system describes well the extent of disease and predicts mortality in adults, but there is less good correlation in CYP (Zimmerman *et al.* 1988, Dottorini *et al.* 1997, Chow *et al.* 2004, Vaisman *et al.* 2011). In the absence of a CYP-specific scoring system, the GDG recommends following Royal College of Pathologists guidelines. It is important to note that age is an important predictor of CYP in that most, albeit low quality, studies show that young children have a worse prognosis compared to young adult or adolescent patients (Welch-Dinauer *et al.* 1998, Bal *et al.* 2001, Borson-Chazot *et al.* 2004, Powers *et al.* 2004, Palmer *et al.* 2005, Jang *et al.* 2012, Silva-Vieira *et al.* 2015).

TNM particularly lacks the accuracy to determine disease-free survival in low-risk CYP, due to the high rate of lymph node involvement in this group, leading to the risk of under-diagnosis and under-treatment of this group (Oommen *et al.* 2008, Wada *et al.* 2009).

36. **Consider recording a prognostic score (TNM) for CYP with DTC in the MDT** *(Moderate Recommendation, Delphi Consensus 73%)*

There is no literature evidence to support this best practice recommendation and it was therefore reviewed by a Delphi Consensus process and 73% of respondents supported this recommendation.

### Differentiated thyroid cancer: post-operative management

37. **Assess the need for and nature of further treatment in the MDT (adult thyroid cancer and paediatric or TYA MDTs or properly constituted all-age thyroid MDT). This will be determined by the histopathology, TNM stage and risk stratification** *(Strong Recommendation, Moderate-Quality Evidence, GDG Consensus)*

DTC in CYP is not a uniform entity, and the post-operative treatment course, assuming optimal surgery, needs to be individualised on a number of factors including age, stage and completeness of surgery. The patient’s post-operative histology should be therefore discussed at an age-appropriate thyroid cancer MDT.

The BTA guidelines (Perros *et al.* 2014) recommend an adaptation of the ATA risk grouping, while the ATA paediatric guidelines recommend a specific paediatric risk grouping (Francis *et al.* 2015). There is no evidence that one system is superior to the other.

While both the BTA and ATA offer guidelines for risk stratification after surgery, these are not identical. There is therefore an option for individual clinicians or for MDTs to use one rather than the other, or to take both into account, and offer choices to patients’ families. For example, a patient with pT1b pN1a M0 R0 disease may be classified as intermediate risk by the BTA system, and low risk by the ATA system. While radioactive iodine ablation has historically been recommended for the majority of DTC patients following surgery, there is recent evidence that some CYP with low-risk disease may not require it (Sohn *et al.* 2017, Sung *et al.* 2017). Further evidence is awaited from randomised trials. With this uncertainty, it would be reasonable to offer either radioactive iodine or close surveillance instead.

*Health benefits:* Children with DTC have specific paediatric needs and also specific disease-related needs. Expertise in both aspects is required. Their best care therefore requires expert input into both paediatric aspects of care and also disease-related aspects of care. A properly constituted all-age thyroid MDT will bring all the necessary expertise together in one setting, alternatively, this can be achieved by discussion in two different MDTs. There are no anticipated side effects or risks if this recommendation is followed. If children with DTC are considered only in an adult thyroid MDT, the focus may simply be on the disease, with neglect of paediatric aspects of care; if considered only in a paediatric or TYA MDT, then the disease-specific expertise may be lacking, so discussion in both is required to provide the best holistic care.

38. **Undertake radioiodine remnant ablation (RAA) according to post-operative risk assessment** *(Strong Recommendation, Delphi Consensus 87%)*

RRA is only applicable in those patients who have had either total thyroidectomy or lobectomy followed by completion thyroidectomy. Following total thyroidectomy, some radioiodine uptake is usually seen within the thyroid bed reflecting normal thyroid remnant tissue. Radioiodine-induced destruction of this remnant is known as ‘radioiodine remnant ablation’. This term should not be used to describe the subsequent treatment, which is referred to as radioiodine ‘therapy’ where the intention is to treat residual, recurrent or metastatic disease. The principles and procedures are similar for the administration of RRA or therapy (Perros *et al.* 2014).

The advantages of RRA are defined by the BTA (Perros *et al.* 2014) as follows:
  - Eradication of all residual thyroid cells post-operatively with subsequent reduced risk of local and distant tumour recurrence
  - Possible prolonged survival
  - Reassurance to patients is provided by the knowledge that serum thyroglobulin is undetectable and neck US or diagnostic iodine scan imaging is negative, implying that all thyroid tissue has been destroyed
  - Increased sensitivity of thyroglobulin monitoring, facilitating early detection of recurrent or metastatic disease
  - Increased sensitivity of subsequent iodine scanning if required
Following definitive surgery, CYP with DTC will fall into one of the following groups:
  - No indication for RRA – observation only
  - Uncertain indication for RRA, management discussed with patient and family, with an individualized approach and consideration for trial entry.
  - Definite indication for RRA and then, follow-up and dynamic risk stratification.
  - Definite indication for RRA and subsequent radioactive iodine therapy administration

**Figure 4:**
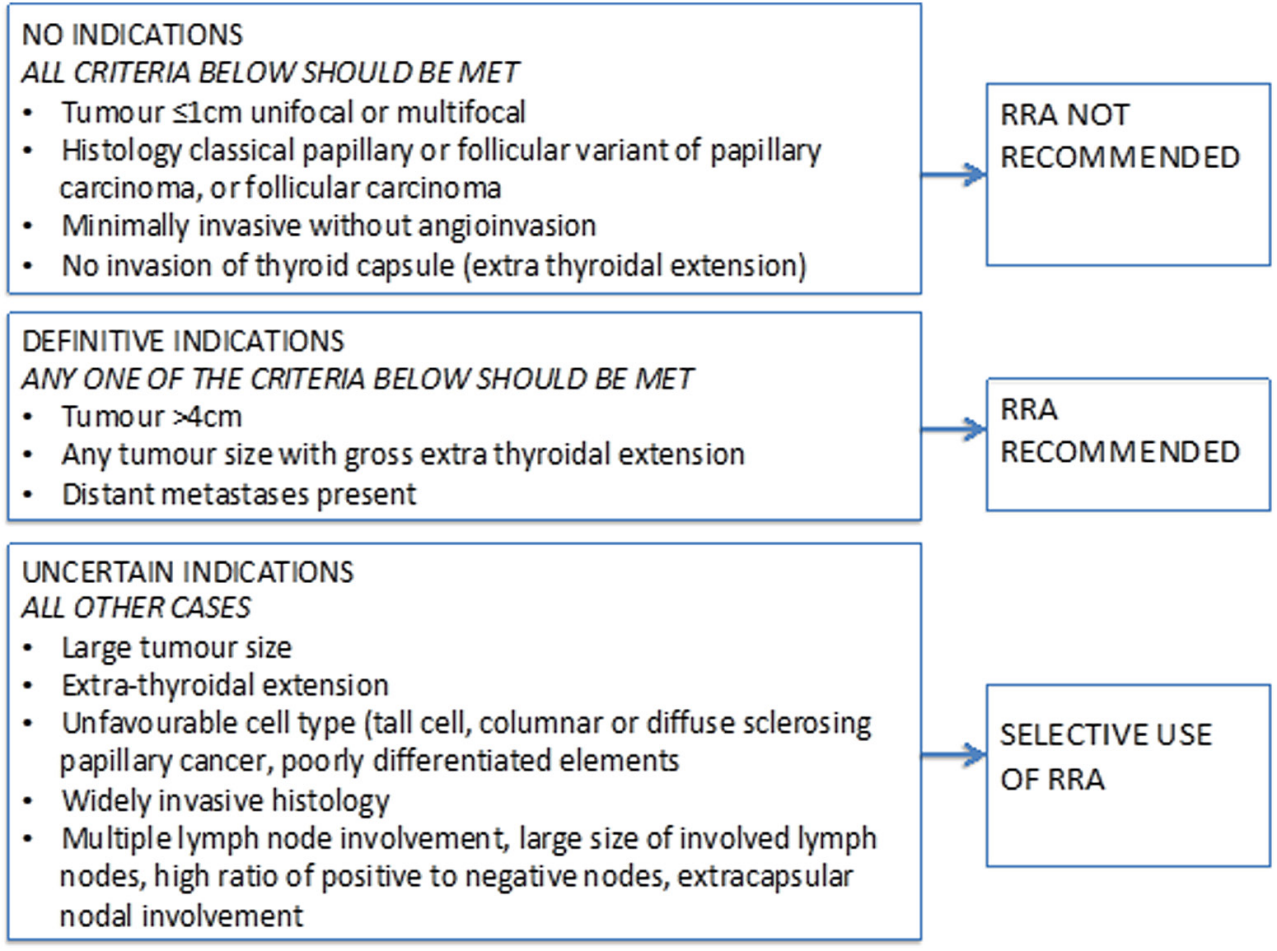
Decision-making flow chart for use of RRA, adapted from British Thyroid Association guidelines. RRA, radioiodine remnant ablation. Modified, with permission, from Perros *et al.* (2014). Copyright 2014 John Wiley and Sons.

The indications for RRA are mainly TNM stage dependent with additional information from the pathological risk factors also contributing to the decision-making (see Table 3). The BTA guidelines define the indications for RRA in adults with DTC into three groups based on available evidence; ‘no indication’, ‘definite indication’ and ‘uncertain indication’ (Fig. 4) (Perros *et al.* 2014).

**Table 3:** Comparison of risk stratification systems in British Thyroid Association (Perros *et al.* 2014) and American Thyroid Association Paediatric (Francis *et al.* 2015) guidelines for DTC.

Risk group	BTA-adapted ATA guidelines	ATA Paediatric guidelines
Low risk	No local or distant metastases All macroscopic tumour has been resected, that is, R0 or R1 resection (pathological definition) No tumour invasion of loco-regional tissues or structures The tumour does not have aggressive histology (tall cell, or columnar cell PTC, diffuse sclerosing PTC, poorly differentiated elements), or angioinvasion	Disease grossly confined to the thyroid with N0/Nx disease or patients with incidental N1a disease (microscopic metastasis to a small number of central neck lymph nodes)
Intermediate risk	Microscopic invasion of tumour into the perithyroidal soft tissues (T3) at initial surgery Cervical lymph node metastases (N1a or N1b) Tumour with aggressive histology (tall cell, or columnar cell PTC, diffuse sclerosing PTC, poorly differentiated elements) or angioinvasion	Extensive N1a or minimal N1b disease
High risk	Extra-thyroidal invasion Incomplete macroscopic tumour resection (R2) Distant metastases (M1)	Regionally extensive disease (extensive N1b) or locally invasive disease (T4 tumours), with or without distant metastasis

Most CYP with DTC present with locally advanced disease, with TNM tumour staging of T3 or T4, and/or N1 (Machens *et al.* 2010, Fridman *et al.* 2012, Markovina *et al.* 2014) and it is far less common to see microcarcinoma in the paediatric population (Lerner & Goldfarb 2015*a*). It is likely that most CYP with DTC will fall into the ‘definite’ or ‘uncertain’ indication groups on the basis of the BTA classification. Those falling into the uncertain group are likely to have RRA recommended following case review due to greater rates of high-risk pathological features (as described in Table 3) seen in this population.

Following RRA, previously unknown disease status, for example unsuspected metastatic disease, may be detected on the post-ablation scan, or an incomplete response on dynamic risk assessment may indicate the need for subsequent radioactive iodine therapy.

However, evidence to support the use of RRA, particularly in early intra-thyroidal disease (cT1-T2 cN0 cM0), is limited (Newman *et al.* 1998, Welch Dinauer *et al.* 1999, Jarzab *et al.* 2000, Chow *et al.* 2004, Demidchik *et al.* 2006, Handkiewicz-Junak *et al.* 2007). In CYP, there is low quality and conflicting evidence on the impact of RRA on disease-free survival (Powers *et al.* 2003). Evidence on whether RRA is associated with increased risk of secondary malignancy is to date conflicting (Newman *et al.* 1998, Welch Dinauer *et al.* 1999, Jarzab *et al.* 2000). Data from the Iodine or Not study in which patients aged 16 or older with low-risk thyroid cancer were randomised to receive RRA or not, will provide further guidance as to which patients can safely avoid RRA (Mallick *et al.* 2012*a*). Until this evidence is available, the standard UK approach in CYP is to treat any localised disease with definite or uncertain indications with RRA. However, there is room for individualisation of decision-making for the uncertain indications group (Perros *et al.* 2014) (see Fig. 4).

The ATA adult guidelines consider the use of post-operative thyroglobulin and US in relation to the need for RRA. This is on the basis that in adults, a post-treatment (surgery and RRA) stimulated thyroglobulin level <2 ng/mL and/or undetectable basal serum thyroglobulin measured with a highly sensitive assay are strong indicators of definitive cure and a very low risk of recurrent disease. A negative US scan adds to the sensitivity of this (Pacini *et al.* 2003, Smallridge *et al.* 2007, Spencer *et al.* 2010).

The optimal cut-off value or whether the stimulated or unstimulated thyroglobulin level should be used has not been defined, either in adults or children. It is critical to have a full discussion with the family to ensure all options and the risks and benefits of surveillance or treatment are conveyed.

The timing of RRA post-thyroidectomy is governed more by the availability of facilities and convenience for the patient than the biology of the tumour. There is no evidence that a delay of 1–2 months changes the outcome. Factors around schooling, childcare for siblings and preparing the child for a period of separation from family usually contribute to the selection of date for treatment. NHS cancer waiting targets stipulate treatment should be administered within 31 days of the decision to treat, but this is not based on the biology or outcomes of the disease.

While this recommendation is strongly made, it is important to acknowledge that decision-making is not always easy, especially when the indications for treatment are uncertain. Individual clinicians and families may have preconceived ideas, and families may have been influenced by what other clinicians have said, or by what they have read about the disease and its treatment. In these circumstances, when faced with the option of either radioactive iodine ablation or surveillance, it is the responsibility of the MDT to approach the family with options and to have a full and frank discussion of the uncertainties surrounding the evidence base. Some families will wish to be active participants in decision-making and will not wish to be told that they ‘must’ follow a certain course of action. Other families may feel unable to contribute to the decision-making, possibly for fear of making the wrong choice, and would like the clinician to make a definite decision. While families typically make the decisions on behalf of younger children, older teenagers who are Gillick competent, should be encouraged to express their opinions and contribute actively to decision-making where there are reasonable options.

39. **Radioiodine must be prescribed and administered by professionals experienced in the dosing and administration of radioiodine in CYP** *(Strong Recommendation, High-Quality Evidence)*

This recommendation ensures the least risk of the incorrect treatment being given. There is also a statutory responsibility in the prescription of radioactive iodine, as regulated by the Administration of Radioactive Substances Advisory Committee of the Department of Health and Social Care. There are no anticipated risks or side effects if this recommendation to limit prescribing and administration to experienced professionals is followed, but there is a significant risk of medication errors if this recommendation is not followed.

40. **Administer radioiodine to children less than 16 years within a paediatric oncology centre with 24 h medical and nursing care** *(Strong Recommendation, High-Quality Evidence)*

Children aged less than 16 years should receive radioiodine within a paediatric oncology centre with 24 h paediatric medical and nursing care. Older teenagers, from the age of 16 up to their 19th birthday, should receive care in a designated principal treatment centre for young people. From the age of 19–24 years (up to the 25th birthday), young adults should be offered radioiodine in a designated teenage and young adult cancer service but may elect for care in an adult environment that may be closer to home.

These recommendations are based on the Royal College of Radiologists Good Practice Guide for Paediatric Radiotherapy (https://www.rcr.ac.uk/system/files/publication/field_publication_files/bfco182_good_pract_paed_rt_second_ed.pdf), the NHS England: 2013/14 NHS Standard Contract for Cancer: Teenagers & Young Adults Section B Part 1 - service specifications (https://www.england.nhs.uk/wp-content/uploads/2013/09/b17.pdf), and also guidelines from the Intercollegiate Standing Committee on Nuclear Medicine (https://www.rcr.ac.uk/system/files/publication/field_publication_files/bfco199-icscnm-molecular-radiotherapy-guidance.pdf).

While there is a strong recommendation for younger children to be treated in a paediatric environment, older teenagers (over 18 years) should have the option to be treated in a specialist TYA centre with all the appropriate support for people in that age group or to be treated in an ‘adult’ centre which lacks TYA support.

41. **Consider adjustment of the radioiodine activity for thyroid ablation and therapy based on the size of the CYP** *(Moderate recommendation, Low-Quality Evidence, Delphi Consensus 78%)*

There are no standardised activities of RRA for children and there is no data to guide this. Following two randomised controlled trials in adult patients, HiLo (Mallick *et al.* 2012*b*) and Estimabl 1 (Schlumberger *et al.* 2012), the standard activity for RRA in adults has reduced from 3.7 to 1.1 GBq for patients at low risk of recurrence of their thyroid cancer. In higher-risk diseases, 3.7 GBq remains the standard administered activity.

Historically the same activity has been considered for children and adults, but some adaptation according to body weight, body surface area and age has been applied by many specialists (Handkiewicz-Junak *et al.* 2007, Pawelczak *et al.* 2010), and Delphi Consensus and stakeholder review both gave further support for this practice.

For subsequent radioactive iodine for therapy in the setting of persistent or recurrent disease the recommended empirical administered activity is 5.5 GBq in adults (Perros *et al.* 2014). As with RRA, there is no good data for the adjustment of activity for children. Although there is interest in the dosimetric prescription of radioiodine, there is no evidence to date to show superiority over empiric activity prescription. Reports have suggested that treatment with at least 200 MBq/kg (5.4 mCi/kg) is possible without a risk of exceeding bone marrow tolerance limits (Verburg *et al.* 2011). Many patients will tolerate much higher activities. However, in children with extensive metastatic disease whole-body dosimetry may be employed to ensure total blood dose does not exceed 2 Gy and that the whole-body retention at 48 h does not exceed 4.44 or 2.86 Gy (in the case of no or miliary lung metastases respectively) (Verburg *et al.* 2011, Verburg *et al.* 2013). CYP with DTC, particularly those with pulmonary metastases and coexisting micronodular disease, often show excellent RAI uptake and thus may be more sensitive to ^131^I therapy than adults (Xu *et al.* 2016). In conclusion, there is no high-quality evidence to advise for or against the prescription of radioiodine based on empiric activity or informed by dosimetry. GDG Consensus opinion suggests that experts prescribe RRA as an empiric activity and reserve dosimetric methodology for patients with extensive disease and repeated therapies in centres with this expertise.

For the majority of patients, there are very few complications of radioiodine. The long-term toxicities are believed to be related to the absorbed radiation dose as a function of the administered activity and therefore the risk of toxicity will increase with cumulative radiation dose and with cumulative administered radioiodine activity. The risks of RRA are relatively low following a single administration. While the majority of DTC patients are teenagers for whom the adult activity is appropriate, very small children may get a good response from a size-adjusted dose and avoid the risks of radiation exposure beyond that which is necessary. There are no anticipated risks or side effects if this recommendation is followed.

42. **Perform a blood or urine pregnancy test on all post-menarchal female patients prior to radioactive iodine administration** *(Strong Recommendation, Moderate-Quality Evidence, GDG Consensus)*

The Royal College of Radiologists Good Practice Guide for Paediatric Radiotherapy (https://www.rcr.ac.uk/system/files/publication/field_publication_files/bfco182_good_pract_paed_rt_second_ed.pdf) states that radioactive iodine may not be administered to pregnant women. Post-pubertal girls should be advised to avoid pregnancy for 6 months after radioiodine (although it may be wise to advise to wait until post-risk assessment at 9–12 months as this will determine response to ablation and whether further treatment is required).

43. **Consider fertility preservation with post-pubertal CYP if they are likely to receive more than two administrations of radioiodine (including the ablation administration)** *(Moderate Recommendation, Delphi Consensus 75%)*

A single ablation dose of radioiodine should have no effect on male or female fertility (Landau *et al.* 2000). Sperm banking should be discussed with all post-pubertal males if they are likely to receive more than two administrations of radioiodine (including the ablation administration) (Wallace 2011). Referral to fertility units for expert advice may be warranted in young women with multiple treatments with radioiodine. Delphi Consensus supported this recommendation, although emphasised that the risks of infertility are low in those patients not requiring high cumulative activities of radioiodine.

While it is important to discuss fertility preservation options with post-pubertal patients and their families, the absolute risk of fertility impairment is low, and following discussion, patients and families should have the option of undergoing fertility-preserving features, or not.

44. **Consider preparing CYP for radioactive iodine (RRA or therapy) with either thyroid hormone withdrawal or recombinant thyroid stimulating hormone** *(Moderate Recommendation, Moderate-Quality Evidence)*

Historically, radioactive iodine was always administered after thyroid hormone withdrawal. Typically, levothyroxine is stopped 28 days prior to radioactive iodine administration, and/or liothyronine 10 days before. Most children tolerate thyroid hormone withdrawal well and reach the target TSH of ≥30 IU for radioiodine administration without problems (Kuijt & Huang 2005). More recently, recombinant TSH (Thyrogen) has become the standard of care in adult practice (Mallick *et al.* 2012*b*). However, it is not licensed for use in children, and the data relating to its effectiveness have mostly related to the adult population. There is now increasing retrospective data suggesting the safety and efficacy of recombinant TSH in the paediatric population (Iorcansky *et al.* 2005, Luster *et al.* 2009, Rosario *et al.* 2012, Handkiewicz-Junak *et al.* 2015).

Uptake of radioactive iodine is poor without a raised TSH level. This recommendation ensures the maximum uptake of radioactive iodine and gives the best chance of successful treatment. While thyroid hormone withdrawal results in a feeling of tiredness and lethargy and may be associated with abnormal kidney function indicators on blood tests, thyrotropin alfa does not cause any significant side effects. However, two intramuscular injections are required.

45. **Advise a low-iodine diet prior to radioiodine treatment in CYP with DTC** *(Strong Recommendation, High-Quality Evidence, Delphi Consensus 100%)*

The promotion of low-iodine diets prior to radioiodine treatment in CYP with DTC is supported by adult (Perros *et al.* 2014) and paediatric guidelines (Pluijmen *et al.* 2003, Francis *et al.* 2015) and our Delphi Consensus. A high dietary iodine intake may reduce the therapeutic efficacy of radioactive iodine, and therefore a low iodine diet for 14 days before radioiodine treatment should maximise the chance of successful ablation or therapy (https://www.btf-thyroid.org/low-iodine-diet).

46. **Perform a whole-body iodine 131 scan following RRA** *(Strong Recommendation, High-Quality Evidence)*

Following RRA, a whole-body iodine 131 scan (preferably SPECT/CT with the CT component focusing on the neck and thorax, and any other body areas with possible abnormal uptake on planar scans) is indicated (Bal *et al.* 2004, Kim *et al.* 2011). This scan permits further staging of the disease, which may identify the presence of unsuspected distant metastases requiring subsequent therapy (Mostafa *et al.* 2016, Jiang *et al.* 2021). This will demonstrate localisation of uptake in the thyroid bed, thyroglossal tract remnants, cervical lymph nodes and pulmonary or other distant metastases. This is therefore an essential investigation for the complete staging of the disease and to predict prognosis. There are no side effects or risks anticipated if this recommendation is followed. There is a risk of missing metastatic disease if the recommendation is not followed.

47. **Investigate residual disease on post-RRA 131 scan with additional imaging. Further management will be determined by imaging findings***(Strong Recommendation, High-Quality Evidence)*

Neck uptake within cervical lymph nodes requires anatomical imaging to localise uptake with SPECT CT/US/MRI as available to assess for macroscopic disease (Antonelli *et al.* 2003, Bal *et al.* 2004, Kim *et al.* 2011). If residual disease or distant metastases are identified, the CYP should be referred back to the MDT for consideration of further treatment, surgical or RAI. All other patients will then proceed to dynamic risk stratification (see the subsequent paragraphs).

### Differentiated thyroid cancer: follow-up

48. **Perform a dynamic risk assessment following RRA. Use this to guide further management and ongoing follow-up** *(Strong Recommendation, High-Quality Evidence)*

At 9–12 months post-surgery and RRA, response to treatment should be assessed. If the suppressed thyroglobulin is undetectable, an US of the thyroid bed and bilateral neck together with the thyroglobulin following TSH stimulation should be performed. This is known as dynamic risk stratification, as recommended in the BTA and ATA Paediatrics guidelines (Perros *et al.* 2014, Francis *et al.* 2015). This assesses the response to treatment in low-risk diseases. There is very low-quality evidence that recombinant human TSH-stimulated thyroglobulin level is useful for disease surveillance in CYP (Hoe *et al.* 2006).

Patients are allocated to one of three groups following dynamic risk stratification: excellent response (no evidence of disease), indeterminate response (biochemical evidence of disease only) and incomplete response (imaging/structural evidence of disease with or without biochemical evidence) (see Table 4). These investigations allow an assessment of the completeness of ablation and facilitate allocation to a risk group for purposes of deciding the level of TSH suppression required and the frequency and intensity of follow-up going forward. There are no anticipated side effects or risks if this recommendation is followed. If the recommendation is not followed, patients may be under-treated, with the risk of disease progression or overtreated with the risk of treatment-related morbidity.

**Table 4:** Follow-up schedule for management of CYP with DTC.

Baseline risk group	Dynamic risk assessment		
	Excellent response	Indeterminate response	Incomplete response
Low-risk	(a) 6–12 monthly follow-up. (b) At least 5 years. (c) Clinic visit, TFTs and Tg. No routine imaging. (d) TSH in normal range.	(a) 6 monthly follow-up. (b) At least 5 years – possibly longer depending on imaging and Tg trend. Consider repeating DRA at 5 years. (c) Clinic visit, TFTs and Tg. Repeat US initially 6 monthly if abnormal – increasing to annually if stable over time. (d) TSH suppressed for at least 5 years.	Consider further treatment in MDT depending on DRA findings. If active surveillance chosen over further treatment: (a) 3–6 monthly follow-up. (b) At least 5 years – more likely longer depending on imaging and Tg trend. Consider repeating DRA at 5 years. (c) Clinic visit, TFTs and Tg. Repeat US initially 6 monthly if abnormal – increasing to annually if stable over time. (d) TSH suppressed indefinitely, unless repeat assessment shows improvement.
Intermediate risk	(a) 6 monthly follow-up. (b) at least 5 years. (c) Clinic visit, TFTs and Tg. Annual US. (d) TSH suppression may be slightly relaxed: target low-normal range.	(a) 6 monthly follow-up. (b) at least 5 years – possibly longer depending on imaging and Tg trend. Consider repeating DRA at 5 years. (c) Clinic visit, TFTs and Tg. Repeat US initially 6 monthly if abnormal – increasing to annually if stable over time. (d) TSH suppressed for at least 5 years.	Consider further treatment in MDT. depending on DRA findings. If active surveillance chosen over further treatment: (a) 3–6 monthly follow-up. (b) At least 5 years – more likely longer depending on imaging and Tg trend. Consider repeating DRA at 5 years. (c) Clinic visit, TFTs and Tg. Repeat US initially 6 monthly if abnormal – increasing to annually if stable over time. (d) TSH suppressed indefinitely, unless repeat assessment shows improvement.
High-risk	(a) 3–6 monthly follow-up. (b) At least 10 years. (c) Clinic visit, TFTs and Tg. US 6 monthly for 2 years, then annually to 5 years. (d) TSH <0.1 to 10 years.	(a) 3–6 monthly follow-up. (b) At least 10 years – possibly longer depending on imaging and Tg trend. Consider repeating DRA at 5 years. (c) Clinic visit, TFTs and Tg. Repeat US initially 6 monthly – increasing to annually if stable over time. (d) TSH <0.1 to 10 years.	Consider further treatment in MDT depending on DRA findings. If active surveillance chosen over further treatment: (a) 3–6 monthly follow-up. (b) At least 10 years, probably lifelong. (c) Clinic visit, TFTs and Tg. Repeat US initially 6 monthly if abnormal – increasing to annually if stable over time. Other imaging e.g. CT chest may need to be repeated periodically if clinically indicated. (d) TSH <0.1 to at least 10 years, consider need for life-long suppression if there is still evidence of controlled disease.

DRA, dynamic risk assessment.

49. **Use neck US as the first-line imaging modality for post-operative follow-up of CYP with DTC** *(Strong Recommendation, Moderate-Quality Evidence, GDG Consensus)*

In CYP with DTC, US is sufficient for evaluation of loco-regional involvement in follow-up (Vali *et al.* 2015), with sensitivity of 85.7%, specificity of 89.4%, negative predictive value of 94.4% and positive predictive value of 75%. Neck US can be used to pinpoint the anatomic site of lymph node metastases (Antonelli *et al.* 2003). In adults, the combination of neck US and FNAC has been shown to detect cases of lymph node metastasis and local recurrence not found by whole-body scan or serum thyroglobulin determination (Durante *et al.* 2013). Neck US is also useful, particularly in young children, post-operatively to help differentiate pathological from reactive lymph nodes.

50. **Measure thyroglobulin antibody in conjunction with serum thyroglobulin** *(Strong Recommendation, High-Quality Evidence)*

Even very low thyroglobulin antibody concentrations can interfere with assay results and thyroglobulin antibodies are found in up to 25% of adult patients (Spencer *et al.* 1998, Vali *et al.* 2015, Haugen *et al.* 2016). Each specimen sent for thyroglobulin measurement requires concomitant thyroglobulin antibody testing because thyroglobulin antibody status can change over time. As variability exists between different thyroglobulin assays, there is a need to use the same assay for serial measurements. Thyroglobulin antibody titres can also correlate with disease burden.

51. **Use the baseline risk grouping and subsequent dynamic risk stratification to determine the frequency and duration of follow-up** *(Strong Recommendation, Moderate-Quality Evidence, GDG Consensus)*

There is a lack of high-level paediatric evidence on which to make clear-cut and highly detailed follow-up recommendations, but it is reasonable and pragmatic to base the long-term follow-up schedule of CYP evidence from adults with DTC. Prognostic stratification will be based on both the baseline risk group and also the results of dynamic risk assessment after treatment (see Table 4). CYP will be followed up until transition to adult services, and for low-risk adult patients, a decision can be taken about discharge to primary care. Life-long follow-up is advised in high-risk groups given that recurrence of DTC can occur 40 years after the initial disease (Landau *et al.* 2000, Hay *et al.* 2010).

The suggested follow-up schedule is given in Table 4, modified from BTA (Perros *et al.* 2014) and ATA Paediatrics (Francis *et al.* 2015) guidelines and recent review (Lee *et al.* 2019). Follow-up of patients will be in one of the three groups. These are, as above, no evidence of disease (excellent response), biochemical evidence of disease only (indeterminate response), and imaging/structural evidence of disease with or without biochemical evidence (incomplete response). The purpose of surveillance is to detect evidence of relapse/progression at a time point when further intervention may be of value and to ensure TSH levels are optimised to reduce the risk of recurrence.

If no structural disease is present and stimulated thyroglobulin is not detectable, this represents an excellent response to treatment, and follow-up intervals can be extended to 6 months during childhood (92% agreement on Delphi Consensus) and relaxation of TSH suppression may be considered (as per Table 4).

In patients with low-level TSH-stimulated thyroglobulin (<10 ng/mL), continued follow-up with serial TSH-suppressed thyroglobulin is indicated. The role of imaging with US in this situation is unclear, and this should be an individualised decision by the treating clinician.

If the unstimulated thyroglobulin is detectable, an US should be performed to localise persistent disease that may be surgically resectable (Vali *et al.* 2015). If no structural disease is present, a therapy dose of RAI may be considered (Francis *et al.* 2015).

Following this recommendation allows for personalisation of care, based on the extent of disease at the time of diagnosis and the response to treatment. This facilitates individualised follow-up schedules, which ensure those at the highest risk are followed more intensively to detect progression, while those at low risk are spared unnecessary hospital visits.

### Differentiated thyroid cancer: metastatic, recurrent or persistent disease

52. **Use serial thyroglobulin measurement with additional imaging if required to monitor CYP with DTC** *(Strong Recommendation, Moderate-Quality Evidence, GDG Consensus)*

Serum thyroglobulin may continue to be detectable following RRA but may decline over time without additional therapy (Dottorini *et al.* 1997, Biko *et al.* 2011). A rise in thyroglobulin or thyroglobulin antibodies should trigger initial confirmation with repeat measurement within 2 months, followed by investigation with US of the thyroid bed and neck to exclude disease recurrence (Kirk *et al.* 1992, Xu *et al.* 2016). If neck US is normal, additional imaging such as CT or MRI can be considered to look for distant metastatic disease, especially lung metastases, if thyroglobulin levels suggest the disease may be present at a distant site. 123-I whole body scintigraphy and SPECT/CT may be added if the thyroglobulin measurement is considered to be unreliable because of a high antibody titre (Kim *et al.* 2011).

Sometimes the low administered activity of 123-I used for diagnostic imaging, and the resolution of the imaging techniques used, may result in small-volume disease being overlooked or mistaken for being iodine resistant. The use of 18F-FDG PET/CT may be a helpful additional investigation as it has high diagnostic accuracy for the detection of recurrent and/or metastatic diseases in DTC patients with thyroglobulin elevation and negative iodine scintigraphy (Larg *et al.* 2019, Qichang *et al.* 2019, Wang *et al.* 2021).

53. **Consider further surgical resection for persistent local structural disease** *(Moderate Recommendation, GDG Consensus)*

Structural disease refers to a definite abnormality on imaging, identifying that cancer has infiltrated anatomical structures such as jugular vein, trachea or oesophagus, or metastatic lymph nodes. If structural disease is detected on the neck US, MDT discussion about the role of further surgery is recommended.

54. **Consider therapeutic radioiodine after further surgical resection** *(Moderate Recommendation, Moderate-Quality Evidence)*
55. **Administer radioiodine as first-line treatment for unresectable metastatic disease in CYP** *(Strong Recommendation, High-Quality Evidence)*

Therapeutic choices after further surgical resection should be individualised, discussed with the MDT and agreed upon with the patient/family who may have strong views. The use of radioiodine should be considered and would normally be regarded as indicated if, following surgery, there is imaging or biochemical evidence of residual disease which was not felt to be amenable to surgery; whereas in the absence of such evidence, a policy of careful observation might be regarded as a safe and more conservative approach, although there is no evidence to show that the use of radioactive iodine is necessarily wrong. Most DTC in this age group responds well to radioactive iodine therapy and repeated therapy doses of radioactive iodine can be used after further surgical resection and to treat metastatic disease as long as a response is seen (Biko *et al.* 2011, Verburg *et al.* 2013). It is rare for DTC in CYP to become radioiodine refractory.

56. **Consider chest imaging (chest X-ray or chest CT) in patients with high-risk disease or those with evidence of persistent or recurrent disease to diagnose and monitor metastatic lung disease** *(Moderate Recommendation, Moderate-Quality Evidence)*

Chest X-ray is used to visualise macroscopic lung metastases. There remains controversy as to whether iodinated contrast should be used if a CT scan is undertaken due to concerns that this may lead to a delay in radioiodine therapy (Perros *et al.* 2014). If contrast enhancement will help define the extent of disease and help management decision-making, it should be used. Evidence is not clear as to the washout time required following intravenous contrast to prevent ‘stunning’ and reduced uptake of radioiodine therapy, but adult guidelines suggest waiting 8 weeks (Perros *et al.* 2014).

57. **Consider the use of palliative targeted therapy in CYP with progressing radioiodine refractory DTC** *(Moderate Recommendation, Moderate-Quality Evidence)*

Radioiodine refractory disease includes either the presence of at least one lesion that does not take up I-131 or clinical evidence that I-131 is no longer providing benefit. There is no evidence that traditional chemotherapeutic agents are an effective treatment of radioiodine refractory DTC in CYP. Targeted agents, sorafenib and lenvatinib, have been licensed more generally for the treatment of radioiodine refractory disease in adults but have not been proven in the paediatric population. In young adults over the age of 16 with progressing (i.e., with radiographic evidence of disease progression), radioiodine refractory DTC, sorafenib and lenvatinib can be considered as per marketing approval and based on phase III data from DECISION (Brose *et al.* 2014) and SELECT (Schlumberger *et al.* 2015) trials, respectively. These drugs should be administered under the supervision of clinicians with experience in managing these drugs and associated toxicities (Brose *et al.* 2012).

The use of next-generation sequencing to identify gene alterations, including BRAF mutations, RET, ALK and NTRK gene fusions, depends on the availability of such testing and NHS England is currently establishing a national test directory service over seven genomic hubs UK-wide to carry out cancer genomic testing by next-generation sequencing and interpret all results. Currently, the service offers testing in paediatric DTC via a multi-target next-generation sequencing panel for RET small and structural variants and NTRK1/2/3 structural variants (https://www.england.nhs.uk/publication/national-genomic-test-directories/). Future studies in CYP will likely help to direct targeted therapies for the treatment of individuals with particular somatic point mutations and fusion genes (Nies *et al.* 2021).

NICE has recommended the use of Larotrectinib (https://www.nice.org.uk/guidance/ta630) within the Cancer Drugs Fund as an option for treating NTRK fusion-positive solid tumours in adults and children if the disease is locally advanced or metastatic, or surgery could cause severe health problems and they have no satisfactory treatment options. Entrectinib has been recommended for use under similar circumstances in children over 12 years of age if they have not had treatment with an NTRK inhibitor previously (https://www.nice.org.uk/guidance/ta644). Clinical trials of RET inhibitors are ongoing.

In the rare situation where CYP are not cured of their DTC, palliative care teams should be involved in care at an early stage. Symptom control may include palliative radiotherapy, in a similar manner to as described above in Recommendation 55. Other locally ablative treatment modalities such as surgery, radiofrequency ablation and vertebroplasty can be considered to treat deposits of disease that are causing specific symptoms.

58. **Consider the use of external beam radiotherapy for symptom control in the palliative setting** *(Moderate Recommendation, Low-Quality Evidence, Delphi Consensus 73%)*

External beam radiotherapy is very rarely indicated in CYP with DTC in the primary or adjuvant setting because their disease is usually very iodine avid and sensitive so there is no benefit from the addition of external beam radiotherapy (Hay *et al.* 2010).

External beam radiotherapy to the neck can be of use in the palliative setting for symptom control, for example in cases of unresectable disease invading the larynx, trachea or oesophagus, where uncontrolled growth of the disease will cause life-threatening or distressing symptoms. There may also be a role in palliating the effects of more distant metastases for example painful bone metastases, bleeding or obstructing deposits of tumour or brain metastases. Any external beam radiotherapy administered should be delivered in a dedicated paediatric radiotherapy centre (Landau *et al.* 2000).

## Discussion

The rarity of paediatric endocrine tumours like DTC makes their management challenging. During the process of guideline development, we have confirmed a general lack of high-quality evidence relating to this age group and identified, through the consensus surveys necessarily undertaken, a professional mandate for both national speciality advisory panels and most importantly, a national register and evaluation of key management outcomes in these rare, eminently curable young patients. If we are to enhance clinical trials and quality of evidence, improve the health-related quality of survival and improve access to, and equity of, expertise in care, such a national register and centralised, advisory panel needs to be expedited alongside the development of tertiary, dedicated and age-appropriate, endocrine oncology multidisciplinary teams and services.

The GDG believes that in the UK no child should be looked after without a full thyroid cancer MDT. If no CYP-specific MDT is available, then these patients should be discussed at an adult thyroid cancer MDT. Working through and in collaboration with existing MDTs for adult and paediatric thyroid cancer will ensure the best use of resources in the current financial climate. We also recommend treatment at a high-volume tertiary centre where technologies and expertise can be most easily accessed, and the process streamlined with regards to ease of decision making and patient flow. Some additional funding may be needed, for the attendance of additional personnel at the existing MDTs and an ongoing programme of evaluation and audit, but any cost implications for health care budgets must be considered alongside the likely benefits inherent in concentrating management of rare conditions in specific expert MDTs. These include improvements in clinical outcomes and reduced complication rates both of which also greatly improve the patient experience. Financial savings are also produced by more efficient referral pathways, reduction in unnecessary and inappropriate investigations and avoiding unnecessary surgical interventions. As the aim of these guidelines is to improve patient care and experience, long-term complications should be reduced with potentially associated improved costs.

## Supplementary Material

Supplementary Material

## Declaration of interest

Dr Helen Spoudeas: founder of SUCCESS Charity – Life After Cure, www.successcharity.org advocating for the unmet health needs of patients surviving childhood brain tumours registered as LC in January 2019; founder of the national HPAT Virtual interest forum, piloting multidisciplinary virtual decision-making for children with complex hypothalamo-pituitary tumours. The other authors have no conflicts of interest to declare.

## Funding

Dr M N Gaze is supported by the National Institute for Health Research, University College London Hospitals Biomedical Research Centre, London, UK and by the Radiation Research Unit at the Cancer Research UKK City of London Centre Award (C7893/A28990). The guideline development was sponsored by unrestricted grants from Sandoz Pharmaceuticals, the professional societies CCLG, BSPED and The Society of British Neurological Surgeons, and the patient support groups ‘Association of Multiple Endocrine Neoplastic Disorders’ (AMEND), ‘SUCCESS Charity – Life After Cure’ and ‘The Pituitary Foundation’. Excepting as stakeholders, the sponsors had no role in development of guideline methodology or final guideline recommendations. The CCLG provided administrative support throughout the guideline and the RCPCH provided advice and appraised the guideline at different stages.

## Endorsing organisations

RCPCH.

## Author contribution statement

T R Kurzawinski and M N Gaze contributed equally to this work. HAS chaired the project board, obtained the funding and co-ordinated the GDG set up. SRH, TRK and MG led the GDG. SRH, TRK and MG were responsible for the study concept and design and drafted the manuscript. SRH and SF undertook the literature search. All authors took part in the grading process, interpreted the data, revised the manuscript critically for important intellectual content, approved the final version to be published, and agreed to be accountable for all aspects of the work.
