# Supplementary material for: Paediatric differentiated thyroid carcinoma: a UK National Clinical Practice Consensus Guideline
Source: Endocr Relat Cancer. 2022 Jun 23;29(11):G1–G33. doi: 10.1530/ERC-22-0035 (PMC9513650; doi:10.1530/ERC-22-0035)
Supplement: Supplementary Material [file supplementary_material.pdf]

Appendix 1 – GDG Members, Stakeholders and Delphi participants

A. DTC GDG members

Dr Sasha Howard	Senior Lecturer and Honorary Consultant in Paediatric Endocrinology, Barts and the London School of Medicine and Dentistry, QMUL and Barts Health NHS Trust, London (chair)	Chair<br><br>Conflicts of interest: None
Dr Sarah Freeston	Foundation Year 2 Doctor, Whipps Cross Hospital, Barts Health, London	Conflicts of interest: None
Mr Barney Harrison	Retired Consultant Endocrine Surgeon, Sheffield	Conflicts of interest: None
Dr Louise Izatt	Consultant Clinical and Cancer Geneticist, Guy's and St Thomas' NHS Foundation Trust, London	Conflicts of interest: None
Dr Sonali Natu	Clinical Director of Pathology and Consultant Histopathologist, University Hospital of North Tees and Hartlepool NHS Trust, Stockton-on-Tees	Conflicts of interest: None
Dr Kate Newbold	Consultant Clinical Oncologist, Royal Marsden Hospital Foundation Trust, London	Conflicts of interest: None
Dr Sabine Pomplun	Consultant Histo-/Cytopathologist, University College London Hospital NHS Foundation Trust, London	Conflicts of interest: None
Dr Helen Spoudeas	Consultant Paediatric Endocrinologist, Great Ormond Street Hospital for Children NHS Foundation Trust, London	Conflicts of interest: Founder of SUCCESS Charity Life After Cure; www.successcharity.org advocating for the unmet health needs of patients surviving childhood brain tumours registered as LC in January 2019<br><br>Founder of the national HPAT

		Virtual interest forum, piloting multidisciplinary virtual decision making for children with complex hypothalamo-pituitary tumours
Dr Sophie Wilne	Consultant Paediatric Oncologist, Nottingham University Hospital's NHS Trust, Nottingham	Conflicts of interest: None
Mr Tom Kurzawinski	Consultant Pancreatic and Endocrine Surgeon, University College London Hospital NHS Foundation Trust, University College London and Great Ormond Street Hospital for Children NHS Foundation Trust, London	Conflicts of interest: None
Dr Mark Gaze	Consultant Clinical Oncologist, University College London Hospital NHS Foundation Trust, Great Ormond Street Hospital for Children NHS Foundation Trust, London	Conflicts of interest: None

Conflicts of interest

All GDG and Delphi consensus group participants were asked to declare any conflicts of interests as per the NICE conflicts of interest policy. Conflicts were reviewed and no relevant conflicts identified. The guideline development was sponsored by unrestricted grants from Sandoz Pharmaceuticals, the patient support groups: Association of Multiple Endocrine Neoplasia (AMEND), SUCCESS Charity – Life After Cure, and The Pituitary Foundation, and the professional societies: The British Society of Neurosurgeons, the Children's Cancer & Leukaemia Group (CCLG) and the British Society for Paediatric Endocrinology & Diabetes (BSPED). Excepting as stakeholders (excluding Sandoz), the sponsors had no role in development of guideline methodology or final guideline recommendations. The CCLG provided administrative support throughout the guideline and the RCPCH provided advice and appraised the guideline at different stages.

Editorial independence: All GDG members declared any conflicts of interests prior to the guideline development starting, and periodically throughout the development of the guideline. The role of the funding bodies declared by Dr Helen Spoudeas did not influence the content of the guideline beyond that of the stakeholder process described in the main text.

External peer reviewers

Dr Tim Cheetham (paediatric endocrinologist), Newcastle upon Tyne Hospitals NHS Foundation Trust, Newcastle

Dr Laura Moss (thyroid oncologist), Velindre Cancer Centre, Cardiff

Mr Tarek Abdel-Aziz (thyroid surgeon), University College London Hospital NHS Foundation Trust, London

Mr Colin Butler (thyroid surgeon), University College London Hospital NHS Foundation Trust, London

Dates of consultation:

External review: Dec 2020 -March 2021

Stakeholder consultation: July 2021 – September 2021

B. DTC Guideline Stakeholders

Children's Cancer and Leukaemia Group (CCLG)
NCRI Thyroid Cancer Subgroup and Thyroid Cancer Forum –UK (TCF-UK)
Royal College of Paediatrics & Child Health (RCPCH)
Royal College of Physicians Clin Committee (including the RCP Young Adult and Adolescent Steering Group and Joint Specialty Committee (JSC) for Endocrinology and Diabetes Mellitus)
UK Endocrine Pathology Service (UK-EPS)

C. Delphi Consensus Process Participants

Name	Speciality	Institution	Country
Tabitha Randell	Consultant Paediatric Endocrinologist	Nottingham Children's Hospital	UK
Sarah Johnson	Consultant Pathologist	Newcastle upon Tyne NHS Foundation Trust	UK
Fiona Eatock	Consultant Endocrine Surgeon	Belfast Health and Social Care Trust	Northern Ireland, UK
Nicholas Reed	Consultant Clinical Oncologist	Beaston, Glasgow	UK
Rossella Elisei	Associate Professor of Endocrinology	University of Pisa, Endocrine Unit.	Italy
Radu Mihai	Consultant Endocrine Surgeon	Oxford university Hospitals NHS Trust	UK
Steven Waguespack	MD	UT MD Anderson Cancer Centre	USA
Clive Harmer	Retired Consultant	The Royal Marsden Hospital	UK
Ulla Feldt-Rasmussen	Professor, Senior Consultant	Rigshospitalet, Copenhagen University	Denmark
Saba Balasubramania n	Consultant Endocrine Surgeon	Sheffield Teaching Hospitals NHS Foundation Trust	UK
Jon Wadsley	Consultant	Weston park hospital, Sheffield	UK
Patrick Morrison	Geneticist	Belfast City Hospital	UK
Johannes Smit	Endocrinolgist	Radbound University Medical Centre	Netherlands
Gary Francis	Paediatric Endocrinologist	VCU	US
Denis Remidios	Consultant Radiologist	Northwick Park Hospital	UK
Timothy Stephenson	Histopathologist	Royal College of Pathologists	UK
Elizabeth Crowne	Paediatric Endocrinologist	University Hospitals Bristol Foundation Trust	UK
Victoria Murday	Consultant Geneticist	Retired	UK

D. Scope

This guideline is for all healthcare professionals caring for children and young people up to the age of 19 years with suspected or confirmed differentiated thyroid carcinoma (DTC). This includes health professionals from a variety of disciplines (paediatric endocrinology, oncology, surgery, radiology, histopathology, cytology and genetics) involved in the management and long-term follow-up of childhood and adolescent differentiated thyroid carcinomas.

Evidenced based or consensus recommendations have been made on the following:

- Presentation of DTC
- Genetics of DTC
- Cytology of DTC
- Histology of DTC
- Imaging of DTC
- Surgery for DTC
- Radio-iodine remnant ablation and treatment for DTC
- Follow up of DTC

Relevant parent and professional organisations / societies and charities (see page 2) were identified by the GDG at outset. The GDG Scope was sent to lead individuals and was revised where appropriate.

Target population: This guideline covers the management of all CYP diagnosed before their 19th birthday with suspected or confirmed differentiated thyroid carcinoma (DTC). It does not cover the management of patients over 19 years of age, nor does it cover patients with medullary thyroid carcinoma.

Appendix 2: Clinical Questions

Presentation

Subheading	Section	Clinical Question	Clinical Search Strategy
Demographics	Age Groups	Demographic data of age of presentation of DTC	P: Patients 0-18yrs with DTC<br>I:<br>C:<br>O: Spectrum/ peak age of first presentation with DTC
	Gender/ Ethnicity	Prevalence in males vs females, different ethnic groups	P: Patients 0-18yrs with DTC<br>I:<br>C:<br>O: Prevalence females vs males, different ethnicity
	Disease sub-type	Prevalence of papillary, follicular and rare aggressive histological sub-types of DTC	P: Patients 0-18yrs with DTC<br>I:<br>C:<br>O: Prevalence of sub-types of DTC in these patients
Signs & Symptoms	Thyroid swelling	Solitary nodule	P: Patients 0-18yrs with DTC<br>I<br>C<br>O: Demographics and specifics of presenting signs/symptoms
		Dominant nodule	
		Diffuse or simple multinodular Goitre	
	Cervical Lymphadenopathy		
	Lung metastases		
Incidental		Is incidental pick-up of thyroid nodule on scanning relevant to Paediatric population?	

**Screening**	**Assessment following irradiation**	Whom should we be screening?<br>Main conditions at risk e.g. ALL, Hodgkin's lymphoma, length of treatment, time since treatment, radiation exposure threshold	P: Patients 0-18yrs with DTC post-irradiation<br>I<br>C<br>O: Demographics of patients in this sub-group
	**Familial thyroid cancer screening**	How frequently should we be screening and with what Ix?	P: Patients 0-18yrs with DTC (familial thyroid cancer)<br>I<br>C<br>O: Demographics of patients in this sub-group

Diagnosis

Subheading	Section	Clinical Question	Clinical Search Strategy
**Biochemistry**	**TFT**	Do abnormal TFTs affect prognosis and therefore management in children with thyroid nodule	P: Patients 0-18yrs presenting with possible DTC
	**Serum Calcium**	Is this only indicated pre-operatively?	P: Patients 0-18yrs presenting with possible DTC
	**Thyroid antibodies**	? Should be part of diagnostic work-up<br>?Does being auto-antibody positive influence future management	P: Patients 0-18yrs presenting with possible DTC
**Imaging**	**US**	Reliability (Sensitivity + Specificity)<br>Does US help avoid multiple operations?<br>Does US help identify best nodule for biopsy?	**P: Patients 0-18yrs presenting with possible DTC**<br>**I:US pre-operative**<br>**C: No US/CT/MRI**<br>**O: Correct diagnosis, reduce operations, optimise other Ix**
	**CT-scan**	Reliability (Sensitivity + Specificity) – are these ever indicated?	P: Patients 0-18yrs presenting with possible DTC<br>I:CT/MRI

	**MRI**	Reliability (Sensitivity + Specificity)	C: No CT/MRI<br>O: Correct diagnosis, reduce operations, optimise other Ix
**Cytology**	**Fine needle aspiration**	Value of FNA for diagnosis of DTC in children<br>Age cut off for tolerating FNA	P: Patients 0-18yrs with isolated/dominant thyroid nodule<br>I: Fine needle aspiration<br>C: Proceed directly to surgery<br>O: Misdiagnosis? Multiple operations? Vs side effects of FNA

Management

Subheading	Section	Clinical Question	Clinical Search Strategy
**Surgical (pre-op)**	**Staging**	Cytology for pre-op staging<br>Best staging system?<br>Impact on prognosis?	P: Patients 0-18yrs with DTC<br>I: TMN<br>C: Other cytology-based staging systems<br>O: Prognostic accuracy of staging
		Histology for staging – papillary, follicular<br>Best staging system?<br>Impact on prognosis?	P: Patients 0-18yrs with DTC<br>I: TMN<br>C: Other histology-based staging systems<br>O: Prognostic accuracy of staging
	**Vocal cord assessment**		
**Surgical**	**Small tumour surgery**	Extent of surgery for small tumours (particularly microcarcinomas)	P: Patients 0-18yrs with small DTC<br>I: Total Lobectomy & Isthmectomy +/- extension<br>C: Hemithyroidectomy +/- total<br>O: Recurrence rate, RLN inury and other side effects

	**Surgery for <10yrs patients**	Advised extent of surgery for under 10 yr old patients	P: Patients 0-10yrs with DTC<br>I: Total thyroidectomy + RI<br>C: Less invasive surgery/ no RI<br>O: Recurrence rates
	**Lymph node removal**	Extent of lymph node removal to be recommended	P: Patients 0-18yrs with small DTC<br>I: Different lymph node resection approaches<br>C:<br>O: Recurrence rate
	**Complications from surgery**	RLN injury incidence<br>Post-op requirement for Ca treatment	P: Post-operative patients 0-18yrs with DTC<br>I<br>C<br>O: Prevalence of specific complications post surgery
**Post-operative**	**Radioiodine to ablate remnant tissue**	Pre-adolescents – optimal dose, inclusion/exclusion criteria, considerations eg T3 discontinuation, contrast media use,	P: Post-operative patients 0-18yrs with DTC<br>I: Differing regimes of radioiodine<br>C:<br>O: Recurrence rates and side effects
		Adolescents with tumours >1cm – optimal dose, inclusion/exclusion criteria, considerations eg sperm cryopreservation	
		Optimal time from surgery to radioiodine treatment	
**Long-term medication**	**Suppression with thyroxine**	Timecourse, dose, under who's management, TSH level to be maintained	P: Post-operative patients 0-18yrs with DTC<br>I: Differing regimes of levothyroxine suppression<br>C:<br>O: Recurrence rates

	**Human recombinant thyrotropin (TSH)**	Efficacy and safety of recombinant TSH for thyroid remnant ablation with RI in children	P: Post-operative patients 0-18yrs with DTC receiving RI<br>I: rhTSH<br>C: No rhTSH<br>O: Cure and recurrence rates and side effects

Appendix 3: Literature Search

Searches were conducted using the Ovid MEDLINE database (1990 – March 2015), the Cochrane Library, TRIP and the EMBASE database using a combination of keywords, and MeSH subject headings relating to the epidemiology, screening, diagnosis, investigation, genetic counselling, management, follow-up and prognosis of patients with differentiated thyroid cancer up to the age of 19 years. A further search was added for papers published March 2015 – August 2020 using the same databases and methodology to ensure the most up to date literature was included (see figure 2, page 26). Letters, editorials and unpublished studies were excluded.

Overall, 230 published primary studies were identified in this way (177 from the initial search in March 2015, and 53 from the search up to August 2020). The literature searches also uncovered 3 national/ international published evidence-based guidelines which are discussed and referenced within this text. An additional 26 papers were included following the peer-review process prior to publication.

1. exp Thyroid neoplasms/
2. (thyroid neoplasm* or differentiated thyroid carcinoma or DTC or thyroid nodule* or papillary carcinoma or follicular carcinoma or (thyroid adj3 cancer) or thyroid carcinoma or (thyroid adj2 tumo?r*) or thyroid metastas* or thyroid adenoma).mp. [mp=title, abstract, original title, name of substance word, subject heading word, keyword heading word, protocol supplementary concept word, rare disease supplementary concept word, unique identifier]
3. 1 or 2
4. limit 3 to (english language and yr="1990 -Current" and "all child (0 to 18 years)")
   Is the incidence of DTC in children on the rise?
5. exp Incidence/
6. incidence.mp. [mp=title, abstract, original title, name of substance word, subject heading word, keyword heading word, protocol supplementary concept word, rare disease supplementary concept word, unique identifier]
7. 5 or 6
8. 4 and 7
9. limit 8 to (english language and yr="1990 -Current" and "all child (0 to 18 years)")
   What is an incidence & prevalence of DTC in males vs females?
10. exp Prevalence/
11. prevalence.mp. [mp=title, abstract, original title, name of substance word, subject heading word, keyword heading word, protocol supplementary concept word, rare disease supplementary concept word, unique identifier]
12. 10 or 11
13. 4 and 12
14. limit 13 to (english language and male and female and yr="1990 -Current" and "all child (0 to 18 years)")
15. (male* or female* or gender or girl* or boy*).mp. [mp=title, abstract, original title, name of substance word, subject heading word, keyword heading word, protocol supplementary concept word, rare disease supplementary concept word, unique identifier]
16. 13 and 15
    What is the incidence and prevalence of DTC in different ethnic groups?
17. exp Population Groups/

18. (ethnic* or minorit* or rac*).mp. [mp=title, abstract, original title, name of substance word, subject heading word, keyword heading word, protocol supplementary concept word, rare disease supplementary concept word, unique identifier]
19. 17 or 18
20. 4 and 12 and 19

    What is the incidence of thyroid nodules in children?
21. exp Thyroid Nodule/
22. thyroid nodule*.mp. [mp=title, abstract, original title, name of substance word, subject heading word, keyword heading word, protocol supplementary concept word, rare disease supplementary concept word, unique identifier]
23. 21 or 22
24. limit 23 to (english language and yr="1990 -Current" and "all child (0 to 18 years)")
25. 12 and 24

    What is the incidence of thyroid cancer in thyroid nodules found in children?
26. 4 and 7 and 23

    What is the prevalence of papillary and follicular DTC?
27. (papillary or follicular).mp. [mp=title, abstract, original title, name of substance word, subject heading word, keyword heading word, protocol supplementary concept word, rare disease supplementary concept word, unique identifier]
28. limit 27 to (english language and yr="1990 -Current" and "all child (0 to 18 years)")
29. 12 and 28

    What is the age of presentation of DTC? Average and range.
    What is the most common presentation of DTC in children?
30. exp Age Distribution/
31. ((age adj2 presentation) or demographic*).mp. [mp=title, abstract, original title, name of substance word, subject heading word, keyword heading word, protocol supplementary concept word, rare disease supplementary concept word, unique identifier]
32. 30 or 31
33. 4 and 32
34. limit 33 to (english language and yr="1990 -Current" and "all child (0 to 18 years)")
35. (presentation or symptom* or sign*).mp. [mp=title, abstract, original title, name of substance word, subject heading word, keyword heading word, protocol supplementary concept word, rare disease supplementary concept word, unique identifier]
36. limit 35 to (english language and yr="1990 -Current" and "all child (0 to 18 years)")
37. 4 and 36

    Solitary or dominant nodule in multinodular goitre?
38. ((solitary or dominant) and nodule).mp. [mp=title, abstract, original title, name of substance word, subject heading word, keyword heading word, protocol supplementary concept word, rare disease supplementary concept word, unique identifier]
39. limit 38 to (english language and yr="1990 -Current" and "all child (0 to 18 years)")
40. 4 and 39

    Diffuse or simple multinodular goitre?
41. thyroid swelling.mp. [mp=title, abstract, original title, name of substance word, subject heading word, keyword heading word, protocol supplementary concept word, rare disease supplementary concept word, unique identifier]
42. limit 41 to (english language and yr="1990 -Current" and "all child (0 to 18 years)")
43. 4 and 42 – thyroid swelling
44. multinodular goitre.mp. [mp=title, abstract, original title, name of substance word, subject heading word, keyword heading word, protocol supplementary concept word, rare disease supplementary concept word, unique identifier]
45. limit 44 to (english language and yr="1990 -Current" and "all child (0 to 18 years)")

46. 4 and 45
    What is the incidence of thyroid cancer in thyroglossal cyst in children?
47. thyroglossal cyst.mp. [mp=title, abstract, original title, name of substance word, subject heading word, keyword heading word, protocol supplementary concept word, rare disease supplementary concept word, unique identifier]
48. limit 47 to (english language and yr="1990 -Current" and "all child (0 to 18 years)")
49. 4 and 48
    Is cervical lymphadenopathy a common presentation of DTC in children?
50. cervical lymphadenopathy.mp.
51. 4 and 50
    Lung metastases
52. lung metastas*.mp. [mp=title, abstract, original title, name of substance word, subject heading word, keyword heading word, protocol supplementary concept word, rare disease supplementary concept word, unique identifier]
53. exp Lung Neoplasms/
54. 52 or 53
55. 4 and 54
    Is incidental pick-up of thyroid nodule on scanning relevant to the paediatric population?
56. ((diagnos* or sign) and (scan* or imag* or incident*)).mp. [mp=title, abstract, original title, name of substance word, subject heading word, keyword heading word, protocol supplementary concept word, rare disease supplementary concept word, unique identifier]
57. limit 56 to (english language and yr="1990 -Current" and "all child (0 to 18 years)")
58. 4 and 57
59. incidental findings/
60. 4 and 59
61. 57 or 59
62. 4 and 61
    Whom should we be screening?
    What is the risk from radiation or chemotherapy?
63. exp Radiation Effects/
64. *radiation/ or chemotherapy.mp. or radiotherapy.mp. [mp=title, abstract, original title, name of substance word, subject heading word, keyword heading word, protocol supplementary concept word, rare disease supplementary concept word, unique identifier]
65. limit 64 to (english language and yr="1990 -Current" and "all child (0 to 18 years)")
66. 63 or 65
67. (screen* or assess*).mp. [mp=title, abstract, original title, name of substance word, subject heading word, keyword heading word, protocol supplementary concept word, rare disease supplementary concept word, unique identifier]
68. 66 and 67
69. 4 and 68
    Which are the main conditions at risk e.g., ALL, Hodgkin's lymphoma (assessment following irradiation)?
70. exp Leukemia, Lymphoid/ or exp Precursor Cell Lymphoblastic Leukemia-Lymphoma/
71. Acute lymphoblastic leukaemia.mp. [mp=title, abstract, original title, name of substance word, subject heading word, keyword heading word, protocol supplementary concept word, rare disease supplementary concept word, unique identifier]
72. 70 or 71
73. risk/
74. 72 and 73
75. 4 and 74
76. 4 and 73

77. 4 and 72
78. Hodgkin Disease/
79. Hodgkin's lymphoma.mp. [mp=title, abstract, original title, name of substance word, subject heading word, keyword heading word, protocol supplementary concept word, rare disease supplementary concept word, unique identifier]
80. 78 or 79
81. 4 and 80
82. exp Disease Susceptibility/ci, de, dt, re [Chemically Induced, Drug Effects, Drug Therapy, Radiation Effects]
83. 4 and 82

    Familial thyroid cancer screening – which are the familial syndromes requiring screening for DTC?
    How frequently should we be screening and with what investigations?
    What constitutes high risk for development of thyroid cancer in children?
84. exp Genetic Predisposition to Disease/
85. 4 and 84
86. (familial or genetic or family).mp. [mp=title, abstract, original title, name of substance word, subject heading word, keyword heading word, protocol supplementary concept word, rare disease supplementary concept word, unique identifier]
87. 84 or 86
88. 67 and 87
89. 4 and 88
90. 4 and 73

    Transplantation and immunosuppression (solid organs, bone marrow)
    What is the time lag between treatment and development of DTC?
91. (transplant* or immunosuppress*).mp. [mp=title, abstract, original title, name of substance word, subject heading word, keyword heading word, protocol supplementary concept word, rare disease supplementary concept word, unique identifier]
92. exp Transplantation/
93. 91 or 92
94. 4 and 93

    Do normal TFTs affect prognosis and therefore management in children with thyroid nodule?
95. exp Thyroid Function Tests/
96. 4 and 95

    What blood tests should be performed preoperatively?
97. ((blood test or thyroglobulin or calcitonin or serum calcium or PTH) and (preop or preoperative* or presurg* or preop*)).mp. [mp=title, abstract, original title, name of substance word, subject heading word, keyword heading word, protocol supplementary concept word, rare disease supplementary concept word, unique identifier]
98. 4 and 97
99. Thyroglobulin/
100. (preop or preoperative* or presurg* or preop*).mp. [mp=title, abstract, original title, name of substance word, subject heading word, keyword heading word, protocol supplementary concept word, rare disease supplementary concept word, unique identifier]
101. limit 99 to (english language and yr="1990 -Current" and "all child (0 to 18 years)")
102. (blood test or thyroglobulin or calcitonin or serum calcium or PTH).mp. [mp=title, abstract, original title, name of substance word, subject heading word, keyword heading word, protocol supplementary concept word, rare disease supplementary concept word, unique identifier]
103. limit 102 to (english language and yr="1990 -Current" and "all child (0 to 18 years)")
104. 99 or 103
105. 101 or 104
106. 4 and 105

Should antibodies be part of thyroid workup?
Does being antibody positive influence future management?

107. ((diagnos* or workup or work-up) and antibod*).mp. [mp=title, abstract, original title, name of substance word, subject heading word, keyword heading word, protocol supplementary concept word, rare disease supplementary concept word, unique identifier]
108. limit 107 to (english language and yr="1990 -Current" and "all child (0 to 18 years)")
109. 4 and 108
110. autoantibod*.mp. [mp=title, abstract, original title, name of substance word, subject heading word, keyword heading word, protocol supplementary concept word, rare disease supplementary concept word, unique identifier]
111. limit 110 to (english language and yr="1990 -Current" and "all child (0 to 18 years)")
112. 4 and 111

What is a sensitivity and specificity of thyroid US in detecting thyroid nodules and differentiating benign and malignant pathology?
Does US help avoid multiple operations?
Does US help identify best nodule for biopsy?
What are typical sonographic features of thyroid cancer?
What are potential differential diagnoses on US?
Should US scoring systems ie KIm be used in children
How should an US be reported (benign, malignant, indeterminate?)

113. exp Ultrasonography/
114. ultrasound.mp. [mp=title, abstract, original title, name of substance word, subject heading word, keyword heading word, protocol supplementary concept word, rare disease supplementary concept word, unique identifier]
115. 113 or 114
116. 4 and 115

Should neck and chest CT scan be performed in all children assessed for thyroid cancer?
What is the sensitivity and specificity of CT scan?

117. exp Tomography, X-Ray Computed/
118. ((CT or computed tomography or neck) and chest CT).mp. [mp=title, abstract, original title, name of substance word, subject heading word, keyword heading word, protocol supplementary concept word, rare disease supplementary concept word, unique identifier]
119. 117 or 118
120. 4 and 119

Should routine assessment of thyroid nodules include MRI and radioisotope scanning?

121. exp Magnetic Resonance Imaging/
122. (MRI or magnetic resonance imaging).mp. [mp=title, abstract, original title, name of substance word, subject heading word, keyword heading word, protocol supplementary concept word, rare disease supplementary concept word, unique identifier]
123. exp Radionuclide Imaging/
124. (radioisotope scan* or isotope scan*).mp. [mp=title, abstract, original title, name of substance word, subject heading word, keyword heading word, protocol supplementary concept word, rare disease supplementary concept word, unique identifier]
125. 121 or 122 or 123 or 124
126. 4 and 125

Is FNAC more informative when guided by US in comparison with 'free hand' biopsy?

127. exp Biopsy, Fine-Needle/

128. fine needle aspiration.mp. [mp=title, abstract, original title, name of substance word, subject heading word, keyword heading word, protocol supplementary concept word, rare disease supplementary concept word, unique identifier]

129. 127 or 128

130. 4 and 129

131. exp Endoscopic Ultrasound-Guided Fine Needle Aspiration/

132. 4 and 131

What is the age cut-off for tolerating FNA under local anaesthesia?

133. 4 and 32 and 129

What is the role of preoperative FNA for diagnosis of DTC in children?

134. diagnos*.mp. [mp=title, abstract, original title, name of substance word, subject heading word, keyword heading word, protocol supplementary concept word, rare disease supplementary concept word, unique identifier]

135. 4 and 100 and 129 and 134

Is there an advantage of core biopsy over cytology?

136. (biops* or cytolog*).mp. [mp=title, abstract, original title, name of substance word, subject heading word, keyword heading word, protocol supplementary concept word, rare disease supplementary concept word, unique identifier]

137. 4 and 136

Should molecular resting be used for diagnosing malignancy on cytology samples?
Should molecular testing be used for diagnosing malignancy on histology samples?

138. molecular test*.mp. [mp=title, abstract, original title, name of substance word, subject heading word, keyword heading word, protocol supplementary concept word, rare disease supplementary concept word, unique identifier]

139. 4 and 134 and 136 and 138

Should Thy 1-5 classification be used in children?

140. exp Classification/

141. classification*.mp. [mp=title, abstract, original title, name of substance word, subject heading word, keyword heading word, protocol supplementary concept word, rare disease supplementary concept word, unique identifier]

142. 140 or 141

143. 4 and 142

When to refer children diagnosed with a thyroid nodule?
Where should children be referred for assessment?
What are the signs and symptoms that warrant urgent referral?
What is the timescale to referral / decision to treat and treatment for children with DTC?
Should all children with potential diagnosis of thyroid cancer be referred/discussed at regional MDT?

144. refer*.mp. [mp=title, abstract, original title, name of substance word, subject heading word, keyword heading word, protocol supplementary concept word, rare disease supplementary concept word, unique identifier]

145. 4 and 144
146. 4 and 36 and 144

Does the pathology reporting need to be done by a specialist pathologist/ someone with the experience in the field?
What should be the core data items included in the pathology report?

147. patholo* report*.mp. [mp=title, abstract, original title, name of substance word, subject heading word, keyword heading word, protocol supplementary concept word, rare disease supplementary concept word, unique identifier]
148. 4 and 147

What are criteria for diagnosing malignant follicular and papillary neoplasms?
Which immunocytochemistry markers (panel of markers) should be used to diagnose thyroid cancer? (BRAC, PTEN, p53, RET ??)
Should TMN system be used for tumour staging?

149. (stage or staging or TMN).mp. [mp=title, abstract, original title, name of substance word, subject heading word, keyword heading word, protocol supplementary concept word, rare disease supplementary concept word, unique identifier]
150. exp Neoplasm Staging/
151. 149 or 150
152. 4 and 151
153. exp Tumor Markers, Biological/
154. (marker or BRAC or PTEN or p53 or RET).mp. [mp=title, abstract, original title, name of substance word, subject heading word, keyword heading word, protocol supplementary concept word, rare disease supplementary concept word, unique identifier]
155. 153 or 154
156. 4 and 134 and 155
157. 4 and 134

What investigations should be completed before surgery in children with small and large tumours?
& Should all children have US before thyroid surgery?
& Should all children have cytological diagnosis before surgery?

1. exp Thyroid neoplasms/
2. (thyroid neoplasm* or differentiated thyroid carcinoma or DTC or thyroid nodule* or papillary carcinoma or follicular carcinoma or (thyroid adj3 cancer) or thyroid carcinoma or (thyroid adj2 tumo?r*) or thyroid metastas* or thyroid adenoma).mp. [mp=title, abstract, original title, name of substance word, subject heading word, keyword heading word, protocol supplementary concept word, rare disease supplementary concept word, unique identifier]
3. 1 or 2
4. limit 3 to (english language and yr="1990 -Current" and "all child (0 to 18 years)")

5. presurg*.mp. [mp=title, abstract, original title, name of substance word, subject heading word, keyword heading word, protocol supplementary concept word, rare disease supplementary concept word, unique identifier]
6. preop*.mp. [mp=title, abstract, original title, name of substance word, subject heading word, keyword heading word, protocol supplementary concept word, rare disease supplementary concept word, unique identifier]
7. 5 or 6

8. investigation.mp. [mp=title, abstract, original title, name of substance word, subject heading word, keyword heading word, protocol supplementary concept word, rare disease supplementary concept word, unique identifier]
9. assessment.mp. [mp=title, abstract, original title, name of substance word, subject heading word, keyword heading word, protocol supplementary concept word, rare disease supplementary concept word, unique identifier]
10. 8 or 9
11. 7 and 10
12. 4 and 11
13. Cytodiagnosis/ or Neoplasms/
14. cyto* diagnosis.mp. [mp=title, abstract, original title, name of substance word, subject heading word, keyword heading word, protocol supplementary concept word, rare disease supplementary concept word, unique identifier]
15. 13 or 14
16. 7 and 15
17. 8 and 16

What is the impact of preoperative staging on prognosis?

5. stage.mp. [mp=title, abstract, original title, name of substance word, subject heading word, keyword heading word, protocol supplementary concept word, rare disease supplementary concept word, unique identifier]
6. staging.mp. [mp=title, abstract, original title, name of substance word, subject heading word, keyword heading word, protocol supplementary concept word, rare disease supplementary concept word, unique identifier]
7. exp Neoplasm Staging/
8. exp Prognosis/
9. prognosis.mp. [mp=title, abstract, original title, name of substance word, subject heading word, keyword heading word, protocol supplementary concept word, rare disease supplementary concept word, unique identifier]
10. 8 or 9
11. 5 or 6 or 7
12. 4 and 10 and 11

Should all children have pre and post operative direct laryngoscopy to assess VC? & 22) What is the role of laryngoscopy?

5. laryngoscopy.mp. [mp=title, abstract, original title, name of substance word, subject heading word, keyword heading word, protocol supplementary concept word, rare disease supplementary concept word, unique identifier]
6. exp Laryngoscopy/
7. 5 or 6
8. 4 and 7

What is the role of US in assessing VC mobility in younger children?

5. ultrasound.mp.
6. vocal cord.mp. or exp Vocal Cords/
7. exp Laryngoscopy/ or exp Vocal Cord Paralysis/ or laryn*.mp.
8. 6 or 7
9. 5 and 8
10. 4 and 9

Who should perform surgery in cases of advanced/locally invasive thyroid cancer in children?

18. invasive.mp.
19. exp Thyroid Neoplasms/ or exp Thyroidectomy/ or Thyroid Nodule/ or thyroid surgery.mp.
20. 4 and 18 and 19

Where should such surgery be performed?

5. exp Diagnosis/ or exp Diagnosis, Differential/ or diagnos*.mp.
6. surgery.mp.
7. surgical.mp.
8. 6 or 7
9. location.mp. or exp Location
10. 8 and 9
11. 4 and 10

Which complications should be discussed (bleeding, infection, RLN injury, hypoparathyroidism)?
& What is the incidence of complications of thyroid surgery in children?

5. surgery.mp.
6. surgical.mp.
7. 5 or 6
8. complication*.mp. or exp Complication
9. 7 and 8
10. incidence.mp
11. 4 and 10

What information should be described in the operating notes?

5. exp Diagnosis/ or exp Diagnosis, Differential/ or diagnos*.mp.
6. surgery.mp.
7. surgical.mp.
8. 6 or 7
9. documentation.mp. or exp Documentation/
10. 8 and 9
11. 4 and 10

What constitutes diagnostic surgery?
& What are the criteria for hemi or total thyroidectomy?

5. exp Diagnosis/ or exp Diagnosis, Differential/ or diagnos*.mp.
6. surgery.mp.
7. surgical.mp.
8. 6 or 7
9. 5 and 8
10. 4 and 9
11. exp Adenocarcinoma, Follicular/ or exp Thyroidectomy/ or exp Thyroid Neoplasms/ or total thyroidectomy.mp. or exp Carcinoma, Papillary/
12. exp Thyroidectomy/ or exp Thyroid Nodule/ or exp Thyroid Neoplasms/ or hemi thyroidectomy.mp. or exp Adenocarcinoma, Follicular/ or exp Carcinoma, Papillary/
13. 11 or 12
14. 4 and 13

What is the impact of tumour type, size, multifocality, patients age on the extent of surgery?

5. exp Diagnosis/ or exp Diagnosis, Differential/ or diagnos*.mp.
6. surgery.mp.
7. surgical.mp.
8. 6 or 7
9. 5 and 8
10. 4 and 9
11. exp Adenocarcinoma, Follicular/ or exp Thyroidectomy/ or exp Thyroid Neoplasms/ or total thyroidectomy.mp. or exp Carcinoma, Papillary/
12. exp Thyroidectomy/ or exp Thyroid Nodule/ or exp Thyroid Neoplasms/ or hemi thyroidectomy.mp. or exp Adenocarcinoma, Follicular/ or exp Carcinoma, Papillary/
13. 11 or 12
14. 4 and 13

15. exp Age Factors/ or age.mp.
16. tumor size.mp.
17. 15 or 16
18. 8 and 17
19. 4 and 1
What is the recommended extent of surgery in children with familial or high risk (postradiation) DTC?
5. familial.mp.
6. high risk.mp.
7. 5 or 6
8. surgery.mp.
9. surgical.mp.
10. 8 or 9
11. 4 and 7 and 10
What is the extent of lymph node removal to be recommended? & 8) Who should perform lymph node dissection in children?
5. lymph node dissection.mp. or exp Lymph Node Excision/
6. Lymph Nodes/ or lymph node surg*.mp.
7. 5 or 6
8. 4 and 7
Should prophylactic lymphadenectomy be performed?
& What is the role of prophylactic and therapeutic LND?
5. exp Lymph Node Excision/
6. prophylactic lymphadenectomy.mp. [mp=title, abstract, original title, name of substance word, subject heading word, keyword heading word, protocol supplementary concept word, rare disease supplementary concept word, unique identifier]
7. 5 or 6
8. 4 and 7
How should short term hypocalcaemia be treated?
5. hypocalcaem*.mp. [mp=title, abstract, original title, name of substance word, subject heading word, keyword heading word, protocol supplementary concept word, rare disease supplementary concept word, unique identifier]
6. treat*.mp.
7. management.mp.
8. 6 or 7
9. 4 and 5 and 8
How should long term hypoparathyroidism be treated?
5. exp Hypoparathyroidism/ or exp Hyperparathyroidism/ or hypoparathyroid*.mp.
6. 4 and 6
What are the indications for initiating treatment with radioiodine?
5. exp Carcinoma, Papillary/ or exp Thyroid Neoplasms/ or exp Iodine Radioisotopes/ or radioiodine.mp.
6. exp Iodine Radioisotopes/ or exp Thyroid Neoplasms/ or exp Carcinoma, Papillary/ or I131.mp.
7. 5 or 6
8. dose.mp. or exp Dose Fractionation/ or exp Dose-Response Relationship, Radiation/
9. 7 and 8
10. 4 and 9
11. exp Disease-Free Survival/ or exp Survival/ or survival.mp.
12. recurrance.mp.
13. 11 or 12
14. 4 and 7 and 13

15. indication.mp.
16. 4 and 7 and 15
What is the recommended dose of I131 for ablation and subsequent treatment?
& How should the dose be calculated taking into account patient weight/BMI/age/ tumour staging/
& What is the optimal time from surgery to radioiodine treatment?
5. exp Carcinoma, Papillary/ or exp Thyroid Neoplasms/ or exp Iodine Radioisotopes/ or radioiodine.mp.
6. exp Iodine Radioisotopes/ or exp Thyroid Neoplasms/ or exp Carcinoma, Papillary/ or I131.mp.
7. 5 or 6
8. dose.mp. or exp Dose Fractionation/ or exp Dose-Response Relationship, Radiation/
9. 7 and 8
10. 4 and 9
Does treatment with I131 improve survival and reduce recurrence rate?
5. exp Carcinoma, Papillary/ or exp Thyroid Neoplasms/ or exp Iodine Radioisotopes/ or radioiodine.mp.
6. exp Iodine Radioisotopes/ or exp Thyroid Neoplasms/ or exp Carcinoma, Papillary/ or I131.mp.
7. 5 or 6
8. dose.mp. or exp Dose Fractionation/ or exp Dose-Response Relationship, Radiation/
9. 7 and 8
10. 4 and 9
11. exp Disease-Free Survival/ or exp Survival/ or survival.mp.
12. recurrence.mp.
13. 11 or 12
14. 4 and 7 and 13
What are the complications of I131 treatment?
5. exp Carcinoma, Papillary/ or exp Thyroid Neoplasms/ or exp Iodine Radioisotopes/ or radioiodine.mp.
6. exp Iodine Radioisotopes/ or exp Thyroid Neoplasms/ or exp Carcinoma, Papillary/ or I131.mp.
7. 5 or 6
8. 4 and 7
9. complication*.mp.
10. 8 and 9
Is rTSH administration better strategy than stopping thyroxine before administration of I131?
& Efficacy and safety of recombinant TSH for thyroid remnant ablation with RI in children
5. exp Thyroid Neoplasms/ or exp Thyrotropin/ or exp Iodine Radioisotopes/ or rTSH.mp. or exp Thyroid Gland/
6. thyrotropin.mp. or exp Thyrotropin/
7. 5 or 6
8. 4 and 7
9. exp Carcinoma, Papillary/ or exp Thyroid Neoplasms/ or thyroid remnant.mp. or exp Adenocarcinoma, Follicular/
10. 8 and 9
Should sperm cryopreservation be considered?
5. Cryopreservation/ or Sperm Motility/ or sperm cryopreservation.mp. or Semen Preservation/
6. exp Cryopreservation/ or exp Sperm Motility/ or sperm cryopreservation.mp. or exp Semen Preservation/
7. 5 and 6
8. 4 and 7
What are the indications for external beam radiotherapy to the neck?
What are the complications of EBR in children?
Does EBR prevent local recurrence and extend survival?

5. external beam radiotherapy.mp. or exp Radiotherapy Dosage/
6. 4 and 5
Which calcium supplementation should be used in children?
& Which vitamin D supplementation should be used in children?
5. postoperative care.mp. or exp postoperative care/
6. postop*mp.
7. 5 or 6
8. calcium supplement*.mp
9. vit* D supplement*.mp
10. 8 or 9
11. 7 and 10
How often should plasma calcium be measured?
How often should urine calcium be measured?
5. postoperative care.mp. or exp postoperative care/
6. postop*mp.
7. 5 or 6
8. measure*.mp. or test.mp. or exp test or assess*.mp.
9. plasma calcium.mp or urine calcium.mp.
10. 8 and 9
11. 7 and 10
12. 4 and 11
How often should renal US be performed?
5. postoperative care.mp. or exp postoperative care/
6. postop*mp.
7. 5 or 6
8. renal ultrasound.mp.
9. 7 and 8
10. 4 and 9
How often should bone scan be performed?
5. postoperative care.mp. or exp postoperative care/
6. postop*mp.
7. 5 or 6
8. bone scan.mp.
9. 7 and 8
10. 4 and 9
How soon after surgery and how often after I131 treatment should thyroglobulin be measured?
How should thyroglobulin levels during TSH suppression be interpreted?
What is the role and indication for ‘stimulated’ thyroglobulin measurement?
What levels of Thyroglobulin are suspicious of recurrence?
What are the cut off values for clinical decision making?
5. thyroglobulin.mp. or exp Thyroglobulin/
6. exp Recurrence/ or recur*.mp.
7. 5 and 6
8. 5 or 7
9. 4 and 8

Which children with genetic mutations are at high risk of developing DTC?
How common is DTC and its subtypes in high risk populations?
How common are mutations in children with DTC?
Which children should be referred for genetic counselling?
What is the impact on prognosis and management of: PTEN, RET, N-TRK, cMET?

How often should US be performed?
What are the indications for radioisotope or US scanning?
What is the best imaging modality to detect local recurrence?
What is the best imaging modality for detecting distal metastasis?
How often should thyroglobulin/TSH blood tests be performed?
How should Tg-positive, scan-negative patients be managed?
What should be the frequency and duration of follow-up for:

a) No genetic mutation; FHx positive
b) No genetic mutation; no Hx
c) Familial Adenomatosis Polyposis (FAP)
d) Cowden's Disease
e) Carney Complex
f) Exposure to ionising Radiation of the thyroid
g) Dominant papillary carcinoma families

What is the optimal age of transition and how to best arrange transition?
Is there a difference in long term prognosis between children and adults with DTC?
Recurrence rate in patients with DTC with and without known mutation
Rates and specifics of morbidity in patients with DTC with and without known mutations
Rates and specifics of mortality in patients with DTC with and without known mutations

1. exp Thyroid neoplasms/
2. (thyroid neoplasm* or differentiated thyroid carcinoma or DTC or thyroid nodule* or papillary carcinoma or follicular carcinoma or (thyroid adj3 cancer) or thyroid carcinoma or (thyroid adj2 tumo?r*) or thyroid metastas* or thyroid adenoma).mp. [mp=title, abstract, original title, name of substance word, subject heading word, keyword heading word, protocol supplementary concept word, rare disease supplementary concept word, unique identifier]
3. 1 or 2
4. limit 3 to (english language and yr="1990 -Current" and "all child (0 to 18 years)")

5. (((gene* or familial or family) adj2 history) or mutation).mp. [mp=title, abstract, original title, name of substance word, subject heading word, keyword heading word, protocol supplementary concept word, rare disease supplementary concept word, unique identifier]
6. 4 and 5
7. (incidence or prevalence or risk).mp. [mp=title, abstract, original title, name of substance word, subject heading word, keyword heading word, protocol supplementary concept word, rare disease supplementary concept word, unique identifier]
8. 4 and 7
9. 6 and 8
10. genetic counselling.mp. [mp=title, abstract, original title, name of substance word, subject heading word, keyword heading word, protocol supplementary concept word, rare disease supplementary concept word, unique identifier]
11. 4 and 10
12. Genetic Counseling/
13. 4 and 12
14. 11 or 13
15. (PTEN or RET or N-TRK or c-MET or "phosphatase and tension homolog" or cmet or NTRK or neurotrophin receptor tyrosine kinase).mp. [mp=title, abstract, original title, name of substance word, subject heading word, keyword heading word, protocol supplementary concept word, rare disease supplementary concept word, unique identifier]
16. 4 and 15
17. prognosis.mp. or Prognosis/

18. 16 and 17
19. postoperative care.mp. or Postoperative Care/
20. (postop* or postsurg* or "follow-up").mp. [mp=title, abstract, original title, name of substance word, subject heading word, keyword heading word, protocol supplementary concept word, rare disease supplementary concept word, unique identifier]
21. 19 or 20
22. exp Diagnostic Imaging/ or imaging.mp.
23. (ultrasound or ultrason*).mp. [mp=title, abstract, original title, name of substance word, subject heading word, keyword heading word, protocol supplementary concept word, rare disease supplementary concept word, unique identifier]
24. 22 or 23
25. 4 and 21 and 24
26. Neoplasm Recurrence, Local/ or neoplasm recurrence.mp.
27. 25 and 26
28. (thyroglobulin or thyroxine or t4 or t3 or tsh).mp. [mp=title, abstract, original title, name of substance word, subject heading word, keyword heading word, protocol supplementary concept word, rare disease supplementary concept word, unique identifier]
29. 4 and 26 and 28
30. prognosis.mp. or Prognosis/
31. recurrence risk.mp. [mp=title, abstract, original title, name of substance word, subject heading word, keyword heading word, protocol supplementary concept word, rare disease supplementary concept word, unique identifier]
32. 26 or 30 or 31
33. 4 and 32
34. neoplasms/ or adenomatous polyposis coli/
35. 33 and 34
36. cowden disease.mp. or Hamartoma Syndrome, Multiple/
37. 33 and 36
38. carney complex.mp. or Carney Complex/
39. 33 and 38
40. (ionising radiation adj3 thyroid).mp. [mp=title, abstract, original title, name of substance word, subject heading word, keyword heading word, protocol supplementary concept word, rare disease supplementary concept word, unique identifier]
41. ((ionising radiation or ionizing radiation) and thyroid).mp. [mp=title, abstract, original title, name of substance word, subject heading word, keyword heading word, protocol supplementary concept word, rare disease supplementary concept word, unique identifier]
42. 33 and 41
43. 4 and 21
44. exp Transition to Adult Care/
45. 4 and 44
46. morbidity.mp. or exp Morbidity/
47. exp Mortality/ or mortality.mp.
48. 46 or 47
49. 4 and 48
50. 16 and 49
51. 9 or 14 or 18 or 25 or 27 or 29 or 33 or 35 or 37 or 39 or 42 or 43 or 45 or 50
52. remove duplicates from 51

Criteria for selecting the evidence

The results of the literature search were screened and full-text versions titles fulfilling the inclusion criteria below were retrieved (see figure 2 below):

Inclusion criteria:
Population:
All children and adolescents (aged 0-18 years) with DTC related- pathology
Study design:
Case reports/ case series/ systematic reviews reporting on assessment/ management of patients with pathology secondary to DTC
Recommendations from consensus guidelines
Full text articles
Published in the English language

Exclusion criteria
Population:
Excluding Adults >18 years old
Excluding patients with medullary thyroid carcinoma
Study design:
Studies published prior to 1990
Personal practice, comments, letters and correspondence, published abstracts, book chapters, narrative reviews, and conference proceedings

Figure 2 – Systematic Literature Review

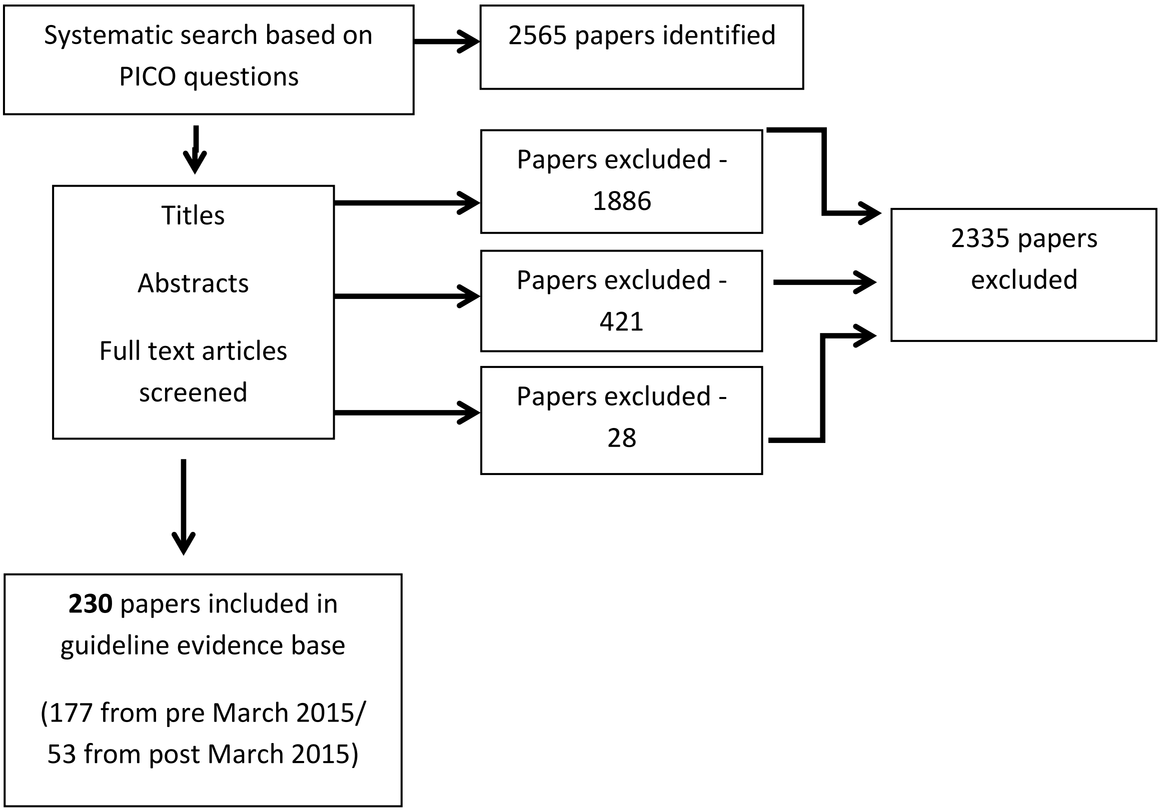

Appendix 4: Structured questions and answers in the literature and grade profiles

The quality of evidence and risk of bias was assessed using the GRADE approach (Guyatt et al, 2011). A member of the group was assigned to each subsection of the guideline based on their expertise to assess the quality of the evidence available for each PICO question. An initial grade (high, moderate, low or very low) was assigned to the evidence based on the overall study design and was increased or decreased based on the absence or presence of issues with study quality, consistency, directness of outcome measures, precision or reporting bias in accordance with GRADE guidelines. Two assessors reviewed each paper using this tool, and the mean score was calculated. A total of 258 papers were graded.

A decision to include or exclude each paper as evidence for a guideline recommendation was discussed for each section of the guideline by the GDG lead for that section, a senior member of the GDG and the GDG lead. The whole GDG then reviewed each guideline section and the supporting evidence to ensure GDG consensus for inclusion of all supporting evidence.

**Clinical Question**	**Reference**	**Type of Study**			**Quality of Evidence (Grading)**						**Comments**
		Type of study	No of patients		Risk of Bias (not serious, serious, very serious)	Inconsistency (not serious, serious, very serious)	Indirectness (not serious, serious, very serious)	Imprecision (not serious, serious, very serious)	Publication Bias (not serious, serious, very serious)	Final Grade	
			Control	Intervention							

Do abnormal TFTs affect prognosis and therefore management in children with thyroid nodule? Relevance of thyroid auto-antibodies?	(Chiu et al., 2012)	Retrospective cohort	n/a	78	High -	UTC	ns	Wide confidence intervals -	ns	Low (upgraded from very low)	Thorough analysis has shown a link between increased TSH and papillary thyroid cancer risk. There is a dose response gradient as increasing TSH is associated with an increased cancer risk and potential confounders have been taken into account. This study could be upgraded to LOW because of this. USA population 1997 - 2011. TSH level > /= 2.5 mIU/l OR of any cancer 8.05 (1.41-81.4) and of papillary carcinoma OR 12.75 (2.1-130.57)
	(Papendieck et al., 2011)	Retrospective cohort	n/a	45, 80% girls	high -	ns	ns	UTC	ns	Very Low	Thyroid function at diagnosis: normal 31, hypo 2, subclinical hyper 2. Median TSH 1.7 with no CLN and 2.78 with CLN mets
	(Mussa et al., 2013)	Multicentre retrospective cohort	68 no surgery	57 surgery	high -	UTC	ns	ns	ns	Low	pts diagnosed between 2006 and 2012 in 9 centres in Italy; TSH mean TSH 2.01 +/- 1.03 (median 1.6 range 0.4-8.4) in pts with thyroid cancer TSH mean 3.23 +/- 1.59 (median 2.85 range 1.4-8.4) without cancer mean TSH 1.64 +/- 0.99 (median 1.4 range 0.41-6) p< 0.001

	(Suzuki et al., 2016)	Prospective population study 2011-2014		2241 with thyroid nodules	low	ns	ns	s	ns	Very Low	Thyroid function tests cannot reliably distinguish between functional and malignant thyroid nodules
	(Verloop et al., 2012)	Systematic review of cohort studies and meta-analysis	32 studies		ns	ns	ns	s	ns	Low	
Is FNAC more informative when guided by US in comparison with 'free hand' biopsy?	(Izquierdo et al., 2009)	Retrospective study		42,8-29 yrs	low number	ns	ns	adults and children	ns	Very low	Clearly improves inadequacy rate, and specificity
	(Moudgil et al., 2016)	Retrospective study		70 children, 86 nodules	small	ns	ns	ns	ns	Very low	Diagnoses at cytopathology and surgical pathology were concordant in 27/29 (93.1%) nodules; no US-FNAB procedure yielded false-positive or false-negative results for malignancy

Role of pre operative FNA for diagnosis of DTC in children	(Bargren et al., 2010)	Retrospective study		110 pt; <19yrs	preselected surgical candidates (ns)	ns	ns	ns	ns	Moderate	
	(Hosler et al., 2006)	Retrospective study		82 pt ; (101FNAs); <18yrs	preselected patient group	ns	ns	ns	ns	Moderate	
	(Khurana et al., 1999)	Retrospective study		57pt; <20yrs	preselected patient group	ns	ns	ns	ns	Moderate	Bethesda (Thy1-5) not used
	(Rossi et al., 2014)	Retrospective study		64/247 pt <18 yrs	preselected surgical candidates (ns)	ns	ns	ns	ns	Moderate	ICC to evaluate suspicious lesions (HMBE1+ and galectin 3+)

	(Redlich et al., 2012)	Retrospective study		50pt; <17yrs	preselected by FNA	ns	ns	ns	ns	Moderate	
	(Hoperia et al., 2010)	Retrospective study		707 FNA, 165 surgery	surgery bias	different suspicious group, includes FN	ns	ns	ns	Low-moderate	ICC (MoAB47) in suspicious lesions
	(Sugino et al., 2013)	Retrospective cohrot	737		ns	ns	ns	s	ns	Low	
	(Altincik et al., 2010)	Retrospective study		30 FNAC	Small numbers	ns	ns	ns	ns	Low	

	(Corrias et al., 2001)	Retrospective study		42pts, 8-17yrs	surgery bias	ns	ns	ns	ns	Moderate	FNA highest sensitivity compared to other modalities (imaging, clin, lab)
	(Smith et al., 2013)	Retrospective study		68pts, 4-20yrs	surgery bias	ns	ns	ns	ns	Moderate	F/U of 50 (73%) showed 50/50 chance of predicting malignancy (would have been better if neoplasia had been considered)
	(Kapila et al., 2010)	Retrospective study		745 aspirates, 4-21yrs	surgery bias, large numbers lost to F/U	ns	ns	ns	ns	Low-moderate	Most aspirates benign
	(Raab et al., 1995)	Retrospective study		64aspirates, 1-18yrs	selection bias	ns	ns	ns	ns	Low	Most aspirates benign

	(Stevens et al., 2009)	Meta-analysis			surgery bias	S	ns	ns	ns	Low-moderate	Sensitivity 94% (86-100%); Specificity 81% (72-91%) FA counted as benign and as neoplasm
	(Canadian Pediatric Thyroid Nodule Study, 2008)	Retrospective study		141pts, 31-217months	algorithm results in surgery for all nodules	ns	ns	ns	ns	Low	
	(Baş et al., 2012)	Retrospective cohort		46	High -	UTC	Serious -	UTC	ns	Very low	Diagnostic value of FNA thyroid nodule - identifies malignancy; Diagnostic value of nodule FNA - gives adequate sample (SW) Identified 2 out of 4 cases of thyroid malignancy

	(Papendieck et al., 2011)	Retrospective cohort		45, 80% girls	high -	ns	ns	UTC	ns	Very Low	FNAB 39 undertaken 37 obtained diagnostic material; result of FNAB malignant 15, indeterminate 14, benign 8 (false negatives)
	(Mussa et al., 2013)	Multicentre retrospective cohort	68 no surgery	57 surgery	high -	UTC	ns	ns	ns	Low	Accuracy of FNA 100% sensitive, 58% specific, 77% diagnostic accuracy
	(Gutnick et al., 2012)	Single institution	21, all female, age range 12-17 yrs		high -	UTC	ns	UTC	ns	Very low	FNA performed in 10 pts (papillary thyroid cancer 1, atypical / suspicious 3, follicular neoplasm 1, haemorrhagic cysts 1, benign colloid nodule 4).
	(Saavedra et al., 2011)r	Retrospective observational, single cente		35 children <18yrs, all had surgery	ns	ns	S	S	S	Very low	FNAC without US guidance, separate US - cancer in 18 of 37 pts, 51% 'The global accuracy of FNAB was 83%, with a sensitivity of 75% and a specificity of 94%. 14 FNABs suggested malignancy (40%), only 1 of which was a false positive (7%). By contrast, 5 of the 21 FNABs suggesting benign lesions were false negatives (24%).'

	(Cole and Wu, 2014)	Retro cohort via computerized search		389<br><13 yrs	ns	ns	s	ns	ns	Very Low	FNA: sensitive (97.2%) and specific (98.5%), and has high positive predictive (97.2%) and negative predictive (98.5%) values for diagnosing malignancy in childhood
	(Lale et al., 2015)	Retrospective cohort		282	ns	ns	slight	ns	ns	low	
	(Norlen et al., 2015)	Retrospective review		56<br><18 yrs	ns	ns	ns	ns	ns	low	
	(Partyka et al., 2016)	Retrospective analysis		186 thyroid FNA samples from 154 patients <18 yrs	ns	ns	ns	ns	ns	low	FNA accuracy was 99%, and the sensitivity and specificity were 94% and 100%, respectively.

	(Francis et al., 2015)	ATA Paediatric Guidelines			ns	ns	ns	ns	ns	High	
Age cut off for tolerating FNA under local anaesthesia	(Redlich et al., 2012)	Retrospective study		50 preoperative FNA samples	ns	ns	Not main aim of study	ns	ns	Moderate	
Should molecular testing be used for diagnosing malignancy on cytology samples?	(Monaco et al., 2012)			179 aspirate, 66 with molecular data	ns	ns	ns	ns	ns	Low-moderate	Low numbers, 17% tested, 11 positive cases, no benign cases tested
Age of presentation of DTC. Average and range	(Steliarova-Foucher et al., 2006)	Retrospective - Data from ACCIS database 1988-97<br>Belarus analysed separately<br>Separate UK data available		1690 cases of chidhood and adolescent thyroid ca	ns	ns	ns	ns	ns	Low	Demographics:<br>Incidence 0.5-1.2 per million 0-14yrs<br>4.4-11 per mill 15-19yrs<br>65% PTC in children, 77% in adolescents

	(Farahati et al., 1998)	Retrospective questionnaire from 65 centres, multicentre cohort study		114<br>3-18 yrs	high	ns	ns	unlikely	unlikely	Very low	Mean age diagnosis 13.6 yrs girls, 12.4 yrs boys
	(O'Gorman et al., 2010)	Retrospective study		61pts	low number	ns	ns	ns	ns	Low	More metastases in younger children & Risk factor data
	(Harach and Williams, 1995)	National retrospective cohort 1963-1992		154	Low	ns	ns	Unlikely	Unlikely	Moderate	Upgraded as national cohort.<br>Age 5-9 17 male, 14 female<br>Age 10-14 27 male, 84 female
	(Machens et al., 2010)	retrospective cohort study, single centre		83 (57 female, 26 male)	high -	UTC	ns	UTC	ns	Very low	Association between age and extrathyroid tumour growth - older children have bigger volume extrathyroid growth tumours

	(Klein Hesselink et al., 2016)	Retrospective cohort 1970-2013		170	low	ns	ns	s	unlikely	low	
	(Silva-Vieira et al., 2015)	Retrospective regional cancer registry cohort 1964-2006		93 <18 yrs	low	ns	ns	ns	unlikely	low	
What is the incidence of children with DTC and is it on the rise?	(Vergamini et al., 2014)	Retrospective study - Dana Faber SEER study, 1984-2010; pts <30yr		< 30 yrs	ns	ns	most patients non-pediatric	ns	ns	Very low	Incidence of DTC is increasing in children and adolescents.<br>Larger tumours in children, increasing trends in female children: APC 2.76, 95% CI 1.01-4.04

	(Steliarova-Foucher et al., 2006)	Retrospective study		1690	ns	ns	ns	ns	ns	Low	
	(Al-Qurayshi et al., 2016)	Partial population - cross sectional analysis		644<br>mean age 13.8y	low	ns	ns	ns	unlikely	Moderate - upgraded one as partial population study	
	(Golpanian et al., 2016)	Partial population (SEER) 1973-2011		2504	low	ns	ns	ns	unlikely	moderate – upgrade as partial population study	

	(Dermody et al., 2016)	partial population (SEER) 2007-2012		1723	low	ns	ns	ns	unlikely	moderate - upgraded as partial population study	
	(Lerner and Goldfarb, 2015b)	partial population (SEER) 1988-2009		1825	low	ns	ns	ns	unlikely	moderate - upgraded as partial population study	
What is the incidence and prevalence of DTC in males vs females	(Steliarova-Foucher et al., 2006)	Retrospective study		1690	ns	ns	ns	ns	ns	Low	

	(Vriens et al., 2011)	Retrospective study of 1011 records; Part of SEER study		445 AYA	ns	ns	mostly patients adult	ns	ns	Very low	Genotyping of somatic mutations; Demographics:<br>83% female<br>94% TNM stage 1 disease<br>31% lymph node metastases<br>4% distant mets
	(Harach and Williams, 1995)	National retrospective cohort 1963-1992		154	Low	ns	ns	Unlikely	Unlikely	Moderate	Sex ration 108 female: 46 male; Sex ratio differentiated follicular carcinoma F:M 1.2:1 in under 10 yrs, 3.6:1 in 10 and over
	(Fridman et al., 2012)-	Retrospective cohort from population Cancer Sub-registry		94<br>age 4-18	high	ns	ns	ns	ns	Very low	Fourfold predominance in females
	(Trahan et al., 2016)	retrospective single centre cohort study - 1993-2014		125	low	ns	ns	ns	unlikely	low	

What is the incidence and prevalence of DTC in different ethnic groups	(La Quaglia et al., 2000)	Retrospective study		83	low	ns	ns	ns	unlikely	Very low	
What is the prevalence of thyroid nodules in children?	(Papendieck et al., 2015)	single centre retrospective cohort 2008-2013		75 <19 yrs	low	ns	ns	s	unlikely	very low	
What is the incidence of thyroid cancer in thyroid nodules found in children?	(Raab et al., 1995)	Retrospective cohort		57 pts	low number	ns	ns	ns	ns	Very low	18% malignancy in paediatric thyroid nodules
	(Kiratli et al., 2013)			50 (33 female, 17 male)	high -	UTC	Serious	UTC	ns	Very low	Presenting symptoms: thyroid nodule 16, goitre 16, neck swelling 14, dysphonia 2, dyspnoea 1;

	(Machens et al., 2010)	Retrospective cohort study, single centre		83 (57 female, 26 male)	high -	UTC	ns	UTC	ns	Very low	Association between multifocal tumours, extra-thyroid tumour growth and lymph node metastases - bigger, multifocal tumours more likely to have multiple lymph node metastases.
	(Canadian Pediatric Thyroid Nodule Study, 2008)	Retrospective, observational, 9 centres		141 children (DTC and MEN2)	ns	ns	ns	ns	ns	Low	The risk of malignancy in nodule is high (43%)
	(Ly et al., 2016)	single centre retrospective cohort 2003-2014		31	low	ns	s	s	unlikely	very low	

	(Trahan et al., 2016)	retrospective single centre cohort study - 1993-2014		125	low	ns	ns	ns	unlikely	low	
	(Jiang et al., 2016)	retrospective single institution study 2001 - 2010		83	low	ns	ns	ns	unlikely	low	
	(Bhatti et al., 2010)	Retrospective case-control study		119	ns	ns	ns	s	ns	Low	Childhood Cancer Survivor Study using the most current cohort follow-up to 2005
	(Corrias and Mussa, 2013)	Review			-	-	-	-	-		

	(Taylor et al., 2009)	Retrospective BCCSS study	50		ns	ns	ns	s	ns	Low	
What is the prevalence of papillary, follicular and rare aggressive histological sub-types of DTC?	(Steliarova-Foucher et al., 2006)	As above									
	(O' Gorman et al., 2010)	As above									
	(Koo et al., 2009)	Retrospective cohort		68	low numbers	ns	<20yrs	ns	ns	Very low	41% diffuse sclerosing variant of PTC - Includes good summary table of other demographic studies

	(Kiratli et al., 2013)			50 (33 female, 17 male)	high -	UTC	Serious -	UTC	ns	Very low	papillary carcinoma 36, papillary carcinoma with follicular variant 10, follicular carcinoma 3, mixed papillary and follicular carcinoma 1.
	(Harach and Williams, 1995)									Low	Diagnosis:<br>41% papillary Ca<br>13% papillary – follicular<br>17% follicular<br>14% medullary<br>5% adenoCa or Ca unspecified<br>5% teratoma<br>3% lymphoma
	(Corrias et al., 2010)	Multicentre retrospective cohort		120	ns	high	ns	ns	unlikely	Very low	63 pts surgery – 53% goitrous nodule, 22% papillary Ca, 13% follicular adenoma, 5% follicular Ca, 3% medullary Ca, 2% benign teratoma.
	(Lerner and Goldfarb, 2015a)	retrospective partial population (SEER) 1988-2009		1956	low	ns	ns	ns	unlikely	moderate - – upgrade as partial population study	

Which children with genetic mutations are at high risk of developing DTC?	(Yamashita and Saenko, 2007)	Review paper			ns	ns	ns	ns	ns	Very low	Mentions genetic syndromes (Gardner (APC), Cowden (PTEN)) etc; also mentions congenital goitre (Thyroglobulin gene). All are AD with variable penetrance
	(Richards, 2010)	Review paper			-	-	-	-	-	N/a	5% NMTC is familial- mentions Gardner, Pendred, Werner, Carney, Cowden. DTC risk 1-18% for these disorders, most of this risk will occur in adults with good prognosis
	(Morrison and Atkinson, 2009)	Review paper			-	-	-	-	-	N/a	6% of all NMTC DTC are familial, some kindreds present with mixed PTC and FTC features. Mentions advocating risk reducing thyroid surgery in some PTEN/PRKAR1A/SDHB and SDHD/MNG1=DICER1
	(de Kock et al., 2014)	Case reports		3	s	s	ns	s	ns	Very Low	

	(Stewart et al., 2019)	Retrospective cohort study		102	ns	ns	ns	s	ns	Low	
	(Lauper et al., 2013)	Retrospective case report review		189	s	ns	s	s	ns	Very low	
	(Marsh et al., 1998)	Retrospective cohort	42		s	s	ns	s	ns	Very low	
	(Pei ling Yang and Ngeow, 2016)	Review			-	-	-	-	-		

	(Rutter et al., 2016)	Case reports	10		s	ns	ns	s	ns	Very low	
	(Stratakis et al., 1997)	Retrospective cohort	53		s	ns	ns	s	ns	Very low	
	(Vriens et al., 2009)	Review			-	-	-	-	-		
How common is DTC and its subtypes in high risk populations?	(Richards, 2010)	Literature review			population bias	-	-	-	-	N/a	Mentions genetic syndromes (Gardner (APC), Cowden (PTEN)etc; also mentions congenital goitre (Thyroglobulin gene). All are AD with variable penetrance

	(Lazar et al., 2009)	Retrospective cohort		27	HIGH -	UC	ns	UTC	ns	Very low	Israeli population with high frequency of familial DTC and familial malignant syndromes children diagnosed between 1990 and 2008
How common are mutations in children with DTC?	(Yamashita and Saenko, 2007)	Retrospective literature review			post radiation exposure (Chernobyl)	ns	ns	ns	ns	Low-moderate	PTC 67-98%, FTC 4-23%, poorly differentiated rare (<0.1%) MTC 2-8%, or 17% after genetic testing
	(Suchy et al., 1998)	Retrospective study		small study (34 patients, no controls)	post radiation exposure (Chernobyl)	ns	ns	ns	ns	Very low - low	
	(Sassolas et al., 2012)	Retrospective study			ns	ns	ns	ns	ns	Low	Indirect evidence that only RET/PTC alterations play a role in paediatric PTC

	(Penko et al., 2005)	Retrospective study		14 patients, 10-21 years	ns	ns	ns	ns	ns	Low-moderate	No BRAF mutations identified
	(Hess et al., 2011)	Case control frequency matched design observational study			ns	ns	ns	ns	ns	Low	
	(Leeman-Neill et al., 2013)	Retrospective study		62 pts, <18yrs	radiation exposure	ns	ns	ns	ns	Low-moderate	RET/PTC most common (22%) but no known mutations 40%
	(Fenton et al., 2000)	Retrospective study		33 pts, 6-21yrs	sporadic cancers?	ns	ns	ns	ns	Low	Ret/PTC1 most common (45%), different from radiation (RET/PTC3 most common)

	(Gertz et al., 2016)	cohort study		14	ns	ns	ns	s	ns	low	
	(Prasad et al., 2016)	cohort study		28 <19 yrs	ns	ns	ns	ns	ns	low	
	(Finkelstein et al., 2012)	Case-control	34 BRAK V600E mutation-positive	22 mutation negative	ns	ns	ns	s	ns	Very low	
PTEN	(Smith et al., 2011)	Retrospective study		7 patients	germ line mutation	includes follicular adenomas	ns	ns	ns	Very low	

	(Ngeow et al., 2011)	Prospective study, small paediatric population (19 patients/664)			germ line mutation	other clinical markers	ns	ns	ns	Low	Recommends U/S from age 6 in known syndromes; screening in children with macrocephaly and DTC.
RET	(Fenton et al., 2000)	Retrospective study		33 pts, 6-21yrs	sporadic cancers?	ns	ns	relatively short F/U	ns	Very low-low	no significance re: stage and/or outcome
N-TRK	(Beimfohr et al., 1999)	Retrospective study		81 patients		small sample	ns	ns	ns	Very low	NTRK1 mutation rare in radiation induced PTC post Chernobyl; RET negative tumours (although RET mutated included: 9 patients)

What are criteria for diagnosing malignant follicular and papillary neoplasms?	(Horeria et al., 2010)	Retrospective	0	54 (of 707)	7.5% of the population previous exposure to radiation in Chernobyl	ns	ns	ns	ns	Low	The study may not be applicable in our setting.
	(Nikiforov et al., 2016)	International multidisciplinary, retrospective study	109 non-invasive	101 invasive <18 yrs	ns	ns	ns	ns	ns	low	

Which molecular markers (panel of markers) should be used to diagnose thyroid cancer? (BRAF, PTEN, p53, RET)	(Penko et al., 2005)	Retrospective	0	14/27	ns	Yes	ns	Small sample size of 14	ns	Very low	Penko et al analysed BRAF wild type in localised PTC disease in a small sample size whereas Henke et analysed BRAFV600E in PTC in all stages of PTC in size of 27 cases. assessed the more common BRAF mutations but not the less common ones, did not contain cases of PTC with mets where BRAF mutations are common in the adult population/ BRAF V600E mutation status did not correlate with pt and tumour characteristics although it was common compared to adult population where BRAF mutations are commoner/ BRAF v600E status was assessed in under 22, and the status was only seen in the paediatric population compared to pt and tumour characteristics
	(Henke et al., 2014)	Retrospective cohort	27		ns	s	ns	ns	ns	Very low	

	(Ricarte-Filho et al., 2013)	Retrospective cohort	23		s	ns	ns	s	ns	Very low	
	(Rosenbaum et al., 2005)	Retrospective cohort	20		ns	ns	s	ns	ns	Very low	
Should TNM system be used for tumour staging?	(Oommen et al., 2008)	Prospective case series	82	82	ns	s	s	ns	ns	Very low	The study compares 1997 TNM to 2002/03 TNM. There is no direct comparison with any other classification like AGES, MACIS. Age is under 18 variable extent of surgery, pts from various institutions, tumour size available in 69/82 pts so T staging inaccurate in 13 p no direct comparison with MACIS or AGES

	(Zimmerman et al., 1988)	Retrospective cohort	58		ns	ns	ns	ns	ns	Low	
Can histology be used to help define initial disease severity and/or risk of recurrence?	(Powers et al., 2004)	Prospective case series	0	48	ns	ns	no real comparator	ns	ns	Low	MACIS scores studied in 48 pts under 21 yrs with cut off of 4 to predict recurrence and persistence.
	(Mihailovic et al., 2014)	Retrospective	0	51	ns	ns	no real comparator	ns	ns	Low	
	(Jang et al., 2012)	Retrospective	0	66	ns	ns	no real comparator	ns	ns	Low	under 21 yrs old

	(Balachandar et al., 2016)	Retrospective review		62	ns	ns	ns	s	ns	very low	
	(Lee et al., 2015)	single centre retrospective cohort 1980-2013		150	low	ns	ns	s	unlikely	very low	
Should molecular testing be used for diagnosing malignancy on histology samples?	(Suchy et al., 1998)	Retrospective	0	34	Population bias, plus ages not specified in paper	ns	ns	ns	ns	Very low	Discussion around penetrability of mutations, no mention about if mutations present in surrounding thyroid, may not be applicable to non-radiation induced PTCs
	(Yamashita and Saenko, 2007)	Retrospective	556	455	ns	ns	s	ns	ns	Low	Candidate genes identified but found no differences in somatic mutations in AYA and adults

	(Patel, 2002)	Retrospective	0	32	ns	ns	ns	Yes	ns	very low	Positive expression of sodium ion importer is associated with low recurrence in PTC and FTC
	(Leeman-Neill et al., 2013)	Retrospective	0	62	Yes post Chernobyl radiation exposure cases	ns	ns	ns	ns	Moderate	These results provide the first demonstration of PAX8/PPARγ rearrangements in post-Chernobyl tumors and show different associations for point mutations and chromosomal rearrangements with (131) I dose and other factors. These data support the relationship between chromosomal rearrangements, but not point mutations, and (131) I exposure and point to a possible role of iodine deficiency in generation of RET/PTC rearrangements in these patients. Retrospective cohort study but shows dose response gradient with regards to I131 radiation exposure and positive correlation with chromosomal rearrangements but negative association with point mutations. So to be upgraded from low to moderate evidence
	(Fenton et al., 2000)	Retrospective	0	33	ns	ns	ns	ns	ns	Low	Authors conclude that ret/PTC mutations are 1) common in sporadic childhood PTC, 2) predominantly PTC-1, 3) frequently multiple, and 4) of different distribution than that reported for children with radiation induced PTC

	(Beimfohr et al., 1999)		0	81	Yes post Chernobyl	ns	ns	ns	ns	Low	
	(Hess et al., 2011)	Case control frequency matched design observational study	28	52	Yes post Chernobyl	ns	ns	ns	ns	Low	This study, using matched exposed and unexposed cohorts, provides insights into the radiation-related carcinogenesis of young-onset PTC and, with the exposure-specific gain of 7q11 and overexpression of the CLIP2 gene, radiation-specific molecular markers.
	(Cordioli et al., 2016)	Case control	30	15	ns	ns	ns	s	ns	Low	
	(Ballester et al., 2016)	Retrospective cohort		27 <20 yrs	ns	ns	ns	ns	ns	low	

	(Buryk et al., 2015)	Retrospective		89 <20yrs	s	ns	ns	s	ns	very low	
What is the sensitivity and specificity of thyroid US in detecting thyroid nodules and differentiating benign and malignant pathology?	(Corrias et al., 2010)	9 Italian centres, retrospective cohort		120 children < 18yrs with thyroid nodules had US	ns	ns	ns	s	ns	Very low	77 benign, malignant 19
	(Goldfarb et al., 2012)	Retrospective cohort study; Single center Miami, 2002, 2011		50 children < 21yrs, all had thyroidectomy	s	ns	ns	ns	s	Low	All USs performed by endocrine surgeons - conclude that clinic based US is useful tool for determining malignancy in thyroid nodules
	(Saavedra et al., 2011)	Retrospective observational 2001-2007 - Single center, Montreal, Canada		35 children <18yrs, all had surgery	ns	ns	s	s	s	Very low	FNAC without US guidance, separate US - cancer in 18 of 37 pts, 51% 'When FNAB suggested a benign lesion, US had a good sensitivity (80%) but a poor specificity and accuracy (50 and 57%, respectively); its negative predictive value was 90% and its positive predictive value 36%.'

	(Al Nofal et al., 2016)	Systematic review and meta-analysis		750 nodules	low	ns	ns	s	unlikely	moderate - downgraded due to imprecision	The presence of internal calcifications and enlarged cervical lymph nodes were the US features with the highest likelihood ratio [4·46 (95% CI: 1·87–10·64) and 4·96 (95% CI: 2·01–12·24), respectively] for thyroid cancer. A cystic nodule was the feature with highest likelihood ratio for benign nodules [1·96 (95% CI: 0·87–4·43)].
	(Koltin et al., 2016)	Retro cohort		27 subjects	s (small study)	ns	ns	ns	ns	Very low	Larger size, microcalcifications and ill-defined margins on ultrasound demonstrate the best predictive model for malignancy in the paediatric population
	(Perros et al., 2014)	Adult guideline			ns	ns	ns	s	ns	Moderate	
Does US help avoid multiple operations?	(Gupta et al., 2013)	retrospective, observational- Boston, 2 centres, 1997-2011		125 patients < 18 yrs had US and FNAC	ns	ns	ns	ns	ns	Low	benign 78%, malignant 22 % (in comparison with 2582 adults 14%) - Avoid biopsy in subset of patients post us

	(Abbasian Ardakani et al., 2018)	Prospective comparison	170 metastatic	170 tumor-free LNs	ns	ns	S	ns	ns	Low	
Does US help identify best nodule for biopsy?	(Gupta et al., 2013)	As above									
	(Corrias et al., 2010)	9 Italian centres, retrospective cohort		120 children < 18yrs with thyroid nodules had US	ns	ns	ns	s	ns	Very low	

What are typical sonographic features of thyroid cancer?	(Lyshchik et al., 2005)	retrospective, observational, Minsk		103 children < 18yrs	ns	s	ns	s	ns	Very low	35 had cancer, 68 benign; gold standard: histology, cytology, F/U; Nodules smaller than 15 mm had higher accuracy in diagnosing cancer than larger nodules; irregular outline, subscapular location, vascularisation good predictors Hypoechogenecity and microcalcifications were not
	(Goldfarb et al., 2012)	Retrospective cohort study; Single centre Miami, 2002, 2011		50 children < 21yrs, all had thyroidectomy	s	ns	ns	ns	s	Low	Univariate analysis - microcalcification, abnormal LN and dimensions taller than wide were significant predictors of malignancy
	(Corrias et al., 2010)	9 Italian centres, retrospective cohort		120 children < 18yrs with thyroid nodules had US	ns	ns	ns	s	ns	Very low	
	(Papendieck et al., 2011)	retrospective cohort		45, 80% girls	high -	ns	ns	UTC	ns	Very Low	US characteristics thyroid nodules solid 32, solitary 27, heterogeneous 11, multiple 10, hypoechogenic 9, soli-cystic 5, irregular margins 5, pathological nodes 5 microcalcification 3, diffuse goitre 2

	(Mussa et al., 2015)	Prospective		184	low	low	Includes 4 cases non PTC	ns	ns	low	A multivariate analysis confirmed that microcalcifications, hypoechoic pattern, intranodular vascularization, lymph node alterations, and thyroid stimulating hormone concentration were independent predictors of malignant outcome.
Should neck and chest CT scan be performed in all children assessed for thyroid cancer?	(Kim et al., 2011)	retrospective, observational - single center, Cincinatti		13 children < 17rs	ns	ns	ns	s	s	Very low	Comparison of planar and SPECT/CT for I 123, 131 after TT - SPECT better
How often should US be performed?	(Antonelli et al., 2003)	Retrospective, observational, Pisa , 1 center		45 children < 18 yrs old, all cancer	ns	ns	ns	ns	ns	Low	Comparison of palpation, WBS, Thyroglobulin and US detecting recurrence during follow up
What are the indications for radioisotope or US scanning?	(Bal et al., 2004)	Retrospective, observational, 1967-2002 - New Delhi, single institution		122 children <20 yrs, 28 of them had pulmonary metastases	ns	ns	ns	ns	ns	Low	Post op: 21 had normal chest xray but all 28 abnormal WBS - Of 21 with N chest X ray CT detected mets only in 5 (21%)

	(Solt et al., 2000)	prospective follow-up study		45 long-term survivors of histology-proved childhood lymphomas	ns	ns	ns	s	unlikely	Low	Thyroid gland abnormalities were detected in 54% of HD survivors after mantle field irradiation
	(Soberman et al., 1991)	cohort study		18 HD survivors, all of whom received radiation therapy	ns	ns	ns	s	unlikely	Low	Ultrasound detected abnormalities in 16 patients (89%)
	(Mostafa et al., 2016)	Retrospective cohort		53 mean age 13.3 years	ns	ns	ns	likely	ns	very low	Variants and pitfalls that mimic functioning thyroid tissue or functioning thyroid carcinomatous tissue on radioiodine scan in children with differentiated thyroid carcinoma are not infrequent, but they decrease in frequency on successive radioiodine scans.
What is the best imaging modality to detect local recurrence?	(Antonelli et al., 2003)	As above									

	(Markovina et al., 2014)	Retrospective observational, 1969-2009 - St Louis, USA, 1 center		112 children < 22 yrs	ns	ns	ns	s	ns	Very low	Overall survival: 20 yrs 100%, 30 yrs 94.4 - PFS 10 yrs 71% 20 yrs 62%, 30 yrs 55%
	(Vali et al., 2015)	Retrospective cohort		40	ns	ns	ns	ns	ns	low	The sensitivity was 85.7%, specificity 89.4%, negative predictive value 94.4% and positive predictive value 75% for US in detecting loco-regional recurrence in follow-up studies of paediatric differentiated thyroid cancer. In 17.3% (18/104) of studies, the results of stimulated thyroglobulin and US were discordant.
	(Durante et al., 2013)	Review			-	-	-	-	-	-	
What is the best imaging modality for detecting distal metastasis?	(Bal et al., 2004)	As above									

	(Vassilopouloii-sellin et al., 1993)	Retrospective, 1960-1990		19/209 <25 yrs had lung mets (9%)	ns	ns	ns	s	ns	Very low	1/2 of lung mets were not seen on chest Xray but were seen on WBS
	(Markovina et al., 2014)	As above									
What are the indications for initiating treatment with radioiodine?	(Mallick et al., 2012a)	Randomised non-inferiority phase II/III multicentre trial									
What is the recommended dose of I131 for ablation and subsequent treatment?	(Pawelczak et al., 2010)	Systematic review 2010, 9/14 articles met inclusion criteria		112 children < 21 yrs with DTC and pulmonary mets	ns	ns	ns	ns	ns	Low	DS mortality 2.68%, survival 97.32%, 1/11 radiation fibrosis

	(Mallick et al., 2012b)	Randomized noninferiority trial	421		ns	ns	ns	s	ns	Moderate	Age range 16-80yrs
	(Pluijmen et al., 2003)	Retrospective cohort	59	61	ns	ns	ns	s	ns	Very low	
How should the dose be calculated taking into account patient weight/BMI/age/ tumour staging/	(Verburg et al., 2011)	Retrospective - Belarus 2011		180 children < 20yrs	ns	ns	ns	s	ns	Low	AHASA important in children with pulmonary metastases
	(Biko et al., 2011)	Case report		1	s	ns	ns	ns	s	Very low	AHASA determined by radiation exposure to critical organs (bone marrow, lungs)

	(Schlumberger et al., 2012)	Randomized, phase 3 trial	684		ns	ns	ns	s	ns	Moderate	Age ≥ 18yrs
Does treatment with I131 improve survival and reduce recurrence rate? What alternative therapies are available?	(Pawelczak et al., 2010)	Systematic review 2010, 9/14 articles met incusion criteria		112 children < 21 yrs with DTC and pulmonary mets	ns	ns	ns	ns	ns	Low	DS mortality 2.68%, survival 97.32%, 1/11 radiation fibrosis
	(Handkiewicz-Junak et al., 2007)	Observational study, Poland, National centre		235 children, <18 1973-2002	ns	ns	ns	ns	ns	Low/moderate	86% recurrence free 14% of children had local recurrence (TBR 28%, LNR 63%, both 9%). Age not related to recurrence NO DEATHS

	(Chow et al., 2004)	Observational 2004, Hong Kong 1960-1997		60 patients < 21 yr, follow up 14 yrs	ns	ns	ns	ns	ns	Low	RAI can reduce local recurrence rate
	(Verburg et al., 2015)	Prospective		76 <21 yrs	s	ns	ns	ns	ns	low	No patients died of DTC<br>Female gender, lower T stage and higher 131I activity (successful ablation, median activity 3.1 GBq, unsuccessful ablation 2.6 GBq) were determinants of a higher rate of successful ablation. After 131I therapy no patient showed recurrence after reaching CR or disease progression if CR was not reached.
	(Xu et al., 2016)	Retrospective	32 incomplete ablation	56 complete ablation	ns	ns	ns	ns	ns	very low	
	(Schlumberger et al., 2015)	phase 3, randomized, double-blind, multicenter study	131	261	ns	ns	ns	S	ns	moderate	Age ≥18 yrs

	(Lee et al., 2019)	Retrospective cohort		166	ns	ns	ns	ns	ns	Low	
	(Qichang et al., 2019)	Meta-analysis		1195 from 17 studies	s	ns	s	ns	s	Moderate	Studies were limited by high heterogeneity and possible publication bias, not limited to adults
Is rTSH administration better strategy than stopping thyroxine before administration of I131?	(Luster et al., 2009)	Retrospective Multiinstitutional 2009, Europe, Asia, Austr , 23 centres in 12 countries		100 children < 18yrs had rh TSH (0.9 mg on two consecutive days) or T4 withdrawal for 7 days	ns	ns	ns	ns	ns	Low-moderate	Adverse effects rare (88% had none); TSH levels good for both strategies

	(Kuijt and Huang, 2005)	Retrospective 2000-2005, Boston 2005		11 children < 18, withdrawal of T4	ns	ns	ns	ns	ns	Low	Only consider T4 withdrawal - 2 weeks withdrawal achieved good TSH levels (in adults recommended is 6 weeks)
	(Iorcansky et al., 2005)	Retrospective review, 2005, Sloan Kettering 1990-2002	19 rhTSH	53 children had T4 withdrawal	ns	ns	ns	ns	ns	Low-moderate	TSH levels similar in both groups<br>Pattern similar to adults (no dose adjustment)<br>no/ minimal side effect
	(Handkiewicz-Junak et al., 2015)	Retrospective cohort	48 (rhTSH) <18 yrs	82 after THW <18 yrs	s	ns	ns	ns	ns	low	48 patients were treated after rhTSH (rhTSH group) and 82 after THW group.<br>Peak TSH concentration was significantly higher in the rhTSH group (152 µIU/ml vs 91 µIU/ml). Similarly, the thyroglobulin concentration was higher in the rhTSH group (9.7 ng/ml vs 1.8 ng/ml). No side effects requiring medical intervention were recorded after rhTSH administration. The evaluation of disease outcomes during TSH stimulation (6-18 months after (131)I treatment) revealed equal rates of thyroid ablation (71%) in both groups

Efficacy and safety of recombinant TSH for thyroid remnant ablation with RI in children	(Rosario et al., 2012)	Cohort prospective - Brazil 2011		12 children < 16 yrs, papillary ca	ns	ns	ns	ns	ns	Very low	Good TSH levels, minimal side effects post rhTSH administered for ablation -> rhTSH can be used in children
How should serum calcium be monitored and which calcium supplementation should be used in children?	(Schneider et al., 2004)	Longitudinal observational study - Belarus 2004		91/208 children with post op hypoparathyroidism	ns	ns	ns	ns	ns	Low	Only boys had impact on bone mineralisation but supplementation corrected it; hypoparathyroid girls had the same mineralisation as girls with normal PTH
	(Freire et al., 2014)	Cohort study	32		s	ns	ns	ns	ns	Very low	
	(Patel et al., 2018)	Retrospective cohort	23	30	ns	ns	ns	ns	ns	Low	

Which vitamin D supplementation should be used in children?	(Schneider et al., 2004)	As above									
How should thyroglobulin levels during TSH suppression be interpreted?	(Kirk et al., 1992)	Barts 1992, retrospective, 1983-1992		10 children <17	s	ns	ns	ns	s	Very low	TG can detect residual and metastatic and recurrent disease in children
	(Biko et al., 2011)	Observational, 2010, Belarus		20 children < 18 yr with DTC and lung metasases	ns	ns	ns	ns	ns	Low	Treated with RAI which was stopped after 5-6 courses (worry about fibrosis)<br>No progression observed, TG continued to decline despite positive WBS (? mechanism, late effects of RAI?)
What is the role and indication for 'stimulated' thyroglobulin measurement?	(Hoe et al., 2006)	Retrospective 200-2004 - Philadelphia 2006		7 <18yrs	s	ns	ns	ns	s	Very low	Recombinant Human TSH Stimulated Thyroglobulin Level useful for monitoring

	(Pacini et al., 2003)	Cohort study	340		ns	ns	ns	s	ns	Low	
	(Smallridge et al., 2007)	Retrospective cohort	194		ns	ns	ns	s	ns	Very low	
	(Spencer et al., 2010)	Cohort study	849		ns	ns	ns	s	ns	Very low	
What levels of Thyroglobulin are suspicious of recurrence?	(Kirk et al., 1992)	As above									

	(Dottorini et al., 1997)	Observational 1997, Italy 1958-1995		85 children < 18 with DTC	ns	ns	ns	ns	ns	Low	46 TT, 38 partial ST, biopsy (1), 47 lymphadenectomy<br>75 RAI, 21 EBR<br>All alive, 6 local recurrence, 4 lung mets<br>It is difficult to achieve undetectable TG but it continues to decline
	(Spencer et al., 1998)		4453	213	ns	ns	s	ns	ns	Very low	Control group mean age, 45.3 yr; range, 12–99 yr, DTC group mean age, 51 yr; range
Frequency and duration of follow-up	(Papendieck et al., 2011)	retrospective cohort		45, 80% girls	high -	ns	ns	UTC	ns	Very Low	recurrence occurred in 5 pts median time since treatment 3.3 years
	(Brose et al., 2012)	Report by multidisciplinary, multiregional panel of experts			-	-	-	-	-	Low	

	(Brose et al., 2014)	Multicentre, randomised, double-blind, placebo-controlled	210	207	s	ns	ns	s	ns	Moderate	Age > 18yrs<br><br>Sorafenib significantly improved progression-free survival compared with placebo in patients with progressive radioactive iodine-refractory DTC
	(Lee et al., 2019)	Mutlicentre review and metaanalysis		166	ns	ns	ns	ns	ns	Moderate	
Long term fertility impact/needs	(Wallace, 2011)	Review			-	-	-	-	-		
	(Alessandri et al., 2000)	retro review	38 <17yrs		yes (16pt records not available)	ns	ns	ns	ns	Low	Single centre, 1955-1996, recurrence defined as clinical or radiological, TNM 1987, two groups ≤10 and >10, no diff in OS, 100%. 20 yr RFS 10.1% <10 and 48.3% >10 p=0.008

Recurrence rate in patients with DTC with known mutation	(Borson-Chazot et al., 2004)	retro review	74 ≤20yrs		ns	ns	ns	ns	ns	Low	1985-2001, med age 17, 25 <15, med FU 61mth; Risk factors for reintervention were young age (< 15 years) (p < 0.01) and cervical lymph nodes (p < 0.001).
Recurrence rate in patients with DTC without known mutation	(Demidchik et al., 2006)	retro review	740 <15yrs		yes, population exposed to Chernobyl	ns	ns	ns	ns	Moderate	Upgraded due to size from low. Mean FU 115.8 mths, 1985-2003, Chernobyl area, 92% exposed, TNM 6th. Recurrence in 204 cases (27.6%),: 73 local relapses (9.9%), 90 distant metastases (12.2%), combination of local and distant in 41 (5.5%) MVA: independent parameters significantly associated with the risk of recurrent nodal disease: a young age at diagnosis, multifocal carcinomas, N1 status, and lack of neck lymph node dissection. For lung metastases, the significant risk factors were female gender, young age at diagnosis, and presence of symptoms.5- and 10-year survival for the entire group was 99.5% and 98.8%,

	(Welch-Dinauer et al., 1998)	retro review	169 ≤21yrs		ns	ns	ns	ns	ns	Low	Multifocality strongest predictor of recurrence; follow up only 6 yrs median
	(Dottorini et al., 1997)	retro review	85 <18yrs		ns	ns	ns	ns	ns	Very low	1958-1995, med FU 111mths
	(Enomoto et al., 2012)	retro review	142 <20yrs		ns	ns	ns	ns	ns	Low	1961-2005 series, 59/142 had EBRT and none had RAI ablation. Gross nodal disease, metastases, tumour diameter, ETE, and FHx all risk factors for DFS. Gross Lns pre-op and M1 disease risk for CSS. <16 more likely to have gross LN disease, Larger tumours and M1 disease
	(Hay et al., 2010)	retro review	215 <21yrs		ns	ns	ns	ns	ns	low	1940-2008, med 16yrs, med FU 29yrs, All-causes mortality rates did not exceed expectation through 20 years, but from 30 through 50 years, the number of deaths was significantly (P\0.001) higher than predicted. Fifteen of 22 deaths (68%) resulted from non-thyroid malignancy.

	(Bal et al., 2001)	retro review	80 <20yrs		s – 7% less than 20 yrs	ns	ns	s	ns	Very low	Pts from Indian low-iodine endemic regions
	(Huang et al., 2012)	retro review	116 ≤20yrs		ns	ns	ns	s	ns	low/very low	5- and 10-year progression-free survival rates 79.1% and 73.4% respectively; Cancer survival rates 99.1 and 96.5%
	(Jang et al., 2012)	retro review	66 <21		ns	ns	ns	ns	ns	Low	MACIS >4 predictive of poorer 10yr DFS
	(Jarzab et al., 2000)		109 aged 6-17yrs		ns	ns	ns	ns	ns	Low	On multivariate analysis total thyroidectomy and radioiodine improved recurrence free survival; Actuarial survival 100%<br>Actuarial DFS 5yrs 80%, 10yrs 61%.

	(Kiratli et al., 2013)	retro review	50 5-19yrs		ns	ns	s	ns	ns	Very low	The mean duration of follow-up was 77.6/12. Pts with M1 disease had worse DFS. Overall good prognosis
	(Kowalski et al., 2003)	retro review	38 4-18yrs		ns	s	ns	ns	ns	Very low	Small numbers, mixed path.
	(Landau et al., 2000)	retro review	30 <16		ns	ns	ns	ns	ns	Low	Median FU 22.5 yrs, The risk of recurrence was higher in children aged 10 years or younger [HR 3.45, 95% CI (1.04±11.11) P=0.03]. TSH suppression was the only intervention to be shown to reduce the recurrence rate [HR 11, 95% CI (2.27±50) P=0.0003]. The median overall survival is 53 years.<br>The only presenting feature predictive of poorer survival was the presence of metastases (HR 28.96, 95% CI 2.51±334, P<0.001).

	(Lazar et al., 2009)	retro review	27	10 prepubertal, 17 pubertal	ns	ns	ns	ns	s	Very low	Small numbers.<br>A positive family history of DTC was more prevalent in the pre-pubertal group (P .037). At diagnosis, they had more ETE, LN involvement and lung mets if pre-pubertal. Med FU only 5ys.
	(Markovina et al., 2014)	retro review	112 <22yrs		ns	ns	ns	s	s	Low	Overall survival at 20 years and 30 years was 100% and 94.4%, respectively. PFS at 10, 20, and 30
	(Mihailovic et al., 2014)	retro review	51 ≤20yrs		ns	s	ns	ns	ns	Low	Recurrence 16.7% at 5yrd; 22.3% at 10yr; 33.3% at 15 yrs. Factors predictive of recurrence: younger age at Dx, less radical Sx, no ablation RAI, tumour multifocality.

	(Palmer et al., 2005)	retro review	36 <18		ns	s	ns	ns	ns	Low	Single institution, Mayo 1987-2001, retrospective, cohort, observational<br>36 children <18yrs<br><br>LNs and multiple thyroid nodules are best predictors of recurrent disease but no survival. No deaths<br><br>Repeated operations required as recurrence rate high (47% as compared to adults 5-20%)
	(Patel, 2002)	retro review	31 ≤21 years		s	ns	ns	ns	ns	Very low	NIS expression confers superior prognosis<br>Those failing to stain for NIS more likely to recur and required higher cumulative dose of RAI to achieve remission.<br>But: short FU and small numbers though<br>Note likely that TSH stimulation for RAI might overcome the 'non-expressers'<br>Grade very low due to small numbers, retrospective
	(Papendieck et al., 2011)	retrospective cohort		45, 80% girls	high -	ns	ns	UTC	ns	Very Low	Recurrence occurred in 5 pts median time since treatment 3.3 years

	(Powers et al., 2003)	retro review	47 <21		ns	ns	ns	ns	ns	Low	Retrospective review, stage and primary tumour size correlate with remission following initial therapy. PTC only
	(Powers et al., 2004)	retro review	48 <21		ns	ns	ns	s	ns	Very low	Only 48/140 <21 had records thus possible selection bias, MACIS score <>4 predictive of recurrence or persistent disease. NPV 94 and 91% respectively. PPV not good.
	(Wada et al., 2009)	retro review	57 ≤15yrs		s	ns	ns	s	ns	Low	Factors for recurrence: male, T3/4, N+
	(Qu et al., 2016)	Regression analysis and metaanalysis	146 7-20 yrs		n	n	low	n	n	Moderate	Meta-analyses revealed that multifocality (HR = 1.91, P < 0.05) and presentation at diagnosis (HR = 1.39, P < 0.05) were highly associated with recurrence in young DTC patients.

	(Sugino et al., 2015)	PTC	227 <20 yrs		ns	ns	ns	s	ns	low	
	(Mihailovic et al., 2014)	retro review	51 ≤20yrs		ns	s	ns	ns	ns	Low	
Rates and specifics of morbidity in patients with DTC with known mutations											
Rates and specifics of morbidity in patients with DTC without known mutations	(Oren et al., 2012)	cross sectional case control	10-18yrs		ns	ns	ns	ns	ns	Low-moderate	QoL cf autoimmune thyroiditis controls. 16 vs 16. No difference and no diff with that expected in normal population

	(Palmer et al., 2005)	retro review	n=36 <18		ns	s	ns	ns	ns	Low	4% hypoPTH, 2%RLN injury
	(Vassilopoulou-Sellin et al., 1998)	retro review	n=112, <20		ns	ns	ns	ns	ns	Low	4% died from Rx related 2nd malignancy (2 breast ca, 2 sarcoma) 1 trach stenosis. Note EBRT delivered in 1950's.
	(Wada et al., 2009)	retro review	57 ≤15yrs		s	ns	ns	s	ns	Low	5.3% RLN injury, 10.5% HypoPTH
	(Kowalski et al., 2003)	retro review	38 4-18yrs		ns	s	ns	ns	ns	Very low	Small numbers, mixed path.<br>24% transient hypocalcaemia; 16% permanent hypocalcaemia; 5% vocal cord palsy;

Rates and specifics of mortality in patients with DTC with known mutations	(Brink et al., 2000)	retro review	14 ≤17yrs with M1 (lung)		retrospective	ns	ns	s	ns	Very low	young patients with pulmonary metastases from PTC do better than adults presenting with this stage disease
Rates and specifics of mortality in patients with DTC without known mutations	(Goldfarb and Freyer, 2014)	cohort review	15-39		retrospective	ns	ns	ns	ns	Low	No evidence of previous treatment, possible bias in data on database, mean FU short 35.5 months.<br>Worse OS in TC as SMN than those as primary. Not enough detail to know if this is due to previous Dx and treatment or the TC. Not really paediatric population as latency between first Ca and Rx.
	(La Quaglia et al., 2000)	Observational	83 children <21 with lung mets		ns	ns	ns	ns	ns	Low	No deaths, overall 10 yr survival 100%<br>PFS at 5 yrs 76%, 66% at 10 yrs

	(Fallah et al., 2013)	cohort	63495		ns	ns	ns	ns	ns	Moderate	Cohort but large numbers and difference. Increased risk of FNMTC if relative with TC. Suggest that surveillance with USS might be justified 5 country, population based cohort study
Family History	(Shayota et al., 2013)	retro review	895 <20		ns	ns	ns	ns	ns	Moderate	Upgraded in view of large numbers. MeSS is a useful prognostic tool on paediatric DTC where other tools used in adults fail.
	(Vassilopoulou-Sellin et al., 1998)	retro review	112 <20		ns	ns	ns	ns	ns	Low	low mortality 0% at 10yrs, but then 5-7% post 10 yrs

	(Jarzab et al., 2000)		109 aged 6-17yrs		ns	ns	ns	ns	ns	Low	17% permanent complications 11/109 laryngeal palsy, 6/109 hypoparathyroidism
	(Steliarova-Foucher et al., 2006)	retro review	1690 <19		ns	ns	ns	ns	ns	Moderate	>90% 20yr survival
	(Ito et al., 2012)	retro review	110 <20yrs		ns	ns	ns	ns	ns	Low	High incidence of larger nodes and primary in the <20; LN recurrence high and LN≥3cm and age <16 predictors of this; ≥3cm node and ETE predictors for M1; CSS was excellent

	(Sigurdson et al., 2005)	Nested case-control study	265	69	S	ns	S	S	ns	Very Low	Radiotherapy to thorax or head & neck, especially under 10 yrs of age; Chemo not relevant; Increased rates in Hodgkin's lymphoma & neuroblastoma Up to 20-29 Gy, not over 30 Gy Incudes adult patients (inclusion any patient dx with 1 ca under 21yrs of age) Total 25% study patients under 19yrs at DTC diagnosis. Large CIs e.g odds ratio for radiotherapy 20-29 Gy is 9.8 (3.2-34)
Whom should we be screening?	(Brignardello et al., 2008)	Retrospective Cohort study		129 cancer survivors	ns	ns	S	ns	ns	Very low	Time since follow-up 15.8yr (6.1-34.8) - Recommend US screening in childhood Ca survivors who had Rx to head/neck/thorax to detect non-palpable DTC<br>PTC in 5 patients Indirectness ++ age at study median 25.1 (17.5-43.8)
	(Veiga et al., 2012)	Retrospective - Study pooled two cohort and two nested case-control studies of childhood cancer survivors		187 developing primary thyroid cancer	ns	ns	ns	large Cis	ns	Low	Radiation dose-related RRs increased approximately linearly for <10 Gy, levelled off at 10-15-fold for 10-30 Gy and then declined, but remained elevated for doses >50 Gy. Dose-related excess RRs increased with decreasing age at exposure (P < 0.01), but did not vary with attained age or time-since-exposure, remaining elevated 25+ years after exposure.

	(Nikiforov and Gnepp, 1994)	Cohort study		84 children 5-14yrs	possible	ns	ns	ns	ns	Very low	Specific characteristics of DTC in children post Chernobyl
	(Metzger et al., 2006)	Retrospective cohort	67		s	ns	ns	s	ns	Very low	
	(Sigurdson et al., 2005)				s	ns	ns	ns	ns	Very low	Fall in risk at high does >30 Gy due to cell-killing effect

Which are the main conditions at risk e.g. ALL, Hodgkin's lymphoma?	(Veiga et al., 2012)	Pooled analysis - 2 case-control, 2 cohort	16.5k	187	ns	Diffs between the 4 studies, 1 study fitted different model (homogenity page 372)	S	ns	ns	Low	
	(Clement et al., 2015)	Retrospective observational		24	s	ns	ns	s	ns	Very low	
	(Solt et al., 2000)	As above									

	(Kennedy et al., 2014)	Retrospective cohort study		163 pts with FAP	ns	ns	All adult presentations of PTC	ns	ns	Very low	PTC in 3.1% - all presented as adults 19-23yrs; Propose yearly thyroid US from age 12yrs
Which are the familial syndromes requiring screening for DTC?	(Richards, 2010)	Review			-	-	-	-	-	N/a	Review paper - covers FAP, Carney, Cowden, Pendred, Werner 5% of DTC are familial 25% of MTC are familial
	(Smith et al., 2011)	case report			only 7 pts	ns	ns	ns	ns	Very low	5 developed Thy Ca 6-12yrs of age<br><br>Recommend annual Thy US after genetic Dx

	(Aretz et al., 2004)	Observational study	917		ns	ns	s	ns	ns	Low	
	(Sigurdson et al., 2005)	Nested case-control study		69 cases thyroid ca vs 265 matched controls without thyroid cancer	ns	ns	ns	ns	ns	Low-moderate	Up to 14 years follow up - confirms need for long term follow up for any child who had radiotherapy to thorax or head and neck region Risk of thyroid cancer increased with radiation doses up to 20-29 Gy (odds ratio 9.8 [95% CI 3.2-34.8]). At doses greater than 30 Gy, a fall in the dose-response relation was seen
How frequently should we be screening and with what investigations?	(Zivaljevic et al., 2013)	case control		75	ns	ns	Patients under 20yrs	only 4 patients of the 75 with PTC had FHx of thyroid Ca	ns	Very low	Paper makes recommendation to screen family members yearly with US

	(Brignardello et al., 2008)			129 cancer survivors	ns	ns	S	ns	ns	Very low	Suggests need US as only 2 of 5 who developed PTC had palpable nodules
	(Sigurdson et al., 2005)			69 cases thyroid ca vs 265 matched controls without thyroid cancer	ns	ns	ns	ns	ns	Low-moderate	
What constitutes the high risk for development of thyroid cancer in children?	(Zivaljevic et al., 2013)	retrospective 1995-2009 - case-control PTC only		75	ns	ns	up to 20yrs of age	only 4 patients of the 75 with PTC had FHx of thyroid Ca	unlikely	Very low	Logistic regression - uni and multivariate FHx only outcome statistically significant- FHx thyroid Ca OR 4.0 (1.4-9.5), p=0.045

	(Lazar et al., 2009)	Retrospective observational study from 1977		27	small study	ns	ns	ns	unlikely	Very low	Predisposing factors. Prepubertal 2 Hashimotos, 3 multi nodular goitre, 5 familial DTC. Pubertal 5Hashimotos, 6 multi nodular goitre, 3 familial DTC, 2 familial malignant syndrome, 2 post irradiation
	(Santos et al., 2017)	Observational study, partly propsective, partly retorspective		214	s	ns	s	ns	ns	Very low	Age range of pts 8-97yrs
	(Veiga et al., 2012)	Pooled analysis - 2 case-control, 2 cohort	16.5k	187	ns	Diffs between the 4 studies, 1 study fitted different model (homogenity page 372)	S	ns	ns	Low	Also declines over 30 Gy; increased risk with lower age at radiation and increased time from exposure - remained high >25yrs after; lower ERR/Gy in Hodgkins patients Mainly adults developing DTC - details of % children not given, only age ranges and median values for each study

Radiation (? dose, whole body, neck)). Length and exposure threshold	(Sigurdson et al., 2005)			69 cases thyroid ca vs 265 matched controls without thyroid cancer	ns	ns	ns	ns	ns	Low-moderate	
	(Leeman-Neill et al., 2013)	Cohort		62	Yes post Chernobyl radiation exposure cases	ns	ns	ns	ns	Moderate	Table 1 - age at exposure/ surgery/ time since exposure by mutation (13.5 - 18.6 yrs) 35% RET/PTC rearrangement, 15% BRAF point muts (V600E, 8% RAS point muts, PAX8/PPARg - 2 cases; Details re dose-response curve
	(Sigurdson et al., 2005)			69 cases thyroid ca vs 265 matched controls without thyroid cancer	ns	ns	ns	ns	ns	Low-moderate	Chemotherapy for the first cancer was not associated with thyroid-cancer risk, and it did not modify the effect of radiotherapy

Effect of chemotherapy	(Brignardello et al., 2008)	Cohort		129	ns	ns	Majority of pts >18yrs	ns	ns	Very low	
What is the time lag between treatment and development of DTC?	(Rose et al., 2012)	Case-control		49 pts with Thy Ca	ns	ns	All adult pts	ns	ns	Very low	Mean time to dx 19.5yrs<br><br>RR for radiation is 2.22 (1.15-4.29)<br>Highest for CNS cancer (4.47)<br>Lowest for leukaemia (1.75)
	(Baş et al., 2012)	Retrospective cohort		111	High -	UTC	Serious -	UTC	ns	Very low	The population is not representative of UK population as 44% had iodine deficiency. Risk factors for malignancy identified were prior neck irradiation and family history of thyroid cancer. Turkish population diagnosed 2007 to 2011.

What is the most common presentation of DTC in children?	(Lazar et al., 2009)	Retrospective cohort		27	HIGH -	UC	ns	UTC	ns	Very low	Study conducted in Israel, possible larger risk of familial cancer 1990 - 2008. Israeli population with high frequency of familial DTC and familial malignant syndromes children diagnosed between 1990 and 2008
	(Papendieck et al., 2011)	retrospective cohort		45, 80% girls	high -	ns	ns	UTC	ns	Very Low	1998 to 2008 Argentina
	(Kiratli et al., 2013)	Retrospective cohort		50 (33 female, 17 male)	high -	UTC	Serious -	UTC	ns	Very low	Turkish study 1976 to 2010 all pts had radioiodine treatment, could only identify records on 50 out of 61 pts. High incidence of iodine deficiency related goitre in Turkey

	(Chiu et al., 2012)			78	High -	UTC	ns	UTC	ns	Very low	Risk of cancer with thyroid nodule size
Solitary or dominant nodule in multinodular goitre?	(Machens et al., 2010)										
Diffuse or simple multinodular Goitre	(Shayota et al., 2013)	Cohort study		895 SEER 1973-2009	ns	ns	ns	ns	ns	Low	Prognostic factor MeSS

What is the impact of preoperative staging on prognosis?	(Wada et al., 2009)	Retrospective, observational		53	s	ns	ns	s	ns	Very low	Risk factors male, T3/4 and Lns should be considered for aggressive treatment
	(Wada et al., 2009)<br><br>(Vaisman et al., 2011)	Retrospective, observational<br><br>BRAZIL 2 centers, retrospective cohort study		53<br><br>65 children <20yrs, 1980-2005, all cancers	s<br><br>s	ns<br><br>ns	ns<br><br>ns	s<br><br>ns	ns<br><br>ns	Very low<br><br>Very low	multifocality seems not be a predictive factor

	(Powers et al., 2004)	Washington, retrospective , observational, tumour database		Washington, retrospective , observational, tumour database	ns	ns	ns	s	ns	Very low	
	(Scholz et al., 2011)	Retrospective, observational		175 children but only 36% cancer	s	ns	ns	ns	ns	Very low	Recommends FNAC

	(Canadian Pediatric Thyroid Nodule Study, 2008)	Retrospective observational		141 children (DTC and MEN2)	ns	ns	ns	ns	ns	Low	The risk of malignancy in nodule is high (43%) underused FNAC (60%) but recommended
	(Shayota et al., 2013)	Cohort study		895 SEER 1973-2009	ns	ns	ns	ns	ns	Low	Prognostic factor MeSS

What are the prognostic factors and which staging classifications should be used?	(Wada et al., 2009)	Retrospective, observational		53	s	ns	ns	s	ns	Very low	Risk factors male, T3/4 and Lns should be considered for aggressive treatment multifocality seems not be a predictive factor
	(Vaisman et al., 2011)	BRAZIL 2 centres, retrospective cohort study		65 children <20yrs, 1980-2005, all cancers	s	ns	ns	ns	ns	Very low	
	(Powers et al., 2004)	Washington, retrospective, observational, tumour database		Washington, retrospective , observational, tumour database	ns	ns	ns	s	ns	Very low	

	(Al-Qahtani et al., 2015)	Retrospective cohort		27 <18 yrs	s	ns	ns	ns	ns	low	Post-thyroidectomy complications and RAI induced toxicities were observed more in children than adults (P = 0.043 and P = 0.041 respectively). LRR occurred in 6 (22.2 %), 9 (11.5 %) and 3 (5.8 %) in age groups of <18 years, 19–25 years and 26–30 years respectively (P = 0.032); while DM was seen in 10 (37.0 %), 9 (10.3 %) and 5 (9.6 %) in age groups of <18 years, 19–25 years and 26–30 years respectively (P = 0.002). Ten year DFS rates were 67.3 % in age group below 18 years, 82.4 % in age group of 19–25 years and 90.1 % in age group of 26–30 years (P = 0.021).
Who should perform thyroidectomy for cancer in children?<br>Who should perform lymph node dissection in children?<br>Who should perform surgery in cases of advanced/locally invasive thyroid cancer in children?	(Burke et al., 2012)	observational 1 institution (Wisconsin)		74 children < 19 yrs	s	ns	s	ns	s	Very low	Only 24 children had cancer

	(Sosa et al., 2008)	retrospective		1119 < 17 yrs	ns	ns	ns	ns	ns	Low	Recommends high volume surgeons and high volume centres
	(Tuggle et al., 2008)	retrospective		607	ns	ns	ns	ns	ns	Low	High volume surgeons should work with paediatric surgeons<br>high volume predicts LOS and cost
	(Wood et al., 2011)	Observational, retrospective single Institution		35	s	ns	s	s	ns	Very low	
	(Astl et al., 2014)	Retrospective cohort		148 <18 yrs	ns	ns	ns	ns	ns	low	Supports the idea of a total thyroidectomy with selective neck dissection in the treatment of metastases of WDTC

	(Breuer et al., 2013)	Review			-	-	-	-	-	-	-
	(Sosa et al., 2008)			1119 < 17 yrs	ns	ns	ns	ns	ns	Low	
Which complications should be discussed (bleeding, infection, RLN injury, hypoparathyroidism)? What is the incidence of complications of thyroid surgery in children?	(Morris et al., 2012)	Retrospective, 2001-2011 - Texas MD Anderson 2012		74 children < 18 yrs had TT for DTC, MTC and benign	ns	ns	ns	ns	ns	Low	41% non-adherence and 30 hypothyroid - 30% parathyroid dysfunction in 1st year and permanent 8%

	(Burke et al., 2012)			74 children < 19 yrs	s	ns	s	ns	s	Very low	
	(van Santen et al., 2004)			103 children diagnosed between 1989-1997)	ns	ns	ns	ns	s	Very low	84% had complications<br>24% RLN permanent<br>32% permanent hypoPT<br>tracheostomy 8%<br>Horners 8%
	(Lang et al., 2014)	Prospective single center		307	ns	ns	s	ns	ns	Low	Age range of patients: median 59.2 (7.3–90.9) years

	(Machens et al., 2016)	Prospective outcome		62 <18 yrs	s	ns	ns	s	ns	Very low	Transient recurrent laryngeal nerve palsy was significantly associated only with central node dissection (100% vs 55%; P = .010).<br>Transient and permanent hypoparathyroidism were significantly associated with age (means of 11.9 years versus 7.8 years, and 12.9 years versus 8.5 years; P ≤ .002); central node dissection (80% vs 50%, and 100% vs 54%; P ≤ .001); and the number of central lymph nodes cleared (means of 12.2 nodes versus 5.4 nodes, and 26.9 nodes versus 5.8 nodes, P < .001).
	(Schneider et al., 2018)	Retrospective cohort study		504	ns	ns	ns	ns	ns	Low	
What information should be described in the operating notes ?											
What constitutes diagnostic surgery?											

What are the criteria for hemi or total thyroidectomy?	(Newman et al., 1998)	Observational Children Cancer Group		329 children < 21yrs, median 15.2,	ns	ns	ns	ns	ns	Low	Disease progression as the end point<br>Progression free survival (overall 67%) was worse in younger children and residual neck disease<br>Size, Lns, mets, type of surgery, I131 and thyroid radiation did not correlate with PFS<br>8 deaths 2 disease related
	(Massimino et al., 2006)	observational		1968-2001 42 children < 18yrs	ns	vs	ns	ns	ns	Very low	These results contradict other studies, small numbers in each group, and short FU 71 months
	(Raval et al., 2010)	retrospective observational		8013 children registered with cancer) <21yrs 5933 children had TT, 1015 ST, 1065 lobectomy	ns	s	ns	ns	ns	Very low	Use of TT increased from 50.6% in 1985 to 84% in 2007<br>hospital factor TT more likely to be performed in high volume centre<br>tumour factors (size Lns)

	(Wada et al., 2009)			53	s	ns	ns	s	ns	Very low	
	(Spinelli et al., 2016)	Review paper			-	-	-	-	-		
	(Enomoto et al., 2013)	Retrospective analysis		20	s	ns	ns	s	ns	Very low	The average age was 17.3 ± 2.7 years (median, 18 years; range, 11–20 years).

	(Spinelli et al., 2019)	Retrospective analysis 2000-2017	30	30	s	ns	ns	ns	ns	Low	Age <18yrs
	(Jin et al., 2015)	systematic review PTC only		n/a	ns	ns	ns	ns	ns	low	No clear advantage in survival or recurrence rate was found for total thyroidectomy compared to other surgical approaches.
	(Palmer et al., 2005)	retrospective observational		36 children <18yrs	ns	s	ns	ns	ns	Very low	Lns (69%) and multiple thyroid nodules are best predictors of recurrent disease but not survival<br>no distal metastases<br>no death<br>repeated operations required as recurrence rate high (47% as compared to adults 5-20%)<br>paradoxically ST had lower recurrence rates than TT but this must be bias , case selection

	(Haveman et al., 2003)			21 children <18 with thyroid cancer	germ line mutation	?other clinical markers	ns	ns	ns	Low	Recommends TT, neck dissection and RAI
	(Nice et al., 2015)	Retrospective		3861 <21 yrs	ns	ns	ns	s	ns	low	p-PTC >1cm has an excellent 15-year overall survival. Treatment with TT did not have an impact<br><br>3861 with primary papillary thyroid cancer >1cm were reviewed from the National Cancer Data Base (NCDB) from 1998 to 2011proved OS compared to PT
	(Savio et al., 2005)	Retrospective observational		14 children < 17yrs	ns	ns	ns	s	s	Very low	All had TT, 12(86%) LND

	(Jiang et al., 2016)	Retrospective cohort		79	s	ns	ns	ns	ns	low	Average age was 14.5 years with a female: male ratio of 4:1 for WDTC versus 1.6:1 for benign nodules. Neck metastasis was noted in 40 % and pulmonary spread in 10 % of patients.
	(Haugen et al., 2016)	Adult guidelines			-	-	-	-	-	Moderate	
	(Bignol-Kologu et al., 2000)	Retrospective observational		18	s	ns	ns	s	ns	Very low	
	(Hanba et al., 2017)	Retrospective national database review 2009-2012		1099 partial and 1654 total thyroidectomies	s	ns	ns	s	ns	Very low	0-20y

	(Collini et al., 2006)	Retrospective observational		42	s	ns	ns	s	ns	Very low	
	(Dinauer et al., 2008)	Review			-	-	-	-	-	-	
	(Hogan et al., 2009)	Retro cohort (SEER)	1753		s	ns	ns	s	ns	Low	
	(Jarzab et al., 2005)	Review			-	-	-	-	-		

	(Rivkees et al., 2011)	Review			-	-	-	-	-		
	(Welch et al., 1999)	Retrospective cohort	37		s	ns	ns	s	ns	Very low	
	(Spinelli et al., 2004)	Retrospective observational		56 <16yrs	ns	ns	ns	ns	ns	Very low	Recurrence 33.5% (64% radiation, 3% non-radiation) Italian and Belrus population
	(de Jong et al., 2020)	Retrospective cohort		72	ns	ns	ns	ns	ns	Low	

What is the recommended extent of surgery in children with familial or high risk (post-radiation) DTC?	(Kim et al., 2017)	Retrospective comparative 1998-2012	12	1075	s	ns	ns	ns	ns	Low	Age < 18 yrs
What is the role and extent of therapeutic LND to be recommended?	(Spinelli et al., 2018)	Multicentric retrospective analysis	132		s	ns	ns	ns	ns	Low	Age < 18 yrs
	(Handkiewicz-Junak et al., 2007)	observational		235	ns	ns	ns	ns	ns	Low-moderate	86% recurrence free<br>14% of children had local recurrence (TBR 28%, LNR 63%, both 9%)<br>Age not related to recurrence<br>NO DEATHS<br><br>no RAI, less than TT increased risk of TBR recurrence 11x, 9.5x<br>LN recurrence lack of LND 3.3x, lack RAI.3.3, papillary 1.9
	(Ben Arush, 2000)	Retrospective observational	16		s	ns	s	ns	ns	Very low	

	(Chung et al., 2009)	Retrospective observational	245		ns	ns	ns	ns	ns	Low	
	(Popovtzer et al., 2006)	Retrospective cohort	75		ns	ns	ns	s	ns	Very low	
	(Samuels et al., 2018)	Retrospective cohort	18		ns	ns	ns	s	ns	Low-moderate	

	(Baumgarten et al., 2019)	Retrospective observational, single centre, all thyroid sx cases Jan 2009 to Dec 2017		464 - 33% benign nodules,36 % papillary/follicular ca, 27% Graves, 3% medullary ca, 1.5%thyroidectomy for MEN2a	s	ns	s	s	ns	Very low	Mean age 15 years, range 2-24<br><br>6% returned to theatre for haematoma, 11 had vocal cord paresis, 37% had transient hypoparathyroidism, 2% permanent
What is the role of laryngoscopy?	(Lo et al., 2000)	Prospective, single centre Jan 1995-April 1998		501 (84 M 416 f)	s	ns	s	ns	ns	Very low	12-88 (median 43)
	(Heikkinen et al., 2019)	Prospective, single centre 2011-2016		866	s	ns	s	ns	ns	Very low	55 +/- 15

	(Bergenfelz et al., 2008)	Retrospective Scandinavian database review 2004-2006		3660	s	ns	s	ns	ns	Very low	
	(Chen et al., 2015)	Retrospective single centre 1991-2013	13	171	s	ns	ns	ns	ns	Low	<18y mean 15.4y
	(Chandrasekhar et al., 2013)	Adult guideline			ns	ns	ns	s	ns	Moderate	
	(Sinclair et al., 2016)	Consensus statement			vs	s	s	n/a	ns	Very low	

	(Joliat et al., 2017)	Retrospective single centre 2005-2013, single surgeon		648	s	ns	s	ns	ns	Very low	50 (43-63) for thyroidectomies and 63 (48-73) for parathyroidectomies<br><br>7% RLN injuries
	(Gambardella et al., 2020)	prospective multicentric study		396	s	ns	s	ns	ns	Very low	Not in paediatric population

Appendix 5 Consensus Statements and Results

Round 1

The following 30 statements were circulated to a list of 47 multi-professional experts and 16 responses were received. Respondents included consultants in Clinical / Cancer Genetics (2), Endocrinology (5), Paediatric Endocrinology (3), Endocrine Surgery (3), Radiology (1).

Respondents were asked to choose between the following choices for each statement.

I support the statement

I would support the statement with modification

I do not support the statement

I do not have the experience in this area to be able to comment

If you have answered 'I would support the statement with modification' or 'I do not support the statement' please add comments below :

Results:

1. **CYP with thyromegaly (cyst / nodule / diffuse or multinodular goitre) require assessment in an age-appropriate tertiary centre that can undertake all necessary diagnostic investigations including ultrasound, fine needle aspiration (FNA) and thyroidectomy.**

Answer Choices	Responses	
I support the statement	**9**	**50%**
I would support the statement with modification	**5**	**31.25%**
I do not support the statement	**1**	**6.25%**
I do not have the experience in this area to be able to comment	**2**	**12.5%**
**Total**	**16**	

Comments:

How much investigation would be needed at primary or secondary care level before referring to a tertiary centre?

For young children I am comfortable but for more mature children, adult teams must also be involved

I agree that they must be followed in a referral center for thyroid cancer management but not necessarily "age appropriate".

It can be a centre in which adults are followed. The most important is that the disease should be well known.

Please clarify 'age-appropriate tertiary centre'...

Not neccesarily tertiary center

Omit tertiary. Experience more important than kudos.

2. **For all CYP with suspected or proven DTC, care should be provided in an age appropriate tertiary setting, (linked to a paediatric or teenage and young adult (TYA) CCLG oncology centre), and led by a designated endocrinologist or surgeon with expertise in DTC in conjunction with the oncology team.**

Answer Choices	Responses	
I support the statement	**8**	**50%**
I would support the statement with modification	**2**	**12.5%**
I do not support the statement	**0**	
I do not have the experience in this area to be able to comment	**5**	**6.25%**
**Total**	**15**	

Comments:

I support with modification but the tick will not stay in that option. How are you defining suspected or proven DTC - ultrasound or cytology or serum calcitonin? If ultrasound or cytology, then that should have been done by experienced operators.

The cases must involve the adult teams who treat DTC. Surgery and administration may of RAI for under 13s should be in paediatric setting, for older children determine after local discussion

Differentiated Thyroid Cancer (DTC) Guideline Consensus Survey

The childhood thyroid cancer must be followed by endocrinologists and not by oncologists. Children must be followed not only for the cancer but also for their growth since they are or will be operated on and the therapy with LT4 must be managed by an endocrinologist. The type of cancer and the therapeutic procedures do not need the intervention of oncologists.

Or designated oncologist

Conjunction with a team that includes paediatric endocrinology, surgery, nuclear medicine, oncology, and behavioural health who all have experience in managing DTC.

3. **The investigation, treatment (including decisions about the timing and extent of surgical intervention) and pathology of a CYP with suspected or proven DTC should be reviewed at the appropriately constituted adult thyroid MDT.**

Answer Choices	Responses	
I support the statement	**12**	**75%**
I would support the statement with modification	**1**	**6.25%**
I do not support the statement	**2**	**12.5**
I do not have the experience in this area to be able to comment	**1**	**6.25**
**Total**	**16**	

Comments:

Essential

It needs to be an MDT with expertise in thyroid cancer--not all experts in adult thyroid cancer may appreciate the differences in the CYP group, especially children < age 10.

Depends on expertise in child thyroid cancer

I do support the concept of a MDT to evaluate decisions but believe this should be a team of paediatric experts or an adult team in consultation with paediatric experts.

4. **To achieve the best surgical outcomes, the thyroid surgeon from the adult thyroid MDT**

nominated to operate on CYP, should lead the designated surgical team.

Answer Choices	Responses	
I support the statement	**13**	**81.25%**
I would support the statement with modification	**1**	**6.25%**
I do not support the statement	**2**	**12.5%**
I do not have the experience in this area to be able to comment	**0**	**0%**
**Total**	**16**	

Comments:

It depends on local circumstances

In general, the best thyroid cancer surgeons will be "adult"-focused in their practice

Experience in head and neck surgery in children most important

Some endocrine surgeons at tertiary care centers have extensive experience with children and would be better equipped to do this than an adult surgeon. Perhaps the surgeon with the most experience rather than a specific designation would be better here.

5. **To inform and improve future management, CYP with DTC should be registered nationally into a lifelong national outcomes database.**

Answer Choices	Responses	
I support the statement	**13**	**86.7%**
I would support the statement with modification	**1**	**6.67%**
I do not support the statement		
I do not have the experience in this area to be able to comment	**1**	**6.67%**
**Total**	**15**	

Comments:

We cannot advise to use a database that is yet to be created. The statement should mention the need to register them and the need to create the appropriate mechanism to collect accurate data on such patients There are already internet-based registries for endocrine tumours that could be used? (e.g. Eurocrine)

6. **Thyroid antibody levels should be measured in all CYP with thyroid abnormalitie**s

Answer Choices	Responses	
I support the statement	**8**	**53.33%**
I would support the statement with modification	**0**	**23.1%**
I do not support the statement	**3**	**20.0%**
I do not have the experience in this area to be able to comment	**4**	**26.67%**
**Total**	**15**	

Comments:

I do not know what you mean This is a vague and incorrect question

Which thyroid antibody? Why all thyroid abnormalities? Is there evidence to support measurement in euthyroid CYP with nodule(s)?

I am not clear to what this refers. Does this include congenital hypothyroidism for example??

7. **Routine calcitonin measurement in CYP with thyroid abnormalities are not indicated**

Answer Choices	Responses	
I support the statement	**7**	**47.3%**
I would support the statement with modification	**3**	**18.75%**
I do not support the statement	**1**	**6.25%**
I do not have the experience in this area to be able to comment	**5**	**31.25%**
**Total**	**16**	

Comments:

It should be done if there is clinical suspicion from FNA or biopsy, otherwise not

Calcitonin should be measured whenever a thyroid nodule is present. Medullary thyroid cancer is rare in children but there are hereditary forms and in many cases the familial history is silent.

I believe this would require at least a comment that more studies are definitely required

In patients without the appropriate family history...

But is indicated for pedigrees with MTC.

8. **Routine molecular genetic pathology testing (DNA/RNA tests looking for specific tumour mutations) is not required in the assessment of cytology samples of CYP's thyroid disease.**

Answer Choices	Responses	
I support the statement	**10**	**62.5%**
I would support the statement with modification	**4**	**25.0 %**
I do not support the statement	**0**	**0%**
I do not have the experience in this area to be able to comment	**2**	**12.5%**
**Total**	**16**	

Comments:

By "routine", I am assuming you mean in all cases regardless of pathology findings.

It is not yet standard of care but is desirable 10/2/2016 10:25 AM

I suggest a change similar to: At this point genetic testing are not established in clinic practice but multi centre studies are already recruiting patients who will have such tests done in parallel with cytological diagnosis and is likely that within few years the results of such studies will be incorporated in routine clinical practice

This would be true for most cases.

Except for Bethesda III-V

However, future research in this area may lead to more widespread use of molecular studies. If performed, BRAF mutations appear specific to DTC.

9. **Routine molecular genetic pathology testing (DNA/RNA tests looking for specific tumour mutations) is not required in the assessment of histopathology samples of children's and young people's (CYP) thyroid disease.**

Answer Choices	Responses	
I support the statement	**9**	**56.25%**
I would support the statement with modification	**4**	**25.0%**
I do not support the statement	**1**	**6.25%**
I do not have the experience in this area to be able to comment	**2**	**12.5%**
**Total**	**16**	

Comments:

I support the statement but the tick will not stay in that option. By "routine", I am assuming you mean in all cases regardless of pathology findings.

It is desirable but not yet standard of care

It is not required routinely but it would be useful for future assessments

10. **In CYP with thyroid nodules, diagnostic surgery (hemi-thyroidectomy) is preferable to core biopsy if FNA is inconclusive.**

Answer Choices	Responses	
I support the statement	**8**	**50.0%**
I would support the statement with modification	**2**	**12.5%**
I do not support the statement	**4**	**25.0%**
I do not have the experience in this area to be able to comment	**2**	**12.5%**
**Total**	**16**	

Comments:

The reason or the FNA being inconclusive would need to be considered. A repeat FNA may be the more appropriate next investigation.

Probably, you cannot make absolute statements

Depends on US appearance. In non-suspicious lesions, would first repeat FNA after a few months. If suspicious, I would still try to clarify the diagnosis pre-operatively with a repeat biopsy sooner.

I would suggest that repeat FNA is considered, but agree that diagnostic surgery is preferable

11. **The Thy 1-5 FNA classification should be used in CYP who have undergone a thyroid biopsy.**

Answer Choices	Responses	
I support the statement	**12**	**75.0%**
I would support the statement with modification	**1**	**6.25%**
I do not support the statement	**1**	**6.25%**
I do not have the experience in this area to be able to comment	**2**	**12.5%**
**Total**	**16**	

Comments:

By "biopsy", do you mean cytology/FNA or core biopsy? For cytology/FNA - the RCPath Thy1-5 categories, or any subsequent modifications from RCPath, should be used (= I support the statement). For core biopsies - there is not yet a national system for categorising reports. (Personally, I allocate an "equivalent" Thy category after my prose report).

Do you mean an FNA or biopsy Poorly written statement For FNA yes, for biopsy inappropriate

Ultrasound U 1-5 classification should also be used.

12. **CYP with multi-nodular goitre require further investigation for possible DTC: a) even in the absence of other risk factors for DTC b) if there is a family history of thyroid cancer**

Answer Choices	Responses	
I support the statement	**9**	**56.25%**
I would support the statement with modification	**3**	**18.75%**
I do not support the statement	**1**	**6.25%**
I do not have the experience in this area to be able to comment	**3**	**18.75%**
**Total**	**16**	

Comments:

Need to explain the "further investigation" please.

Too many things included with this statement. Confusing as written.

Should give age specific advice

13. **CYP with proven DTC and no prior history of ionizing radiation exposure (therapeutic or environmental) require assessment by a geneticist for an underlying genetic predisposition.**

Answer Choices	Responses	
I support the statement	**5**	**31.25%**
I would support the statement with modification	**2**	**12.5%**
I do not support the statement	**5**	**31.25%**
I do not have the experience in this area to be able to comment	**4**	**25.0%**
**Total**	**16**	

Comments:

I don't think that the genetics are well enough defined for the results to be clinically useful (ie benefit individual patients) at present and I think, therefore, that genetic assessment should be undertaken as part of a study to try to define the implications for individuals or specific groups. Routine testing, in conjunction with a national registry, may lead to developments which will be clinically useful in the future

Not if isolated case

If we advise ALL such children to have genetic testing, how many centres in the country offer this test? I would say "... can be considered for genetic testing..."

Likely not cost effective approach, unless there is a compelling family history or the patient has other findings suggestive of an underlying syndrome.

I don't think this is currently standard practice in our setting.

14. **In CYP with confirmed DTC, if CT is performed prior to surgery iodinated contrast should not be used (given it can delay treatment with radioiodine).**

Answer Choices	Responses	
I support the statement	**7**	**43.75%**
I would support the statement with modification	**1**	**6.25%**
I do not support the statement	**5**	**31.25%**
I do not have the experience in this area to be able to comment	**3**	**18.75%**
**Total**	**16**	

Comments:

Yes a generalisation

CT before surgery is usually not necessary, but, if performed should be done with contrast otherwise the information will not be trustable.

Contrast is absolutely needed for proper imaging with CT. A brief delay in RAI will not change long-term outcomes. 9
MR preferable to CT If CT is chosen, contrast may be given
I would suggest that this statement be modified to 'avoided if possible' as the delay is not significant and does not impact on prognosis.
Usual RAI is given about 6 weeks post-op. After CT the iodine might be cleared in 6-8 weeks. Would prefer to say that urine iodine should be monitored and patient placed on low iodine diet once iodine excretion is low normal. RAI can be given after 2 weeks of low iodine diet.
There is usually sufficient time between imaging and radio-iodine therapy for wash-out.

15. CYP undergoing thyroid surgery should have pre and post operative assessments of vocal cord mobility.

Answer Choices	Responses	
I support the statement	**7**	**43.75%**
I would support the statement with modification	**2**	**12.5%**
I do not support the statement	**1**	**6.25%**
I do not have the experience in this area to be able to comment	**6**	**37.5%**
**Total**	**16**	

Comments:
In an ideal world this would be the case, however in younger children with no voice change, given that they will require long term follow up, the risk of creating distress to examine the vocal cords and longer term hospital phobia, may outweigh the benefits
I don't think it is reasonable to submit a 5-10 years old with normal voice to routine pre and post op laryngoscopy. Might be reasonable in older children (as more patients with DTC are likely to be) The wording makes it 'compulsory' and is unlikely to be adopted in clinical practice
This should be at the discretion of the clinician's judgement. The pick up rate of vocal cord palsy is probably very low in the absence of symptoms. Laryngoscopy can be distressing in children. It may help in some suspected or proven cancer patients, but routine assessments should not be mandated.

16. In CYP undergoing thyroid surgery all operating notes should include: a. information about dissection and preservation of the recurrent laryngeal nerve b. information about number of parathyroid glands identified, preserved and autografted

Answer Choices	Responses	
I support the statement	**12**	**75.0%**
I would support the statement with modification	**0**	**0%**
I do not support the statement	**0**	**0%**
I do not have the experience in this area to be able to comment	**4**	**25%**
**Total**	**16**	

Comments:
None

17. Surveillance frequency of CYP with permanent hypoparathyroidism with biochemical plasma and urine calcium monitoring should be annual once levels are stable.

Answer Choices	Responses	
I support the statement	**9**	**56.25 %**
I would support the statement with modification	**2**	**12.5%**
I do not support the statement	**1**	**6.25%**
I do not have the experience in this area to be able to comment	**4**	**25.0%**
**Total**	**16**	

Comments:

None

18. CYP with permanent hypoparathyroidism require assessment of potential nephrocalcinosis by renal ultrasound at least annually.

Answer Choices	Responses	
I support the statement	**7**	**43.75%**
I would support the statement with modification	**0**	**0%**
I do not support the statement	**4**	**25.0%**
I do not have the experience in this area to be able to comment	**5**	**31.25%**
**Total**	**16**	

Comments:

I would recommend at least 6 monthly monitoring of plasma and urine calcium levels whilst the CYP is still growing; requirements can change dramatically during puberty.

A 24 hr urine calcium might be cheaper and may correlate better with risk of nephrocalcinosis.

Probably annually is too frequent

Is there evidence that this will change management of hypoparathyroidism? I think that urinary calcium measurements should be adequate and screening by ultrasound has no prognostic or therapeutic value - will only add to burden on anxiety and worry.

Question 17 requires more frequent monitoring especially in young growing children.

US not specific enough

19. CYP with permanent hypoparathyroidism require surveillance monitoring of bone densitometry (by DEXA) at least every three years.

Answer Choices	Responses	
I support the statement	**4**	**25.0%**
I would support the statement with modification	**2**	**12.5%**
I do not support the statement	**5**	**31.25%**
I do not have the experience in this area to be able to comment	**5**	**31.25%**
**Total**	**16**	

Comments:

This is not something we routinely do in CYP with hypoparathyroidism due to other causes so do not see why this population should be different.

Probably accurate

Not sure that hypopara has been linked with adverse DXA outcomes.

Compared with age related controls

These patients are not at increased risk of osteoporosis!

Not sure that it needs to be this frequent and may depend on other risk factors

20. Prior to radioiodine treatment in CYP with DTC, low iodine diets should be advised.

Answer Choices	Responses	
I support the statement	**8**	**50.0%**
I would support the statement with modification	**2**	**12.5%**
I do not support the statement	**0**	**0%**
I do not have the experience in this area to be able to comment	**6**	**37.5%**
**Total**	**16**	

Comments:

It is very much dependent from the living area: in Italy the low levels of iodine do not require that patients follow any iodine-low diet.

Although little data to support this!

It depends on the risk stratification and whether or not rTSH could be used instead

21. **The activity of radioactive iodine administered for thyroid ablation should be fixed and irrespective of the size of the child or young person.**

Answer Choices	Responses	
I support the statement	**2**	**12.5%**
I would support the statement with modification	**0**	**0%**
I do not support the statement	**7**	**43.75%**
I do not have the experience in this area to be able to comment	**7**	**43.75%**
**Total**	**16**	

Comments:

The age and the weight of the child must be considered.

Dose of RAI needs to be adjusted based upon patient age, weight, extent of disease, stimulated thyroglobulin, and uptake on a diagnostic scan.

Should be calculated by weight

Dose should be size appropriate

22. **In CYP with low risk DTC, recombinant human TSH administration should be considered (as an off-licence indication) rather than thyroid hormone withdrawal before radioiodine ablation.**

Answer Choices	Responses	
I support the statement	**7**	**43.75%**
I would support the statement with modification	**1**	**6.25%**
I do not support the statement	**3**	**18.75%**
I do not have the experience in this area to be able to comment	**5**	**31.25%**
**Total**	**16**	

Comments:

What would be the additional benefit of this? Thyroid hormone withdrawal is only for a few weeks, so long-term sequelae are extremely unlikely.

Children do quite well after 2 weeks thyroid hormone withdrawal. A commercial product such as rhTSH should not formally recommended in any guidelines unless there is clear evidence to support its superiority in clinical practice.

Could be considered if there are special reasons to avoid hypothyroidism. The majority of children will have an adequate rise in TSH after 2 weeks of thyroid withdrawal.

23. **In CYP requiring radiodine ablation, it should be commenced within one month of surgery (total thyroidectomy or neck dissection for recurrence).**

Answer Choices	Responses

I support the statement	**1**	**6.25%**
I would support the statement with modification	**4**	**25.0%**
I do not support the statement	**7**	**43.75%**
I do not have the experience in this area to be able to comment	**4**	**25.0%**
**Total**	**16**	

Comments:

RIA should be delayed if iodinated contrast has been administered within the preceding 8 weeks. 6 weeks may be a more realistic target and allows time for post-op healing where surgery has been complicated by wound infection

Three months are also ok

There is no proof that delaying by few months impacts on the outcome. If parents would read such a statement will create panic if RIA is not received with 4 weeks postop In my view it could be delayed even for convenience (e.g.doing during holiday period to avoid the problems with school/peers contact, etc

RAI can be safely deferred for several months. Does not have to be done right away.

Or 2 months if ct contrast given 9/16/2016 4:24 PM

I think this point needs individualization in many respects - again perhaps depending on whether or not rTSH can be used to avoid the growth inhibition of hypothyroidism

There is no evidence that this is necessary. Given that it is often necessary to refer to a centre with paediatric nuclear medicine facilities this is almost certainly not feasible. Adult guidelines suggest treatment within 6 months is appropriate.

I would typically do this 6-8 weeks out and there is no rush to treat. Post-op staging should be done to evaluate need for RAI prior to giving any and that will require a few weeks. Post-op staging should include US of neck, suppressed Tg or TSH_stimulated Tg in higher risk patients and possibly RAI whole body scan.

24. Peri-pubertal boys and young adult males likely to require more than two administrations of radioactive iodine (whether therapy or ablation) should be offered the opportunity of cryopreserving sperm.

**Answer Choices**	**Responses**	
I support the statement	**5**	**31.25%**
I would support the statement with modification	**2**	**12.5%**
I do not support the statement	**4**	**25.0%**
I do not have the experience in this area to be able to comment	**5**	**31.25%**
**Total**	**16**	

Comments:

I am unaware of any good evidence that radio-iodine treatment causes male infertility. 10/10/2016 There are no evidences that one or two courses of 131-I can affect the spermatogenesis in "normal" subject. What we should do is to verify if there are problems with spermatogenesis "before" the treatment. The cryopreservation should be offered to those who are already oligospermic.

I don't have experience with this but I did not come across any papers describing infertility after RIA in teenagers 9

Not sure this is necessary in the vast majority of cases, especially given current practiced of not administering extremely high RAI doses frequently

Should be discussed

25. In CYP, external beam radiotherapy has no place in the treatment of potentially

curable DTC, but the MDT may consider its use in the palliation of extensive inoperable / incurable disease in individual cases.

Answer Choices	Responses	
I support the statement	**7**	**43.75%**
I would support the statement with modification	**1**	**6.25%**
I do not support the statement	**3**	**18.75%**
I do not have the experience in this area to be able to comment	**5**	**31.25%**
**Total**	**16**	

Comments:

External radiotherapy in children with DTC is not indicated

Radiotherapy should only be reserved for patients with ATC. Almost all cases of DTC in the CYP group are operable by a high volume surgeon and one would not want radiation given to a patient with residual disease only because he/she did not meet the correct surgical team.

In the rare case when surgery and RAI have failed and if TKI therapy is not possible.

26. **The safety and benefits of relaxing long term TSH suppression in CYP with DTC stratified as low risk by rhTSH/ THW stimulation adult protocols requires national study. In the meantime CYP with DTC should receive TSH suppression to adulthood and then undergo dynamic restratification.**

Answer Choices	Responses	
I support the statement	**8**	**50.0%**
I would support the statement with modification	**1**	**6.25%**
I do not support the statement	**3**	**18.75%**
I do not have the experience in this area to be able to comment	**4**	**25.0%**
**Total**	**16**	

Comments:

Agree with the first sentence. The second sentence is not supported by evidence. Keeping a low-risk, young patient on overt TSH suppression may likely have more long-term harm than benefit. Also, dynamic re-stratification can be done at almost any time and does not require waiting until adulthood.

This is probably the pragmatic way of doing it - but more studies on this particular point would be needed

I do not see a need to tightly suppress for low risk patients

27. **In CYP with DTC who have completed treatment, a minimum 6 monthly surveillance monitoring with thyroglobulin measurements is recommended until adulthood (> 19yr), followed by monitoring according to adult BTA guidelines.**

Answer Choices	Responses	
I support the statement	**9**	**56.25%**
I would support the statement with modification	**2**	**12.5%**
I do not support the statement	**1**	**6.25%**
I do not have the experience in this area to be able to comment	**4**	**25.0%**
**Total**	**16**	

Comments:

Once a patient is NED for 2-3 years, annual FU can be performed.
3 months

28. During surveillance monitoring of CYP treated for DTC, any single elevated thyroglobulin value requires confirmation in a repeat sample within 2 months before consideration of further investigation and MDT discussion.

Answer Choices	Responses	
I support the statement	**9**	**56.25%**
I would support the statement with modification	**1**	**6.25%**
I do not support the statement	**2**	**12.5%**
I do not have the experience in this area to be able to comment	**4**	**25.0%**
**Total**	**16**	

Comment:

Repeat first

Within 6 months instead of "2 months" would be more reasonable.

Follow trend in Tg not just single values

29. Adult prognostic scoring systems (TNM) aid management decisions in CYP with DTC.

Answer Choices	Responses	
I support the statement	**8**	**50.0%**
I would support the statement with modification	**3**	**18.75%**
I do not support the statement	**1**	**6.25%**
I do not have the experience in this area to be able to comment	**4**	**25.0%**
**Total**	**16**	

Comments:

For pathology TNM, needs to be done accurately, correctly and by an experienced endocrine pathologist.

The TNM system really does not help with survival but may predict patients at highest risk for distant disease and recurrence.

We probably do not know how they work for children

No data to support this

30. A prognostic (MACIS) score should be recorded centrally in any register for CYP with papillary thyroid carcinoma.

Answer Choices	Responses	
I support the statement	**7**	**43.72%**
I would support the statement with modification	**1**	**6.25%**
I do not support the statement	**3**	**18.75%**
I do not have the experience in this area to be able to comment	**5**	**31.25%**
**Total**	**16**	

Comments:

TNM is preferable to MACIS

TNM stratification is probably better than MACIS

Following the first round of the Consensus Questions, the results were collated.

Question number	Number replying	Agreement	Comment
1.	16	14/15	93%
2.	15	10/10	100%
3	16	13/15	87%
4	15	14/16	88%
5	15	14/14	100%
6	16	8/11	73%
7	16	10/11	91%
8	16	14/14	100%
9	16	13/14	93%
10	16	10/14	71%
11	16	13/14	93%
12	16	12/13	93%
13	16	7/12	58%
14	16	8/13	62%
15	16	9/10	90%
16	16	12/12	100%
17	16	11/12	92%
18	16	7/11	64%
19	16	6/11	55%
20	16	10/10	100%
21	16	2/9	22%
22	16	8/11	73%
23	16	5/12	42%
24	16	7/11	64%
25	16	8/11	73%
26	16	9/12	75%
27	16	11/12	92%
28	16	10/12	83%
29	16	11/12	92%
30	16	8/11	73%

Questions 12 was therefore re-issued in the second round in two separate questions (2nd round, questions 1 and 2). Question 26 was rewritten (2nd round, question 3)

Delphi Second Round Questions

1. **CYP with multi-nodular goitre with no other risk factors for DTC requires investigation for possible DTC**

Answer Choices	Responses	
I support the statement	**6**	**55%**
I would support the statement with modification	**4**	**36%**
I do not support the statement	**0**	
I do not have the experience in this area to be able to comment	**1**	**0.9%**
**Total**	**11**	

Comments:

What is meant by "investigation"? - presume ultrasound +/- FNA. I would support that for MNG in CYP.

Depends what is meant by investigation for DTC. If this is a child with clear evidence of thyroid dysfunction and positive thyroid antibodies, what additional investigations are proposed? If this was an ultrasound, would agree. If the proposal was for FNAC, then that is probably a bit OTT.

Subject to identifying key risk factors You do want to see every MNG in a child

2. **CYP with multi-nodular goitre with a family history of thyroid cancer requires investigation for possible DTC**

Answer Choices	Responses	
I support the statement	**8**	**73%**
I would support the statement with modification	**3**	**27%**
I do not support the statement	**0**	
I do not have the experience in this area to be able to comment	**0**	
**Total**	**11**	

Comments:

What is meant by "family history of thyroid cancer" - DTC or MTC? And what is meant by "investigation"? If the family history is of DTC, I would support ultrasound +/- FNA for MNG in CYP. If the family history is MTC, I would support the addition of genetic testing, followed by (if positive) serum calcitonin, calcium assessment and exclusion of phaeochromocytoma.

I would also include a significant family history of other cancers known to associate with DTC. For instance colorectal cancer(polyposis), premenopausal breast cancer(cowden), DICER 1 associated tumours etc.

This is more reasonable but language: should be considered for further investigation

3. **CYP with DTC stratified as low risk by rhTSH/ THW stimulation adult protocols should receive TSH suppression to adulthood**

Answer Choices	Responses	
I support the statement	**3**	**27%**
I would support the statement with modification	**2**	**18%**
I do not support the statement	**3**	**27%**
I do not have the experience in this area to be able to comment	**3**	**27%**
**Total**	**11**	

Comments:

Potential morbidity probably exceeds possible benefit

There is no sufficient evidence that TSH suppression is beneficial for prognosis whereas TSH suppression may have disadvantageous effects on growth/development

They should receive TSH suppression until the evidence of clinical remission

You have not defined level of suppression, I think you should specify a range, say desirable range is 0.5 to 1

Following the second round of the Consensus Questions, the results were collated.

Question number	Number replying	Agreement	Comment
1.	11	10/10	100%
2.	11	10/10	100%
3	11	8/10	80%

The different options for management of DTC were considered by a subgroup of the GDG who are expert in its management. They reviewed the literature and identified the Delphi questions for consideration by the full GDG. The different options for management were considered and discussed in light of the published evidence and the Delphi Consensus results.

Appendix 6: Research Recommendations

Having reviewed the evidence and sought consensus opinion on areas where evidence is contradictory or poor, the GDG would like to make the following recommendations.

1. <u>Frequency of pathogenic variants in children with DTC</u>

The proportion of children with DTC and a pathogenic variant in a differentiated thyroid cancer predisposing gene is unknown. A future recommendation would be to study the proportion of children who underwent a thyroidectomy with a syndromic diagnosis to verify the incidence of syndromic DTC in CYP. This data could be ascertained from National Cancer registries and CCLG.

In addition, CYP with proven DTC, no clear syndromic diagnosis and no prior history of ionizing radiation exposure (therapeutic or environmental) may benefit from assessment by a geneticist and genetic testing as part of future research, in order to determine the proportion of such CYP who have an undiagnosed underlying genetic predisposition.

2. <u>Should all children have pre and post operative direct laryngoscopy to assess vocal cord function?</u>

Limited evidence was identified to support the recommendation of pre and post-operative vocal cord mobility assessment. This was thus reviewed by Delphi consensus: the challenges of such assessments via laryngoscopy in younger children were identified with recommendation that such assessments should be considered especially in CYP with extensive or metastatic disease.

Suggested research recommendation i) to test the validity of assessment of vocal cord mobility pre-and post-operatively by ultrasound versus laryngoscopy in CYP with DTC , and ii) to assess (continuous) intraoperative neuromonitoring in CYP with DTC to preserve vocal cord function.

3. <u>Molecular markers in DTC</u>

Although there is increasing evidence for the use of molecular markers to diagnose malignancy in CYP with thyroid lesions, many of these mutational analyses have not been fully validated in CYP and evidence for diagnostic use is lacking in the paediatric population. Further, there is insufficient data on the negative predictive value of these tests. For future recommendations, it is essential to standardise the molecular methodologies used for testing, and future studies need to focus attention on this question. Differentiating which are RNA or DNA based will impact on sample type, storage and preparation. Future trials should consider inclusion of molecular subtype into risk stratification. Finally, such analysis needs to include a cost effectiveness analysis on the impact of NHS commissioning.

4. <u>Dosimetry in RIT in CYP with DTC</u>

More prospective data on dosimetry in children receiving iodine 131 is critical and a clinical trial should be considered.

5. Long term TSH suppression in CYP with DTC stratified as low risk

The safety and benefits of relaxing long term TSH suppression in CYP with DTC stratified as low risk (by rhTSH or stimulation by thyroid hormone withdrawal via adult protocols) requires national study.

6. Screening of CYP at risk of DTC (due to syndromic features or family history of DTC or related cancers)

Although the risk of childhood DTC development in these syndromes is overall low, cases of DTC in PTEN mutation positive individuals have been documented as young as 6yrs (18) with nodules identified from 5yrs of age (19). Data from the International Cowden Consortium identified an elevated risk of DTC from birth, with approximately 16% of PTEN mutation positive patients who develop thyroid cancer likely to present before the age of 18yrs (21). In children with clinical or genetic diagnosis known to be associated with DTC screening should be undertaken. The age of starting screening and frequency of screening is currently determined by risks posed by the specific syndromes and diagnoses, but further research is required. There is insufficient evidence to recommend screening by ultrasound over clinical assessment with palpation in these patients, and further research is needed to clarify these screening protocols.

7. Define 'high-volume' for members of the medical and surgical components of the MDT

Our guideline, in line with previous guidelines for paediatric DTC, recommends that CYP with DTC should be under the care of a 'high-volume' surgeon, defined by >30 cervical endocrine procedures per year. However, it is not yet clear as to how to define high-volume for other medical and surgical members of the MDT, or what constitutes a high-volume centre, and we propose that metrics for these definitions should be discussed and validated in future research. These discussions could also include which medical specialist is best placed to lead the medical MDT.

References

Policies and procedures | Who we are | About | NICE [Online]. Available: https://www.nice.org.uk/about/who-we-are/policies-and-procedures [Accessed Accessed May 23, 2018].

ABBASIAN ARDAKANI, A., REIAZI, R. & MOHAMMADI, A. 2018. A Clinical Decision Support System Using Ultrasound Textures and Radiologic Features to Distinguish Metastasis From Tumor-Free Cervical Lymph Nodes in Patients With Papillary Thyroid Carcinoma. *J Ultrasound Med,* 37**,** 2527-2535.

AL-QAHTANI, K. H., TUNIO, M. A., AL ASIRI, M., ALJOHANI, N. J., BAYOUMI, Y., RIAZ, K. & ALSHAKWEER, W. 2015. "Clinicopathological features and treatment outcomes of differentiated thyroid cancer in Saudi children and adults". *J Otolaryngol Head Neck Surg,* 44**,** 48.

AL-QURAYSHI, Z., HAUCH, A., SRIVASTAV, S., ASLAM, R., FRIEDLANDER, P., KANDIL, E., PW, G., NL, S., J, E.-C., L, R., ME, G., GB, T., A, Y., CA, D., KD, N., D, Z., C, A., D, R., LG, B., JC, L., SH, B., SG, E., S, S., JA, S., CT, T., TS, W., YE, D., AR, H. & GL, F. 2016. A National Perspective of the Risk, Presentation, and Outcomes of Pediatric Thyroid Cancer. *JAMA Otolaryngology–Head & Neck Surgery,* 142**,** 472.

AL NOFAL, A., GIONFRIDDO, M. R., JAVED, A., HAYDOUR, Q., BRITO, J. P., PROKOP, L. J., PITTOCK, S. T. & MURAD, M. H. 2016. Accuracy of thyroid nodule sonography for the detection of thyroid cancer in children: systematic review and meta-analysis. *Clinical Endocrinology,* 84**,** 423-430.

ALESSANDRI, A. J., GODDARD, K. J., BLAIR, G. K., FRYER, C. J. & SCHULTZ, K. R. 2000. Age is the major determinant of recurrence in pediatric differentiated thyroid carcinoma. *Med Pediatr Oncol,* 35**,** 41-6.

ALTINCIK, A., DEMIR, K., ABACI, A., BOBER, E. & BUYUKGEBIZ, A. 2010. Fine-needle aspiration biopsy in the diagnosis and follow-up of thyroid nodules in childhood. *J Clin Res Pediatr Endocrinol,* 2**,** 78-80.

ANTONELLI, A., MICCOLI, P., FALLAHI, P., GROSSO, M., NESTI, C., SPINELLI, C. & FERRANNINI, E. 2003. Role of neck ultrasonography in the follow-up of children operated on for thyroid papillary cancer. *Thyroid,* 13**,** 479-484.

ARETZ, S., UHLHAAS, S., CASPARI, R., MANGOLD, E., PAGENSTECHER, C., PROPPING, P. & FRIEDL, W. 2004. Frequency and parental origin of de novo APC mutations in familial adenomatous polyposis. *Eur J Hum Genet,* 12**,** 52-8.

ASTL, J., CHOVANEC, M., LUKES, P., KATRA, R., DVORAKOVA, M., VLCEK, P., SYKOROVA, P. & BETKA, J. 2014. Thyroid carcinoma surgery in children and adolescents - 15 years experience surgery of pediatric thyroid carcinoma. *Int J Pediatr Otorhinolaryngol,* 78**,** 990-4.

BAL, C. S., KUMAR, A., CHANDRA, P., DWIVEDI, S. N. & MUKHOPADHYAYA, S. 2004. Is chest x-ray or high-resolution computed tomography scan of the chest sufficient investigation to detect pulmonary metastasis in pediatric differentiated thyroid cancer? *Thyroid,* 14**,** 217-225.

BAL, C. S., PADHY, A. K. & KUMAR, A. 2001. Clinical features of differentiated thyroid carcinoma in children and adolescents from a sub-Himalayan iodine-deficient endemic zone. *Nucl Med Commun,* 22**,** 881-7.

BALACHANDAR, S., LA QUAGLIA, M., TUTTLE, R. M., HELLER, G., GHOSSEIN, R. A. & SKLAR, C. A. 2016. Pediatric Differentiated Thyroid Carcinoma of Follicular Cell Origin: Prognostic Significance of Histologic Subtypes. *Thyroid,* 26**,** 219-26.

BALLESTER, L. Y., SARABIA, S. F., SAYEED, H., PATEL, N., BAALWA, J., ATHANASSAKI, I., HERNANDEZ, J. A., FANG, E., QUINTANILLA, N. M., ROY, A. & LÓPEZ-TERRADA, D. H. 2016. Integrating Molecular Testing in the Diagnosis and Management of Children with Thyroid Lesions. *Pediatric and Developmental Pathology,* 19**,** 94-100.

BARGREN, A. E., MEYER-ROCHOW, G. Y., SYWAK, M. S., DELBRIDGE, L. W., CHEN, H. & SIDHU, S. B. 2010. Diagnostic utility of fine-needle aspiration cytology in pediatric differentiated thyroid cancer. *World J Surg,* 34**,** 1254-60.

BAŞ, V. N., AYCAN, Z., CETINKAYA, S., UNER, C., CAVUŞOĞLU, Y. H. & ARDA, N. 2012. Thyroid nodules in children and adolescents a single institution's experience. *Journal of pediatric endocrinology & metabolism : JPEM,* 25**,** 633.

BAUMGARTEN, H. D., BAUER, A. J., ISAZA, A., MOSTOUFI-MOAB, S., KAZAHAYA, K. & ADZICK, N. S. 2019. Surgical management of pediatric thyroid disease: Complication rates after thyroidectomy at the Children's Hospital of Philadelphia high-volume Pediatric Thyroid Center. *J Pediatr Surg*.

BEIMFOHR, C., KLUGBAUER, S., DEMIDCHIK, E. P., LENGFELDER, E. & RABES, H. M. 1999. NTRK1 RE-ARRANGEMENT IN PAPILLARY THYROID CARCINOMAS OF CHILDREN AFTER THE CHERNOBYL REACTOR ACCIDENT. *International Journal of Pediatric Otorhinolaryngology,* 847**,** 842-847.

BEN ARUSH, M. W., STEIN, M. E., PEREZ NAHUM, M., ZIDAN, J., & KUTEN, A. 2000. Pediatric thyroid carcinoma 22 years of experience at the Northern Israel Oncology Center (1973-1995). *pediatric hematology and oncology,* 17**,** 85-92.

BERGENFELZ, A., JANSSON, S., KRISTOFFERSSON, A., MARTENSSON, H., REIHNER, E., WALLIN, G. & LAUSEN, I. 2008. Complications to thyroid surgery: results as reported in a database from a multicenter audit comprising 3,660 patients. *Langenbecks Arch Surg,* 393**,** 667-73.

BHATTI, P., VEIGA, L. H., RONCKERS, C. M., SIGURDSON, A. J., STOVALL, M., SMITH, S. A., WEATHERS, R., LEISENRING, W., MERTENS, A. C., HAMMOND, S., FRIEDMAN, D. L., NEGLIA, J. P., MEADOWS, A. T., DONALDSON, S. S., SKLAR, C. A., ROBISON, L. L. & INSKIP, P. D. 2010. Risk of second primary thyroid cancer after radiotherapy for a childhood cancer in a large cohort study: an update from the childhood cancer survivor study. *Radiat Res,* 174**,** 741-52.

BIGNOL-KOLOGU, TANYEL, F. C., E, M., BÜYÜKPAMUKC, N. & HIC, A. 2000. Surgical Treatment of Differentiated Thyroid Carcinoma in Children. *European journal of pediatric surgery,* 2000**,** 347-452.

BIKO, J., REINERS, C., KREISSL, M. C., VERBURG, F. A., DEMIDCHIK, Y. & DROZD, V. 2011. Favourable course of disease after incomplete remission on (131)I therapy in children with pulmonary metastases of papillary thyroid carcinoma: 10 years follow-up. *Eur J Nucl Med Mol Imaging,* 38**,** 651-5.

BORSON-CHAZOT, F., CAUSERET, S., LIFANTE, J. C., AUGROS, M., BERGER, N. & PEIX, J. L. 2004. Predictive factors for recurrence from a series of 74 children and adolescents with differentiated thyroid cancer. *World J Surg,* 28**,** 1088-92.

BREUER, C., TUGGLE, C., SOLOMON, D. & SOSA, J. A. 2013. Pediatric thyroid disease: when is surgery necessary, and who should be operating on our children? *J Clin Res Pediatr Endocrinol,* 5 Suppl 1**,** 79-85.

BRIGNARDELLO, E., CORRIAS, A., ISOLATO, G., PALESTINI, N., CORDERO DI MONTEZEMOLO, L., FAGIOLI, F. & BOCCUZZI, G. 2008. Ultrasound screening for thyroid carcinoma in childhood cancer survivors: a case series. *J Clin Endocrinol Metab,* 93**,** 4840-3.

BRINK, J. S., VAN HEERDEN, J. A., MCIVER, B., SALOMAO, D. R., FARLEY, D. R., GRANT, C. S., THOMPSON, G. B., ZIMMERMAN, D. & HAY, I. D. 2000. Papillary thyroid cancer with pulmonary metastases in children: long-term prognosis. *Surgery,* 128**,** 881-6; discussion 886-7.

BROSE, M. S., NUTTING, C. M., JARZAB, B., ELISEI, R., SIENA, S., BASTHOLT, L., DE LA FOUCHARDIERE, C., PACINI, F., PASCHKE, R., SHONG, Y. K., SHERMAN, S. I., SMIT, J. W., CHUNG, J., KAPPELER, C., PENA, C., MOLNAR, I., SCHLUMBERGER, M. J. & INVESTIGATORS, D. 2014. Sorafenib in radioactive iodine-refractory, locally

advanced or metastatic differentiated thyroid cancer: a randomised, double-blind, phase 3 trial. *Lancet,* 384**,** 319-28.

BROSE, M. S., SMIT, J., CAPDEVILA, J., ELISEI, R., NUTTING, C., PITOIA, F., ROBINSON, B., SCHLUMBERGER, M., SHONG, Y. K. & TAKAMI, H. 2012. Regional approaches to the management of patients with advanced, radioactive iodine-refractory differentiated thyroid carcinoma. *Expert Rev Anticancer Ther,* 12**,** 1137-47.

BURKE, J. F., SIPPEL, R. S. & CHEN, H. 2012. Evolution of pediatric thyroid surgery at a tertiary medical center. *J Surg Res,* 177**,** 268-74.

BURYK, M. A., SIMONS, J. P., PICARSIC, J., MONACO, S. E., OZOLEK, J. A., JOYCE, J., GURTUNCA, N., NIKIFOROV, Y. E. & WITCHEL, S. F. 2015. Can Malignant Thyroid Nodules Be Distinguished from Benign Thyroid Nodules in Children and Adolescents by Clinical Characteristics? A Review of 89 Pediatric Patients with Thyroid Nodules. *Thyroid,* 25**,** 392-400.

CANADIAN PEDIATRIC THYROID NODULE STUDY, G. 2008. The Canadian Pediatric Thyroid Nodule Study: an evaluation of current management practices. *J Pediatr Surg,* 43**,** 826-30.

CHANDRASEKHAR, S. S., RANDOLPH, G. W., SEIDMAN, M. D., ROSENFELD, R. M., ANGELOS, P., BARKMEIER-KRAEMER, J., BENNINGER, M. S., BLUMIN, J. H., DENNIS, G., HANKS, J., HAYMART, M. R., KLOOS, R. T., SEALS, B., SCHREIBSTEIN, J. M., THOMAS, M. A., WADDINGTON, C., WARREN, B., ROBERTSON, P. J., AMERICAN ACADEMY OF, O.-H. & NECK, S. 2013. Clinical practice guideline: improving voice outcomes after thyroid surgery. *Otolaryngol Head Neck Surg,* 148**,** S1-37.

CHEN, Y., MASIAKOS, P. T., GAZ, R. D., HODIN, R. A., PARANGI, S., RANDOLPH, G. W., SADOW, P. M. & STEPHEN, A. E. 2015. Pediatric thyroidectomy in a high volume thyroid surgery center: Risk factors for postoperative hypocalcemia. *J Pediatr Surg,* 50**,** 1316-9.

CHIU, H. K., SANDA, S., FECHNER, P. Y. & PIHOKER, C. 2012. Correlation of TSH with the risk of paediatric thyroid carcinoma. *Clin Endocrinol (Oxf),* 77**,** 316-22.

CHOW, S. M., LAW, S. C., MENDENHALL, W. M., AU, S. K., YAU, S., MANG, O. & LAU, W. H. 2004. Differentiated thyroid carcinoma in childhood and adolescence-clinical course and role of radioiodine. *Pediatr Blood Cancer,* 42**,** 176-83.

CHUNG, Y. S., KIM, J. Y., BAE, J. S., SONG, B. J., KIM, J. S., JEON, H. M., JEONG, S. S., KIM, E. K. & PARK, W. C. 2009. Lateral lymph node metastasis in papillary thyroid carcinoma: results of therapeutic lymph node dissection. *Thyroid,* 19**,** 241-6.

CLEMENT, S. C., VAN RIJN, R. R., VAN ECK-SMIT, B. L., VAN TROTSENBURG, A. S., CARON, H. N., TYTGAT, G. A. & VAN SANTEN, H. M. 2015. Long-term efficacy of current thyroid prophylaxis and future perspectives on thyroid protection during 131I-metaiodobenzylguanidine treatment in children with neuroblastoma. *Eur J Nucl Med Mol Imaging,* 42**,** 706-15.

COLE, C. D. & WU, H. H. 2014. Fine-needle aspiration in pediatric patients 12 years of age and younger. *Diagnostic Cytopathology,* 42**,** 600-605.

COLLINI, P., MASSIMINO, M., LEITE, S. F., MATTAVELLI, F., SEREGNI, E., ZUCCHINI, N., SPREAFICO, F., FERRARI, A., CASTELLANI, M. R., CANTU, G., FOSSATI-BELLANI, F., ROSAI, J. & THYROID CANCER STUDY GROUP OF THE ISTITUTO NAZIONALE TUMORI OF MILAN, I. 2006. Papillary thyroid carcinoma of childhood and adolescence: a 30-year experience at the Istituto Nazionale Tumori in Milan. *Pediatr Blood Cancer,* 46**,** 300-6.

CORDIOLI, M. I. C. V., MORAES, L., CARVALHEIRA, G., SISDELLI, L., ALVES, M. T. S., DELCELO, R., MONTE, O., LONGUI, C. A., CURY, A. N. & CERUTTI, J. M. 2016. AGK-BRAF gene fusion is a recurrent event in sporadic pediatric thyroid carcinoma. *Cancer Medicine,* 5**,** 1535-1541.

CORRIAS, A., EINAUDI, S., CHIORBOLI, E., WEBER, G., CRINÒ, A., ANDREO, M., CESARETTI, G., DE SANCTIS, L., MESSINA, M. F., SEGNI, M., CICCHETTI, M., VIGONE, M., PASQUINO, A. M., SPERA, S., DE LUCA, F., MUSSA, G. C. & BONA, G. 2001. Accuracy of Fine Needle Aspiration Biopsy of Thyroid Nodules in Detecting Malignancy in Childhood Comparison with Conventional Clinical, Laboratory, and Imaging Approaches *Journal of Clinical Endocrinology and Metabolism,* 86**,** 4644-4648.

CORRIAS, A. & MUSSA, A. 2013. Thyroid nodules in pediatrics: which ones can be left alone, which ones must be investigated, when and how. *J Clin Res Pediatr Endocrinol,* 5 Suppl 1**,** 57-69.

CORRIAS, A., MUSSA, A., BARONIO, F., ARRIGO, T., SALERNO, M., SEGNI, M., VIGONE, M. C., GASTALDI, R., ZIRILLI, G., TULI, G., BECCARIA, L., IUGHETTI, L., EINAUDI, S., WEBER, G., DE LUCA, F., CASSIO, A., STUDY GROUP FOR THYROID DISEASES OF ITALIAN SOCIETY FOR PEDIATRIC, E. & DIABETOLOGY 2010. Diagnostic features of thyroid nodules in pediatrics. *Arch Pediatr Adolesc Med,* 164**,** 714-9.

DE JONG, M. C., GAZE, M. N., SZYCHOT, E., ROZALEN GARCIA, V., BRAIN, C., DATTANI, M., SPOUDEAS, H., HINDMARSH, P., ABDEL-AZIZ, T. E., BOMANJI, J., SHANKAR, A., STONEHAM, S., MORLEY, S., BEALE, T., JAWAD, S., OTERO, S., PROCTOR, I., AMIN, S., BUTLER, G., HEWITT, R. J. & KURZAWINSKI, T. R. 2020. Treating papillary and follicular thyroid cancer in children and young people: Single UK-center experience between 2003 and 2018. *J Pediatr Surg*.

DE KOCK, L., SABBAGHIAN, N., SOGLIO, D. B., GUILLERMAN, R. P., PARK, B. K., CHAMI, R., DEAL, C. L., PRIEST, J. R. & FOULKES, W. D. 2014. Exploring the association Between DICER1 mutations and differentiated thyroid carcinoma. *J Clin Endocrinol Metab,* 99**,** E1072-7.

DEMIDCHIK, Y. E., DEMIDCHIK, E. P., REINERS, C., BIKO, J., MINE, M., SAENKO, V. A. & YAMASHITA, S. 2006. Comprehensive clinical assessment of 740 cases of surgically treated thyroid cancer in children of Belarus. *Ann Surg,* 243**,** 525-32.

DERMODY, S., WALLS, A. & HARLEY, E. H., JR. 2016. Pediatric thyroid cancer: An update from the SEER database 2007-2012. *Int J Pediatr Otorhinolaryngol,* 89**,** 121-6.

DINAUER, C. A., BREUER, C. & RIVKEES, S. A. 2008. Differentiated thyroid cancer in children: diagnosis and management. *Curr Opin Oncol,* 20**,** 59-65.

DOTTORINI, M. E., VIGNATI, A., MAZZUCCHELLI, L., LOMUSCIO, G., COLOMBO, L. & MEDICINA, U. 1997. Differentiated Thyroid Carcinoma in Children and Adolescents A 37-Year Experience in 85 Patients Year Experience in 85 Patients *J Nucl Med,* 38**,** 669-675.

DURANTE, C., COSTANTE, G. & FILETTI, S. 2013. Differentiated thyroid carcinoma: defining new paradigms for postoperative management. *Endocr Relat Cancer,* 20**,** R141-54.

ENOMOTO, K., ENOMOTO, Y., UCHINO, S., YAMASHITA, H. & NOGUCHI, S. 2013. Follicular thyroid cancer in children and adolescents: clinicopathologic features, long-term survival, and risk factors for recurrence. *Endocr J,* 60**,** 629-35.

ENOMOTO, Y., ENOMOTO, K., UCHINO, S., SHIBUYA, H., WATANABE, S. & NOGUCHI, S. 2012. Clinical features, treatment, and long-term outcome of papillary thyroid cancer in children and adolescents without radiation exposure. *World J Surg,* 36**,** 1241-6.

FALLAH, M., PUKKALA, E., TRYGGVADOTTIR, L., OLSEN, J. H., TRETLI, S., SUNDQUIST, K. & HEMMINKI, K. 2013. Risk of thyroid cancer in first-degree relatives of patients with non-medullary thyroid cancer by histology type and age at diagnosis: a joint study from five Nordic countries. *J Med Genet,* 50**,** 373-82.

FARAHATI, J., PARLOWSKY, T., MÄDER, U., REINERS, C. & BUCSKY, P. 1998. Differentiated thyroid cancer in children and adolescents. *Langenbeck's archives of surgery,* 383**,** 235-6.

FENTON, C. L., LUKES, Y., NICHOLSON, D., DINAUER, C. A., FRANCIS, G. L. & TUTTLE, R. M. 2000. The ret PTC Mutations Are Common in Sporadic Papillary Thyroid Carcinoma of Children and Young Adults. *Journal of Clinical Endocrinology and Metabolism,* 85**,** 1170-1175.

FINKELSTEIN, A., LEVY, G. H., HUI, P., PRASAD, A., VIRK, R., CHHIENG, D. C., CARLING, T., ROMAN, S. A., SOSA, J. A., UDELSMAN, R., THEOHARIS, C. G. & PRASAD, M. L. 2012. Papillary thyroid carcinomas with and without BRAF V600E mutations are morphologically distinct. *Histopathology,* 60**,** 1052-9.

FRANCIS, G. L., WAGUESPACK, S. G., BAUER, A. J., ANGELOS, P., BENVENGA, S., CERUTTI, J. M., DINAUER, C. A., HAMILTON, J., HAY, I. D., LUSTER, M., PARISI, M. T., RACHMIEL, M., THOMPSON, G. B., YAMASHITA, S. & AMERICAN THYROID ASSOCIATION GUIDELINES TASK, F. 2015. Management Guidelines for Children with Thyroid Nodules and Differentiated Thyroid Cancer. *Thyroid,* 25**,** 716-59.

FREIRE, A. V., ROPELATO, M. G., BALLERINI, M. G., ACHA, O., BERGADA, I., DE PAPENDIECK, L. G. & CHIESA, A. 2014. Predicting hypocalcemia after thyroidectomy in children. *Surgery,* 156**,** 130-6.

FRIDMAN, M. V., SAVVA, N. N., KRASKO, O. V., ZBOROVSKAYA, A. A., MANKOVSKAYA, S. V., SCHMID, K. W. & DEMIDCHIK, Y. E. 2012. Clinical and pathologic features of "sporadic" papillary thyroid carcinoma registered in the years 2005 to 2008 in children and adolescents of Belarus. *Thyroid,* 22**,** 1016-24.

GAMBARDELLA, C., OFFI, C., ROMANO, R. M., DE PALMA, M., RUGGIERO, R., CANDELA, G., PUZIELLO, A., DOCIMO, L., GRASSO, M. & DOCIMO, G. 2020. Transcutaneous laryngeal ultrasonography: a reliable, non-invasive and inexpensive preoperative method in the evaluation of vocal cords motility-a prospective multicentric analysis on a large series and a literature review. *Updates Surg,* 72**,** 885-892.

GERTZ, R. J., NIKIFOROV, Y., REHRAUER, W., MCDANIEL, L. & LLOYD, R. V. 2016. Mutation in BRAF and Other Members of the MAPK Pathway in Papillary Thyroid Carcinoma in the Pediatric Population. *Archives of Pathology & Laboratory Medicine,* 140**,** 134-139.

GOLDFARB, M. & FREYER, D. R. 2014. Comparison of secondary and primary thyroid cancer in adolescents and young adults. *Cancer,* 120**,** 1155-61.

GOLDFARB, M., GONDEK, S. S., SANCHEZ, Y. & LEW, J. I. 2012. Clinic-based ultrasound can predict malignancy in pediatric thyroid nodules. *Thyroid,* 22**,** 827-31.

GOLPANIAN, S., PEREZ, E. A., TASHIRO, J., LEW, J. I., SOLA, J. E. & HOGAN, A. R. 2016. Pediatric papillary thyroid carcinoma: outcomes and survival predictors in 2504 surgical patients. *Pediatric Surgery International,* 32**,** 201-208.

GUPTA, A., LY, S., CASTRONEVES, L. A., FRATES, M. C., BENSON, C. B., FELDMAN, H. A., WASSNER, A. J., SMITH, J. R., MARQUSEE, E., ALEXANDER, E. K., BARLETTA, J., DOUBILET, P. M., PETERS, H. E., WEBB, S., MODI, B. P., PALTIEL, H. J., KOZAKEWICH, H., CIBAS, E. S., MOORE, F. D., JR., SHAMBERGER, R. C., LARSEN, P. R. & HUANG, S. A. 2013. A standardized assessment of thyroid nodules in children confirms higher cancer prevalence than in adults. *J Clin Endocrinol Metab,* 98**,** 3238-45.

GUTNICK, J., SOLDES, O., GUPTA, M. & MILAS, M. 2012. Circulating thyrotropin receptor messenger RNA for evaluation of thyroid nodules and surveillance of thyroid cancer in children. *J Pediatr Surg,* 47**,** 171-6.

HANBA, C., SVIDER, P. F., SIEGEL, B., SHEYN, A., SHKOUKANI, M., LIN, H. S. & RAZA, S. N. 2017. Pediatric Thyroidectomy. *Otolaryngol Head Neck Surg,* 156**,** 360-367.

HANDKIEWICZ-JUNAK, D., GAWLIK, T., ROZKOSZ, J., PUCH, Z., MICHALIK, B., GUBALA, E., KRAJEWSKA, J., KLUCZEWSKA, A. & JARZAB, B. 2015. Recombinant human thyrotropin preparation for adjuvant radioiodine treatment in children and adolescents with differentiated thyroid cancer. *Eur J Endocrinol,* 173**,** 873-81.

HANDKIEWICZ-JUNAK, D., WLOCH, J., ROSKOSZ, J., KRAJEWSKA, J., KROPINSKA, A., POMORSKI, L., KUKULSKA, A., PROKURAT, A., WYGODA, Z. & JARZAB, B. 2007. Total thyroidectomy and adjuvant radioiodine treatment independently decrease locoregional recurrence risk in childhood and adolescent differentiated thyroid cancer. *J Nucl Med,* 48**,** 879-88.

HARACH, H. R. & WILLIAMS, E. D. 1995. Childhood thyroid cancer in England and Wales. *Br J Cancer,* 72**,** 777-83.

HAUGEN, B. R., ALEXANDER, E. K., BIBLE, K. C., DOHERTY, G. M., MANDEL, S. J., NIKIFOROV, Y. E., PACINI, F., RANDOLPH, G. W., SAWKA, A. M., SCHLUMBERGER, M., SCHUFF, K. G., SHERMAN, S. I., SOSA, J. A., STEWARD, D. L., TUTTLE, R. M. & WARTOFSKY, L. 2016. 2015 American Thyroid Association Management Guidelines for Adult Patients with Thyroid Nodules and Differentiated Thyroid Cancer: The American Thyroid Association Guidelines Task Force on Thyroid Nodules and Differentiated Thyroid Cancer. *Thyroid,* 26**,** 1-133.

HAVEMAN, J. W., VAN TOL, K. M., ROUWÉ, C. W., PIERS, D. A. & PLUKKER, J. T. M. 2003. Surgical Experience in Children With Differentiated Thyroid Carcinoma. *Annals of Surgical Oncology,* 10**,** 15-20.

HAY, I., GONZALEZ-LOSADA, T., REINALDA, M., HONETSCHLAGER, J., RICHARDS, M. & THOMPSON, G. 2010. Long-Term Outcome in 215 Children and Adolescents with Papillary Thyroid Cancer Treated During 1940 Through 2008. *World J Surg,* 34**,** 1192-1202.

HEIKKINEN, M., HALTTUNEN, S., TERAVA, M., KARKKAINEN, J. M., LOPPONEN, H. & PENTTILA, E. 2019. Vocal foldparesis as a surgical complication: Our 10-year experience with 162 incidents. *Clin Otolaryngol,* 44**,** 179-182.

HENKE, L. E., PERKINS, S. M., PFEIFER, J. D., MA, C., CHEN, Y., DEWEES, T. & GRIGSBY, P. W. 2014. BRAF V600E mutational status in pediatric thyroid cancer. *Pediatr Blood Cancer,* 61**,** 1168-72.

HESS, J., THOMAS, G., BRASELMANN, H., BAUER, V., BOGDANOVA, T., WIENBERG, J., ZITZELSBERGER, H. & UNGER, K. 2011. Gain of chromosome band 7q11 in papillary thyroid carcinomas of young patients is associated with exposure to low-dose irradiation. *Proceedings of the National Academy of Sciences of the United States of America,* 108**,** 9595-600.

HOE, F. M., CHARRON, M. & MOSHANG, T., JR. 2006. Use of the recombinant human TSH stimulated thyroglobulin level and diagnostic whole body scan in children with differentiated thyroid carcinoma. *J Pediatr Endocrinol Metab,* 19**,** 25-30.

HOGAN, A. R., ZHUGE, Y., PEREZ, E. A., KONIARIS, L. G., LEW, J. I. & SOLA, J. E. 2009. Pediatric thyroid carcinoma: incidence and outcomes in 1753 patients. *J Surg Res,* 156**,** 167-72.

HOPERIA, V., LARIN, A., JENSEN, K., BAUER, A. & VASKO, V. 2010. Thyroid fine needle aspiration biopsies in children: study of cytological-histological correlation and immunostaining with thyroid peroxidase monoclonal antibodies. *Int J Pediatr Endocrinol,* 2010**,** 690108.

HOSLER, G. A., CLARK, I., ZAKOWSKI, M. F., WESTRA, W. H. & ALI, S. Z. 2006. Cytopathologic analysis of thyroid lesions in the pediatric population. *Diagn Cytopathol,* 34**,** 101-5.

HUANG, C. H., CHAO, T. C., HSEUH, C., LIN, K. J., HO, T. Y., LIN, S. F. & LIN, J. D. 2012. Therapeutic outcome and prognosis in young patients with papillary and follicular thyroid cancer. *Pediatr Surg Int,* 28**,** 489-94.

IORCANSKY, S., HERZOVICH, V., QUALEY, R. R. & TUTTLE, R. M. 2005. Serum thyrotropin (TSH) levels after recombinant human TSH injections in children and teenagers with papillary thyroid cancer. *J Clin Endocrinol Metab,* 90**,** 6553-5.

ITO, Y., KIHARA, M., TAKAMURA, Y., KOBAYASHI, K., MIYA, A., HIROKAWA, M. & MIYAUCHI, A. 2012. Prognosis and prognostic factors of papillary thyroid carcinoma in patients under 20 years. *Endocrine journal,* 59**,** 539-45.

IZQUIERDO, R., SHANKAR, R., KORT, K. & KHURANA, K. 2009. Ultrasound-guided fine-needle aspiration in the management of thyroid nodules in children and adolescents. *Thyroid : official journal of the American Thyroid Association,* 19**,** 703-5.

JANG, H. W., LEE, J. I., KIM, H. K., OH, Y. L., CHOI, Y. L., JIN, D. K., KIM, J. H., CHUNG, J. H. & KIM, S. W. 2012. Identification of a cut-off for the MACIS score to predict the prognosis of differentiated thyroid carcinoma in children and young adults. *Head Neck,* 34**,** 696-701.

JARZAB, B., HANDKIEWICZ-JUNAK, D. & WLOCH, J. 2005. Juvenile differentiated thyroid carcinoma and the role of radioiodine in its treatment: a qualitative review. *Endocr Relat Cancer,* 12**,** 773-803.

JARZAB, B., JUNAK, D. H., JAN, W., KALEMBA, B., ROSKOSZ, J. & KUKULSKA, A. 2000. Multivariate analysis of prognostic factors for differentiated thyroid carcinoma in children. *European journal of nuclear medicine,* 27.

JIANG, W., NEWBURY, R. O. & NEWFIELD, R. S. 2016. Pediatric thyroid surgery and management of thyroid nodules – an institutional experience over a 10-year period. *International Journal of Pediatric Endocrinology,* 2016**,** 1.

JIN, X., MASTERSON, L., PATEL, A., HOOK, L., NICHOLSON, J., JEFFERIES, S., GAZE, M., NASSIF, R., ELLER, R., HULSE, T. & JANI, P. 2015. Conservative or radical surgery for pediatric papillary thyroid carcinoma: A systematic review of the literature. *Int J Pediatr Otorhinolaryngol,* 79**,** 1620-4.

JOLIAT, G. R., GUARNERO, V., DEMARTINES, N., SCHWEIZER, V. & MATTER, M. 2017. Recurrent laryngeal nerve injury after thyroid and parathyroid surgery: Incidence and postoperative evolution assessment. *Medicine (Baltimore),* 96**,** e6674.

KAPILA, K., PATHAN, S. K., GEORGE, S. S., HAJI, B. E., DAS, D. K. & QADAN, L. R. 2010. Fine Needle Aspiration Cytology of the Thyroid in Children and Adolescents Experience with 792 Aspirates. *Acta cytologica,* 54**,** 569.

KENNEDY, R. D., POTTER, D. D., MOIR, C. R. & EL-YOUSSEF, M. 2014. The natural history of familial adenomatous polyposis syndrome: a 24 year review of a single center experience in screening, diagnosis, and outcomes. *J Pediatr Surg,* 49**,** 82-6.

KHURANA, K. K., LABRADOR, E., IZQUIERDO, R., MESONERO, C. E. & PISHARODI, L. R. 1999. The Role of Fine-Needle Aspiration Biopsy in the Management of Thyroid Nodules in Children, Adolescents, and Young Adults: A Multi-Institutional Study. *Thyroid,* 9**,** 383-386.

KIM, H. Y., GELFAND, M. J. & SHARP, S. E. 2011. SPECT/CT imaging in children with papillary thyroid carcinoma. *Pediatr Radiol,* 41**,** 1008-12.

KIM, J., SUN, Z., ADAM, M. A., ADIBE, O. O., RICE, H. E., ROMAN, S. A. & TRACY, E. T. 2017. Predictors of nodal metastasis in pediatric differentiated thyroid cancer. *J Pediatr Surg,* 52**,** 120-123.

KIRATLI, P. O., VOLKAN-SALANCI, B., GUNAY, E. C., VARAN, A., AKYUZ, C. & BUYUKPAMUKCU, M. 2013. Thyroid Cancer in Pediatric Age Group An Institutional Experience and Review of the Literature. *Journal of pediatric hematology/oncology,* 35**,** 93-97.

KIRK, J. M., MORT, C., GRANT, D. B., TOUZEL, R. J. & PLOWMAN, N. 1992. The usefulness of serum thyroglobulin in the follow-up of differentiated thyroid carcinoma in children. *Medical and pediatric oncology,* 20**,** 201-208.

KLEIN HESSELINK, M. S., NIES, M., BOCCA, G., BROUWERS, A. H., BURGERHOF, J. G. M., VAN DAM, E. W. C. M., HAVEKES, B., VAN DEN HEUVEL-EIBRINK, M. M., CORSSMIT, E. P. M., KREMER, L. C. M., NETEA-MAIER, R. T., VAN DER PAL, H. J. H., PEETERS, R. P., SCHMID, K. W., SMIT, J. W. A., WILLIAMS, G. R., PLUKKER, J. T. M., RONCKERS, C. M.,

VAN SANTEN, H. M., TISSING, W. J. E. & LINKS, T. P. 2016. Pediatric Differentiated Thyroid Carcinoma in The Netherlands: A Nationwide Follow-Up Study. *The Journal of Clinical Endocrinology & Metabolism,* 101**,** 2031-2039.
KOLTIN, D., O'GORMAN, C. S., MURPHY, A., NGAN, B., DANEMAN, A., NAVARRO, O. M., GARCIA, C., ATENAFU, E. G., WASSERMAN, J. D., HAMILTON, J. & RACHMIEL, M. 2016. Pediatric thyroid nodules: ultrasonographic characteristics and inter-observer variability in prediction of malignancy. *J Pediatr Endocrinol Metab,* 29**,** 789-94.
KOO, J. S., HONG, S. & PARK, C. S. 2009. Diffuse sclerosing variant is a major subtype of papillary thyroid carcinoma in the young. *Thyroid,* 19**,** 1225-31.
KOWALSKI, L. P., GONCALVES FILHO, J., PINTO, C. A., CARVALHO, A. L. & DE CAMARGO, B. 2003. Long-term survival rates in young patients with thyroid carcinoma. *Arch Otolaryngol Head Neck Surg,* 129**,** 746-9.
KUIJT, W. J. & HUANG, S. A. 2005. Children with differentiated thyroid cancer achieve adequate hyperthyrotropinemia within 14 days of levothyroxine withdrawal. *J Clin Endocrinol Metab,* 90**,** 6123-5.
LA QUAGLIA, M. P., BLACK, T., HOLCOMB, G. W. R., SKLAR, C., AZIZKHAN, R. G., HAASE, G. M. & NEWMAN, K. D. 2000. Differentiated thyroid cancer clinical characteristics, treatment, and outcome in patients under 21 years of age Who Present With Distant Metastases.

A Report From the Surgical Discipline Committee of the Children's Cancer Group. *Journal of Pediatric Surgery,* 35**,** 955-960.
LALE, S. A., MORGENSTERN, N. N., CHIARA, S. & WASSERMAN, P. 2015. Fine needle aspiration of thyroid nodules in the pediatric population: a 12-year cyto-histological correlation experience at North Shore-Long Island Jewish Health System. *Diagn Cytopathol,* 43**,** 598-604.
LANDAU, D., VINI, L., A'HERN, R. & HARMER, C. 2000. Thyroid cancer in children the Royal Marsden Hospital experience. *european journal of cancer,* 36**,** 214-220.
LANG, B. H., CHU, K. K., TSANG, R. K., WONG, K. P. & WONG, B. Y. 2014. Evaluating the incidence, clinical significance and predictors for vocal cord palsy and incidental laryngopharyngeal conditions before elective thyroidectomy: is there a case for routine laryngoscopic examination? *World J Surg,* 38**,** 385-91.
LAUPER, J. M., KRAUSE, A., VAUGHAN, T. L. & MONNAT, R. J., JR. 2013. Spectrum and risk of neoplasia in Werner syndrome: a systematic review. *PLoS One,* 8**,** e59709.
LAZAR, L., LEBENTHAL, Y., STEINMETZ, A., YACKOBOVITCH-GAVAN, M. & PHILLIP, M. 2009. Differentiated Thyroid Carcinoma in Pediatric Patients: Comparison of Presentation and Course between Pre-pubertal Children and Adolescents. *The Journal of Pediatrics,* 154**,** 708.
LEE, K. A., SHARABIANI, M. T. A., TUMINO, D., WADSLEY, J., GILL, V., GERRARD, G., SINDHU, R., GAZE, M. N., MOSS, L. & NEWBOLD, K. 2019. Differentiated Thyroid Cancer in Children: A UK Multicentre Review and Review of the Literature. *Clin Oncol (R Coll Radiol),* 31**,** 385-390.
LEE, Y. A., JUNG, H. W., KIM, H. Y., CHOI, H., KIM, H.-Y., HAH, J. H., PARK, D. J., CHUNG, J.-K., YANG, S. W., SHIN, C. H. & PARK, Y. J. 2015. Pediatric patients with multifocal papillary thyroid cancer have higher recurrence rates than adult patients: a retrospective analysis of a large pediatric thyroid cancer cohort over 33 years. *The Journal of clinical endocrinology and metabolism,* 100**,** 1619-29.
LEEMAN-NEILL, R. J., BRENNER, A. V., LITTLE, M. P., BOGDANOVA, T. I., HATCH, M., ZURNADZY, L. Y., MABUCHI, K., TRONKO, M. D. & NIKIFOROV, Y. E. 2013. RET/PTC and PAX8/PPARgamma chromosomal rearrangements in post-Chernobyl thyroid cancer and their association with iodine-131 radiation dose and other characteristics. *Cancer,* 119**,** 1792-9.

LERNER, J. & GOLDFARB, M. 2015a. Follicular variant papillary thyroid carcinoma in a pediatric population. *Pediatric Blood & Cancer,* 62**,** 1942-1946.
LERNER, J. & GOLDFARB, M. 2015b. Pediatric Thyroid Microcarcinoma. *Annals of Surgical Oncology,* 22**,** 4187-4192.
LO, C. Y., KWOK, K. F. & YUEN, P. W. 2000. A prospective evaluation of recurrent laryngeal nerve paralysis during thyroidectomy. *Arch Surg,* 135**,** 204-7.
LUSTER, M., HANDKIEWICZ-JUNAK, D., GROSSI, A., ZACHARIN, M., TAIEB, D., CRUZ, O., HITZEL, A., CASAS, J. A., MADER, U., DOTTORINI, M. E. & PEDIATRIC RH, T. S. H. I. G. 2009. Recombinant thyrotropin use in children and adolescents with differentiated thyroid cancer: a multicenter retrospective study. *J Clin Endocrinol Metab,* 94**,** 3948-53.
LY, S., FRATES, M. C., BENSON, C. B., PETERS, H. E., GRANT, F. D., DRUBACH, L. A., VOSS, S. D., FELDMAN, H. A., SMITH, J. R., BARLETTA, J., HOLLOWELL, M., CIBAS, E. S., MOORE, F. D., MODI, B., SHAMBERGER, R. C. & HUANG, S. A. 2016. Features and Outcome of Autonomous Thyroid Nodules in Children: 31 Consecutive Patients Seen at a Single Center. *The Journal of Clinical Endocrinology & Metabolism,* 101**,** 3856-3862.
LYSHCHIK, A., DROZD, V., DEMIDCHIK, Y. & REINERS, C. 2005. Diagnosis of thyroid cancer in children: value of gray-scale and power doppler US. *Radiology,* 235**,** 604-13.
MACHENS, A., ELWERR, M., THANH, P. N., LORENZ, K., SCHNEIDER, R. & DRALLE, H. 2016. Impact of central node dissection on postoperative morbidity in pediatric patients with suspected or proven thyroid cancer. *Surgery,* 160**,** 484-92.
MACHENS, A., LORENZ, K., NGUYEN THANH, P., BRAUCKHOFF, M. & DRALLE, H. 2010. Papillary thyroid cancer in children and adolescents does not differ in growth pattern and metastatic behavior. *J Pediatr,* 157**,** 648-52.
MALLICK, U., HARMER, C., HACKSHAW, A., MOSS, L. & IO, N. T. M. G. 2012a. Iodine or Not (IoN) for low-risk differentiated thyroid cancer: the next UK National Cancer Research Network randomised trial following HiLo. *Clin Oncol (R Coll Radiol),* 24**,** 159-61.
MALLICK, U., HARMER, C., YAP, B., WADSLEY, J., CLARKE, S., MOSS, L., NICOL, A., CLARK, P. M., FARNELL, K., MCCREADY, R., SMELLIE, J., FRANKLYN, J. A., JOHN, R., NUTTING, C. M., NEWBOLD, K., LEMON, C., GERRARD, G., ABDEL-HAMID, A., HARDMAN, J., MACIAS, E., ROQUES, T., WHITAKER, S., VIJAYAN, R., ALVAREZ, P., BEARE, S., FORSYTH, S., KADALAYIL, L. & HACKSHAW, A. 2012b. Ablation with low-dose radioiodine and thyrotropin alfa in thyroid cancer. *N Engl J Med,* 366**,** 1674-85.
MARKOVINA, S., GRIGSBY, P. W., SCHWARZ, J. K., DEWEES, T., MOLEY, J. F., SIEGEL, B. A. & PERKINS, S. M. 2014. Treatment approach, surveillance, and outcome of well-differentiated thyroid cancer in childhood and adolescence. *Thyroid,* 24**,** 1121-6.
MARSH, D. J., COULON, V., LUNETTA, K. L., ROCCA-SERRA, P., DAHIA, P. L., ZHENG, Z., LIAW, D., CARON, S., DUBOUE, B., LIN, A. Y., RICHARDSON, A. L., BONNETBLANC, J. M., BRESSIEUX, J. M., CABARROT-MOREAU, A., CHOMPRET, A., DEMANGE, L., EELES, R. A., YAHANDA, A. M., FEARON, E. R., FRICKER, J. P., GORLIN, R. J., HODGSON, S. V., HUSON, S., LACOMBE, D., ENG, C. & ET AL. 1998. Mutation spectrum and genotype-phenotype analyses in Cowden disease and Bannayan-Zonana syndrome, two hamartoma syndromes with germline PTEN mutation. *Hum Mol Genet,* 7**,** 507-15.
MASSIMINO, M., COLLINI, P., LEITE, S. F., SPREAFICO, F., ZUCCHINI, N., FERRARI, A., MATTAVELLI, F., SEREGNI, E., CASTELLANI, M. R., CANTU, G., FOSSATI-BELLANI, F., ROSAI, J. & THYROID CANCER STUDY GROUP OF THE ISTITUTO NAZIONALE TUMORI OF MILAN, I. 2006. Conservative surgical approach for thyroid and lymph-node involvement in papillary thyroid carcinoma of childhood and adolescence. *Pediatr Blood Cancer,* 46**,** 307-13.

METZGER, M. L., HOWARD, S. C., HUDSON, M. M., GOW, K. W., LI, C. S., KRASIN, M. J., MERCHANT, T., KUN, L., SHELSO, J., PUI, C. H., SHOCHAT, S. J. & MCCARVILLE, M. B. 2006. Natural history of thyroid nodules in survivors of pediatric Hodgkin lymphoma. *Pediatr Blood Cancer,* 46**,** 314-9.

MIHAILOVIC, J., NIKOLETIC, K. & SRBOVAN, D. 2014. Recurrent disease in juvenile differentiated thyroid carcinoma: prognostic factors, treatments, and outcomes. *J Nucl Med,* 55**,** 710-7.

MONACO, S. E., PANTANOWITZ, L., KHALBUSS, W. E., BENKOVICH, V. A., OZOLEK, J., NIKIFOROVA, M. N., SIMONS, J. P. & NIKIFOROV, Y. E. 2012. Cytomorphological and molecular genetic findings in pediatric thyroid fine-needle aspiration. *Cancer Cytopathol,* 120**,** 342-50.

MORRIS, L. F., WAGUESPACK, S. G., WARNEKE, C. L., RYU, H., YING, A. K., ANDERSON, B. J., STURGIS, E. M., CLAYMAN, G. L., LEE, J. E., EVANS, D. B., GRUBBS, E. G. & PERRIER, N. D. 2012. Long-term follow-up data may help manage patient and parent expectations for pediatric patients undergoing thyroidectomy. *Surgery,* 152**,** 1165-71.

MORRISON, P. J. & ATKINSON, A. B. 2009. Genetic aspects of familial thyroid cancer. *Oncologist,* 14**,** 571-7.

MOSTAFA, M., VALI, R., CHAN, J., OMARKHAIL, Y. & SHAMMAS, A. 2016. Variants and pitfalls on radioiodine scans in pediatric patients with differentiated thyroid carcinoma. *Pediatric Radiology,* 46**,** 1579-1589.

MOUDGIL, P., VELLODY, R., HEIDER, A., SMITH, E. A., GROVE, J. J., JARBOE, M. D., BRUCH, S. W. & DILLMAN, J. R. 2016. Ultrasound-guided fine-needle aspiration biopsy of pediatric thyroid nodules. *Pediatric Radiology,* 46**,** 365-371.

MUSSA, A., DE ANDREA, M., MOTTA, M., MORMILE, A., PALESTINI, N. & CORRIAS, A. 2015. Predictors of Malignancy in Children with Thyroid Nodules. *J Pediatr,* 167**,** 886-892 e1.

MUSSA, A., SALERNO, M. C., BONA, G., WASNIEWSKA, M., SEGNI, M., CASSIO, A., VIGONE, M. C., GASTALDI, R., IUGHETTI, L., SANTANERA, A., CAPALBO, D., MATARAZZO, P., DE LUCA, F., WEBER, G. & CORRIAS, A. 2013. Serum thyrotropin concentration in children with isolated thyroid nodules. *J Pediatr,* 163**,** 1465-70.

NEWMAN, K. D., BLACK, T., HELLER, G., AZIZKHAN, R. G., HOLCOMB, G. W., SKLAR, C., VMAMIS, V., HAASE, G. M. & QUAGLIA, M. P. L. 1998. Differentiated Thyroid Cancer Determinants of Disease Progression in Patients 21 Years of Age at Diagnosis A Report from the Surgical Discipline Committee of the Children ' s Cancer Group. *Ann Surg,* 227**,** 533-541.

NGEOW, J., MESTER, J., RYBICKI, L. A., NI, Y., MILAS, M. & ENG, C. 2011. Incidence and clinical characteristics of thyroid cancer in prospective series of individuals with Cowden and Cowden-like syndrome characterized by germline PTEN, SDH, or KLLN alterations. *J Clin Endocrinol Metab,* 96**,** E2063-71.

NICE, T., PASARA, S., GOLDFARB, M., DOSKI, J., GOLDIN, A., GOW, K. W., NUCHTERN, J. G., VASUDEVAN, S. A., LANGER, M. & BEIERLE, E. A. 2015. Pediatric papillary thyroid cancer >1 cm: is total thyroidectomy necessary? *J Pediatr Surg,* 50**,** 1009-13.

NIKIFOROV, Y. E. & GNEPP, M. D. 1994. Pediatric Thyroid Cancer after the

Chernobyl Disaster. *Cancer,* 74**,** 748-766.

NIKIFOROV, Y. E., SEETHALA, R. R., TALLINI, G., BALOCH, Z. W., BASOLO, F., THOMPSON, L. D., BARLETTA, J. A., WENIG, B. M., AL GHUZLAN, A., KAKUDO, K., GIORDANO, T. J., ALVES, V. A., KHANAFSHAR, E., ASA, S. L., EL-NAGGAR, A. K., GOODING, W. E., HODAK, S. P., LLOYD, R. V., MAYTAL, G., METE, O., NIKIFOROVA, M. N., NOSE, V., PAPOTTI, M., POLLER, D. N., SADOW, P. M., TISCHLER, A. S., TUTTLE, R. M., WALL, K. B., LIVOLSI, V. A., RANDOLPH, G. W. & GHOSSEIN, R. A. 2016. Nomenclature Revision

for Encapsulated Follicular Variant of Papillary Thyroid Carcinoma: A Paradigm Shift to Reduce Overtreatment of Indolent Tumors. *JAMA Oncol,* 2**,** 1023-9.
NORLEN, O., CHARLTON, A., SARKIS, L. M., HENWOOD, T., SHUN, A., GILL, A. J. & DELBRIDGE, L. 2015. Risk of malignancy for each Bethesda class in pediatric thyroid nodules. *J Pediatr Surg,* 50**,** 1147-9.
O'GORMAN, C. S., HAMILTON, J., RACHMIEL, M., GUPTA, A., NGAN, B. Y. & DANEMAN, D. 2010. Thyroid cancer in childhood: a retrospective review of childhood course. *Thyroid,* 20**,** 375-80.
OOMMEN, P. T., ROMAHN, A., LINDEN, T., FRUHWALD, M. C. & BUCSKY, P. 2008. UICC-2002 TNM classification is not suitable for differentiated thyroid cancer in children and adolescents. *Pediatr Blood Cancer,* 50**,** 1159-62.
OREN, A., BENOIT, M. A., MURPHY, A., SCHULTE, F. & HAMILTON, J. 2012. Quality of life and anxiety in adolescents with differentiated thyroid cancer. *J Clin Endocrinol Metab,* 97**,** E1933-7.
PACINI, F., MOLINARO, E., CASTAGNA, M. G., AGATE, L., ELISEI, R., CECCARELLI, C., LIPPI, F., TADDEI, D., GRASSO, L. & PINCHERA, A. 2003. Recombinant human thyrotropin-stimulated serum thyroglobulin combined with neck ultrasonography has the highest sensitivity in monitoring differentiated thyroid carcinoma. *J Clin Endocrinol Metab,* 88**,** 3668-73.
PALMER, B. A., ZARROUG, A. E., POLEY, R. N., KOLLARS, J. P. & MOIR, C. R. 2005. Papillary thyroid carcinoma in children: risk factors and complications of disease recurrence. *J Pediatr Surg,* 40**,** 1284-8.
PAPENDIECK, P., GRUÑEIRO-PAPENDIECK, L., VENARA, M., ACHA, O., COZZANI, H., MATEOS, F., MAGLIO, S., CALCAGNO, M. L., BERGADA, I. & CHIESA, A. 2015. Differentiated Thyroid Cancer in Children: Prevalence and Predictors in a Large Cohort with Thyroid Nodules Followed Prospectively. *The Journal of Pediatrics,* 167**,** 199-201.
PAPENDIECK, P., GRUÑEIRO-PAPENDIECK, L., VENARA, M., ACHA, O., MAGLIO, S., BERGADÁ, I. & CHIESA, A. 2011. Differentiated thyroid carcinoma: presentation and follow-up in children and adolescents. *Journal of Pediatric Endocrinology and Metabolism,* 24.
PARTYKA, K. L., HUANG, E. C., CRAMER, H. M., CHEN, S. & WU, H. H. 2016. Histologic and clinical follow-up of thyroid fine-needle aspirates in pediatric patients. *Cancer Cytopathology,* 124**,** 467-471.
PATEL, A. 2002. Differentiated Thyroid Carcinoma That Express Sodium-Iodide Symporter Have a Lower Risk of Recurrence for Children and Adolescents. *Pediatric Research,* 52**,** 737-744.
PATEL, N. A., BLY, R. A., ADAMS, S., CARLIN, K., PARIKH, S. R., DAHL, J. P. & MANNING, S. 2018. A clinical pathway for the postoperative management of hypocalcemia after pediatric thyroidectomy reduces blood draws. *Int J Pediatr Otorhinolaryngol,* 105**,** 132-137.
PAWELCZAK, M., DAVID, R., FRANKLIN, B., KESSLER, M., LAM, L. & SHAH, B. 2010. Outcomes of children and adolescents with well-differentiated thyroid carcinoma and pulmonary metastases following (1)(3)(1)I treatment: a systematic review. *Thyroid,* 20**,** 1095-101.
PEILING YANG, S. & NGEOW, J. 2016. Familial non-medullary thyroid cancer: unraveling the genetic maze. *Endocr Relat Cancer,* 23**,** R577-R595.
PENKO, K., LIVEZEY, J., FENTON, C., PATEL, A., NICHOLSON, D., FLORA, M., OAKLEY, K., TUTTLE, R. M. & FRANCIS, G. 2005. BRAF mutations are uncommon in papillary thyroid cancer of young patients. *Thyroid,* 15**,** 320-325.
PERROS, P., BOELAERT, K., COLLEY, S., EVANS, C., EVANS, R. M., GERRARD BA, G., GILBERT, J., HARRISON, B., JOHNSON, S. J., GILES, T. E., MOSS, L., LEWINGTON, V., NEWBOLD, K., TAYLOR, J., THAKKER, R. V., WATKINSON, J., WILLIAMS, G. R. & BRITISH THYROID, A.

2014. Guidelines for the management of thyroid cancer. *Clin Endocrinol (Oxf),* 81 Suppl 1**,** 1-122.
PLUIJMEN, M. J., EUSTATIA-RUTTEN, C., GOSLINGS, B. M., STOKKEL, M. P., ARIAS, A. M., DIAMANT, M., ROMIJN, J. A. & SMIT, J. W. 2003. Effects of low-iodide diet on postsurgical radioiodide ablation therapy in patients with differentiated thyroid carcinoma. *Clin Endocrinol (Oxf),* 58**,** 428-35.
POPOVTZER, A., SHPITZER, T., BAHAR, G., FEINMESSER, R. & SEGAL, K. 2006. Thyroid cancer in children: management and outcome experience of a referral center. *Otolaryngol Head Neck Surg,* 135**,** 581-4.
POWERS, P. A., DINAUER, C. A., TUTTLE, R. M. & FRANCIS, G. L. 2004. The MACIS score predicts the clinical course of papillary thyroid carcinoma in children and adolescents. *Journal of pediatric endocrinology & metabolism,* 17**,** 339-343.
POWERS, P. A., DINAUER, C. A., TUTTLE, R. M., ROBIE, D. K., MCCLELLAN, D. R. & FRANCIS, G. L. 2003. Tumor Size and Extent of Disease at Diagnosis Predict the Response to Initial Therapy for Papillary Thyroid Carcinoma in Children and Adolescents. *J Pediatr Endocrinol Metab,* 16**,** 693-702.
PRASAD, M. L., VYAS, M., HORNE, M. J., VIRK, R. K., MOROTTI, R., LIU, Z., TALLINI, G., NIKIFOROVA, M. N., CHRISTISON-LAGAY, E. R., UDELSMAN, R., DINAUER, C. A. & NIKIFOROV, Y. E. 2016. NTRK fusion oncogenes in pediatric papillary thyroid carcinoma in northeast United States. *Cancer,* 122**,** 1097-107.
QICHANG, W., LIN, B., GEGE, Z., YOUJIA, Z., QINGJIE, M., RENJIE, W. & BIN, J. 2019. Diagnostic performance of 18F-FDG-PET/CT in DTC patients with thyroglobulin elevation and negative iodine scintigraphy: a meta-analysis. *Eur J Endocrinol,* 181**,** 93-102.
QU, N., ZHANG, L., LU, Z.-W., JI, Q.-H., YANG, S.-W., WEI, W.-J. & ZHANG, Y. 2016. Predictive factors for recurrence of differentiated thyroid cancer in patients under 21 years of age and a meta-analysis of the current literature. *Tumor Biology,* 37**,** 7797-7808.
RAAB, S. S., SILVERMAN, J. F., ELSHEIKH, T. M., THOMAS, P. A., PAUL, E., RAAB, S. & THOMAS, A. 1995. Pediatric Thyroid Nodules Disease Demographics and Clinical Management as Determined by Fine Needle Aspiration Bio. *Pediatrics,* 95**,** 46.
RAVAL, M. V., BENTREM, D. J., STEWART, A. K., KO, C. Y. & REYNOLDS, M. 2010. Utilization of total thyroidectomy for differentiated thyroid cancer in children. *Ann Surg Oncol,* 17**,** 2545-53.
REDLICH, A., BOXBERGER, N., SCHMID, K. W., FRUHWALD, M., ROHRER, T. & VORWERK, P. 2012. Sensitivity of fine-needle biopsy in detecting pediatric differentiated thyroid carcinoma. *Pediatr Blood Cancer,* 59**,** 233-7.
RICARTE-FILHO, J. C., LI, S., GARCIA-RENDUELES, M. E., MONTERO-CONDE, C., VOZA, F., KNAUF, J. A., HEGUY, A., VIALE, A., BOGDANOVA, T., THOMAS, G. A., MASON, C. E. & FAGIN, J. A. 2013. Identification of kinase fusion oncogenes in post-Chernobyl radiation-induced thyroid cancers. *J Clin Invest,* 123**,** 4935-44.
RICHARDS, M. L. 2010. Familial syndromes associated with thyroid cancer in the era of personalized medicine. *Thyroid,* 20**,** 707-13.
RIVKEES, S. A., MAZZAFERRI, E. L., VERBURG, F. A., REINERS, C., LUSTER, M., BREUER, C. K., DINAUER, C. A. & UDELSMAN, R. 2011. The treatment of differentiated thyroid cancer in children: emphasis on surgical approach and radioactive iodine therapy. *Endocr Rev,* 32**,** 798-826.
ROSARIO, P. W., MINEIRO FILHO, A. F., LACERDA, R. X. & CALSOLARI, M. R. 2012. Recombinant human TSH for thyroid remnant ablation with (131)I in children and adolescents with papillary carcinoma. *Horm Res Paediatr,* 77**,** 59-62.

ROSE, J., WERTHEIM, B. C. & GUERRERO, M. A. 2012. Radiation treatment of patients with primary pediatric malignancies: risk of developing thyroid cancer as a secondary malignancy. *Am J Surg,* 204**,** 881-6; discussion 886-7.

ROSENBAUM, E., HOSLER, G., ZAHURAK, M., COHEN, Y., SIDRANSKY, D. & WESTRA, W. H. 2005. Mutational activation of BRAF is not a major event in sporadic childhood papillary thyroid carcinoma. *Mod Pathol,* 18**,** 898-902.

ROSSI, E. D., STRACCIA, P., MARTINI, M., REVELLI, L., LOMBARDI, C. P., PONTECORVI, A. & FADDA, G. 2014. The role of thyroid fine-needle aspiration cytology in the pediatric population: an institutional experience. *Cancer Cytopathol,* 122**,** 359-67.

RUTTER, M. M., JHA, P., SCHULTZ, K. A., SHEIL, A., HARRIS, A. K., BAUER, A. J., FIELD, A. L., GELLER, J. & HILL, D. A. 2016. DICER1 Mutations and Differentiated Thyroid Carcinoma: Evidence of a Direct Association. *J Clin Endocrinol Metab,* 101**,** 1-5.

SAAVEDRA, J., DELADOEY, J., SAINT-VIL, D., BOIVIN, Y., ALOS, N., DEAL, C., VAN VLIET, G. & HUOT, C. 2011. Is ultrasonography useful in predicting thyroid cancer in children with thyroid nodules and apparently benign cytopathologic features? *Horm Res Paediatr,* 75**,** 269-75.

SAMUELS, S. L., SURREY, L. F., HAWKES, C. P., AMBERGE, M., MOSTOUFI-MOAB, S., LANGER, J. E., ADZICK, N. S., KAZAHAYA, K., BHATTI, T., BALOCH, Z., LIVOLSI, V. A. & BAUER, A. J. 2018. Characteristics of Follicular Variant Papillary Thyroid Carcinoma in a Pediatric Cohort. *J Clin Endocrinol Metab,* 103**,** 1639-1648.

SANTOS, J. E., FREITAS, M., FONSECA, C. P., CASTILHO, P., CARREIRA, I. M., ROMBEAU, J. L. & BRANCO, M. C. 2017. Iodine deficiency a persisting problem: assessment of iodine nutrition and evaluation of thyroid nodular pathology in Portugal. *J Endocrinol Invest,* 40**,** 185-191.

SASSOLAS, G., HAFDI-NEJJARI, Z., FERRARO, A., DECAUSSIN-PETRUCCI, M., ROUSSET, B., BORSON-CHAZOT, F., BORBONE, E., BERGER, N. & FUSCO, A. 2012. Oncogenic alterations in papillary thyroid cancers of young patients. *Thyroid,* 22**,** 17-26.

SAVIO, R., GOSNELL, J., PALAZZO, F. F., SYWAK, M., AGARWAL, G., COWELL, C., SHUN, A., ROBINSON, B. & DELBRIDGE, L. W. 2005. The role of a more extensive surgical approach in the initial multimodality management of papillary thyroid cancer in children. *J Pediatr Surg,* 40**,** 1696-700.

SCHLUMBERGER, M., CATARGI, B., BORGET, I., DEANDREIS, D., ZERDOUD, S., BRIDJI, B., BARDET, S., LEENHARDT, L., BASTIE, D., SCHVARTZ, C., VERA, P., MOREL, O., BENISVY, D., BOURNAUD, C., BONICHON, F., DEJAX, C., TOUBERT, M. E., LEBOULLEUX, S., RICARD, M., BENHAMOU, E. & TUMEURS DE LA THYROIDE REFRACTAIRES NETWORK FOR THE ESSAI STIMULATION ABLATION EQUIVALENCE, T. 2012. Strategies of radioiodine ablation in patients with low-risk thyroid cancer. *N Engl J Med,* 366**,** 1663-73.

SCHLUMBERGER, M., TAHARA, M., WIRTH, L. J., ROBINSON, B., BROSE, M. S., ELISEI, R., HABRA, M. A., NEWBOLD, K., SHAH, M. H., HOFF, A. O., GIANOUKAKIS, A. G., KIYOTA, N., TAYLOR, M. H., KIM, S. B., KRZYZANOWSKA, M. K., DUTCUS, C. E., DE LAS HERAS, B., ZHU, J. & SHERMAN, S. I. 2015. Lenvatinib versus placebo in radioiodine-refractory thyroid cancer. *N Engl J Med,* 372**,** 621-30.

SCHNEIDER, P., BIKO, J., REINERS, C., DEMIDCHIK, Y. E., DROZD, V. M., CAPOZZA, R. F., COINTRY, G. R. & FERRETTI, J. L. 2004. Impact of parathyroid status and Ca and vitamin-D supplementation on bone mass and muscle-bone relationships in 208 Belarussian children after thyroidectomy because of thyroid carcinoma. *Experimental and clinical endocrinology & diabetes : official journal, German Society of Endocrinology [and] German Diabetes Association,* 112**,** 444.

SCHNEIDER, R., MACHENS, A., SEKULLA, C., LORENZ, K., WEBER, F. & DRALLE, H. 2018. Twenty-year experience of paediatric thyroid surgery using intraoperative nerve monitoring. *Br J Surg,* 105**,** 996-1005.

SCHOLZ, S., SMITH, J. R., CHAIGNAUD, B., SHAMBERGER, R. C. & HUANG, S. A. 2011. Thyroid surgery at Children's Hospital Boston: a 35-year single-institution experience. *J Pediatr Surg,* 46**,** 437-42.

SHAYOTA, B. J., PAWAR, S. C. & CHAMBERLAIN, R. S. 2013. MeSS: A novel prognostic scale specific for pediatric well-differentiated thyroid cancer: a population-based, SEER outcomes study. *Surgery,* 154**,** 429-35.

SIGURDSON, A. J., RONCKERS, C. M., MERTENS, A. C., STOVALL, M., SMITH, S. A., LIU, Y., BERKOW, R. L., HAMMOND, S., NEGLIA, J. P., MEADOWS, A. T., SKLAR, C. A., ROBISON, L. L. & INSKIP, P. D. 2005. Primary thyroid cancer after a first tumour in childhood (the Childhood Cancer Survivor Study): a nested case-control study. *The Lancet,* 365**,** 2014-2023.

SILVA-VIEIRA, M., SANTOS, R., LEITE, V. & LIMBERT, E. 2015. Review of clinical and pathological features of 93 cases of well-differentiated thyroid carcinoma in pediatric age at the Lisbon Centre of the Portuguese Institute of Oncology between 1964 and 2006. *International Journal of Pediatric Otorhinolaryngology,* 79**,** 1324-1329.

SINCLAIR, C. F., BUMPOUS, J. M., HAUGEN, B. R., CHALA, A., MELTZER, D., MILLER, B. S., TOLLEY, N. S., SHIN, J. J., WOODSON, G. & RANDOLPH, G. W. 2016. Laryngeal examination in thyroid and parathyroid surgery: An American Head and Neck Society consensus statement: AHNS Consensus Statement. *Head Neck,* 38**,** 811-9.

SMALLRIDGE, R. C., MEEK, S. E., MORGAN, M. A., GATES, G. S., FOX, T. P., GREBE, S. & FATOURECHI, V. 2007. Monitoring thyroglobulin in a sensitive immunoassay has comparable sensitivity to recombinant human tsh-stimulated thyroglobulin in follow-up of thyroid cancer patients. *J Clin Endocrinol Metab,* 92**,** 82-7.

SMITH, J. R., MARQUSEE, E., WEBB, S., NOSE, V., FISHMAN, S. J., SHAMBERGER, R. C., FRATES, M. C. & HUANG, S. A. 2011. Thyroid nodules and cancer in children with PTEN hamartoma tumor syndrome. *J Clin Endocrinol Metab,* 96**,** 34-7.

SMITH, M., PANTANOWITZ, L., KHALBUSS, W. E., BENKOVICH, V. A. & MONACO, S. E. 2013. Indeterminate pediatric thyroid fine needle aspirations: a study of 68 cases. *Acta Cytol,* 57**,** 341-8.

SOBERMAN, N., LEONIDAS, J. C., CHERRICK, I., SCHIFF, R. & KARAYALCIN, G. 1991. Sonographic abnormalities of the thyroid gland in longterm survivors of Hodgkin disease. *Pediatr Radiol,* 21**,** 250-3.

SOLT, I., GAITINI, D., PERY, M., HOCHBERG, Z., STEIN, M. & ARUSH, M. W. 2000. Comparing thyroid ultrasonography to thyroid function in long-term survivors of childhood lymphoma. *Med Pediatr Oncol,* 35**,** 35-40.

SOSA, J. A., TUGGLE, C. T., WANG, T. S., THOMAS, D. C., BOUDOURAKIS, L., RIVKEES, S. & ROMAN, S. A. 2008. Clinical and economic outcomes of thyroid and parathyroid surgery in children. *J Clin Endocrinol Metab,* 93**,** 3058-65.

SPENCER, C., FATEMI, S., SINGER, P., NICOLOFF, J. & LOPRESTI, J. 2010. Serum Basal thyroglobulin measured by a second-generation assay correlates with the recombinant human thyrotropin-stimulated thyroglobulin response in patients treated for differentiated thyroid cancer. *Thyroid,* 20**,** 587-95.

SPENCER, C. A., TAKEUCHI, M., KAZAROSYAN, M., WANG, C. C., GUTTLER, R. B., SINGER, P. A., FATEMI, S., LOPRESTI, J. S. & NICOLOFF, J. T. 1998. Serum thyroglobulin autoantibodies: prevalence, influence on serum thyroglobulin measurement, and prognostic significance in patients with differentiated thyroid carcinoma. *J Clin Endocrinol Metab,* 83**,** 1121-7.

SPINELLI, C., BERTOCCHINI, A., ANTONELLI, A. & MICCOLI, P. 2004. Surgical therapy of the thyroid papillary carcinoma in children: Experience with 56 patients ≤16 years old. *Journal of Pediatric Surgery,* 39**,** 1500-1505.

SPINELLI, C., RALLO, L., MORGANTI, R., MAZZOTTI, V., INSERRA, A., CECCHETTO, G., MASSIMINO, M., COLLINI, P. & STRAMBI, S. 2019. Surgical management of follicular thyroid carcinoma in children and adolescents: A study of 30 cases. *J Pediatr Surg,* 54**,** 521-526.

SPINELLI, C., ROSSI, L., PISCIONERI, J., STRAMBI, S., ANTONELLI, A., FERRARI, A., MASSIMINO, M. & MICCOLI, P. 2016. Pediatric Differentiated Thyroid Cancer: When to Perform Conservative and Radical Surgery. *Curr Pediatr Rev,* 12**,** 247-252.

SPINELLI, C., TOGNETTI, F., STRAMBI, S., MORGANTI, R., MASSIMINO, M. & COLLINI, P. 2018. Cervical Lymph Node Metastases of Papillary Thyroid Carcinoma, in the Central and Lateral Compartments, in Children and Adolescents: Predictive Factors. *World J Surg,* 42**,** 2444-2453.

STELIAROVA-FOUCHER, E., STILLER, C. A., PUKKALA, E., LACOUR, B., PLESKO, I. & PARKIN, D. M. 2006. Thyroid cancer incidence and survival among European children and adolescents (1978-1997): report from the Automated Childhood Cancer Information System project. *Eur J Cancer,* 42**,** 2150-69.

STEVENS, C., LEE, J. K., SADATSAFAVI, M. & BLAIR, G. K. 2009. Pediatric thyroid fine-needle aspiration cytology: a meta-analysis. *J Pediatr Surg,* 44**,** 2184-91.

STEWART, D. R., BEST, A. F., WILLIAMS, G. M., HARNEY, L. A., CARR, A. G., HARRIS, A. K., KRATZ, C. P., DEHNER, L. P., MESSINGER, Y. H., ROSENBERG, P. S., HILL, D. A. & SCHULTZ, K. A. P. 2019. Neoplasm Risk Among Individuals With a Pathogenic Germline Variant in DICER1. *J Clin Oncol,* 37**,** 668-676.

STRATAKIS, C. A., COURCOUTSAKIS, N. A., ABATI, A., FILIE, A., DOPPMAN, J. L., CARNEY, J. A. & SHAWKER, T. 1997. Thyroid gland abnormalities in patients with the syndrome of spotty skin pigmentation, myxomas, endocrine overactivity, and schwannomas (Carney complex). *J Clin Endocrinol Metab,* 82**,** 2037-43.

SUCHY, B., WALDMANN, V. & KLUGBAUER, S. 1998. Absence of RAS and p53 mutations in thyroid carcinomas of children after Chernobyl. *British journal of cancer,* 77**,** 952-955.

SUGINO, K., ITO, K., NAGAHAMA, M., KITAGAWA, W., SHIBUYA, H., OHKUWA, K., YANO, Y., URUNO, T., AKAISHI, J., SUZUKI, A., MASAKI, C. & ITO, K. 2013. Diagnostic accuracy of fine needle aspiration biopsy cytology and ultrasonography in patients with thyroid nodules diagnosed as benign or indeterminate before thyroidectomy. *Endocr J,* 60**,** 375-82.

SUGINO, K., NAGAHAMA, M., KITAGAWA, W., SHIBUYA, H., OHKUWA, K., URUNO, T., SUZUKI, A., AKAISHI, J., MASAKI, C., MATSUZU, K.-I. & ITO, K. 2015. Papillary Thyroid Carcinoma in Children and Adolescents: Long-Term Follow-Up and Clinical Characteristics. *World Journal of Surgery,* 39**,** 2259-2265.

SUZUKI, S., NAKAMURA, I., SUZUKI, S., OHKOUCHI, C., MIZUNUMA, H., MIDORIKAWA, S., FUKUSHIMA, T., ITO, Y., SHIMURA, H., OHIRA, T., MATSUZUKA, T., OHTSURU, A., ABE, M., YAMASHITA, S. & SUZUKI, S. 2016. Inappropriate Suppression of Thyrotropin Concentrations in Young Patients with Thyroid Nodules Including Thyroid Cancer: The Fukushima Health Management Survey. *Thyroid,* 26**,** 717-725.

TAYLOR, A. J., CROFT, A. P., PALACE, A. M., WINTER, D. L., REULEN, R. C., STILLER, C. A., STEVENS, M. C. & HAWKINS, M. M. 2009. Risk of thyroid cancer in survivors of childhood cancer: results from the British Childhood Cancer Survivor Study. *Int J Cancer,* 125**,** 2400-5.

TRAHAN, J., REDDY, A., CHANG, E., GOMEZ, R., PRASAD, P. & JEYAKUMAR, A. 2016. Pediatric thyroid nodules: A single center experience. *Int J Pediatr Otorhinolaryngol,* 87**,** 94-7.

TUGGLE, C. T., ROMAN, S. A., WANG, T. S., BOUDOURAKIS, L., THOMAS, D. C., UDELSMAN, R. & ANN SOSA, J. 2008. Pediatric endocrine surgery: who is operating on our children? *Surgery,* 144**,** 869-77; discussion 877.

VAISMAN, F., BULZICO, D. A., PESSOA, C. H. C. N., BORDALLO, M. A. N., MENDONÇA, U. B. T. D., DIAS, F. L., COELI, C. M., CORBO, R. & VAISMAN, M. 2011. Prognostic factors of a good response to initial therapy in children and adolescents with differentiated thyroid cancer. *Clinics,* 66**,** 281-286.

VALI, R., RACHMIEL, M., HAMILTON, J., EL ZEIN, M., WASSERMAN, J., COSTANTINI, D. L., CHARRON, M. & DANEMAN, A. 2015. The role of ultrasound in the follow-up of children with differentiated thyroid cancer. *Pediatr Radiol,* 45**,** 1039-45.

VAN SANTEN, H. M., ARONSON, D. C., VULSMA, T., TUMMERS, R. F., GEENEN, M. M., DE VIJLDER, J. J. & VAN DEN BOS, C. 2004. Frequent adverse events after treatment for childhood-onset differentiated thyroid carcinoma: a single institute experience. *Eur J Cancer,* 40**,** 1743-51.

VASSILOPOULOII-SELLIN, R., KLEIN, M. J., SMITH, T. H., CANGIR, A. & HAYNIE, T. P. 1993. Pulmonary Metastases in Children and Young Adults with Differentiated Thyroid Cancer. *Cancer,* 71**,** 1348-1352.

VASSILOPOULOU-SELLIN, R., GOEPFERT, H., RANEY, B. & SCHULTZ, P. N. 1998. Differentiated thyroid cancer in children and adolescents clinical outcome and mortality after long-term follow-up. *Head and neck,* 20**,** 549-555.

VEIGA, L. H., LUBIN, J. H., ANDERSON, H., DE VATHAIRE, F., TUCKER, M., BHATTI, P., SCHNEIDER, A., JOHANSSON, R., INSKIP, P., KLEINERMAN, R., SHORE, R., POTTERN, L., HOLMBERG, E., HAWKINS, M. M., ADAMS, M. J., SADETZKI, S., LUNDELL, M., SAKATA, R., DAMBER, L., NETA, G. & RON, E. 2012. A pooled analysis of thyroid cancer incidence following radiotherapy for childhood cancer. *Radiat Res,* 178**,** 365-76.

VERBURG, F. A., BIKO, J., DIESSL, S., DEMIDCHIK, Y., DROZD, V., RIVKEES, S. A., REINERS, C. & HANSCHEID, H. 2011. I-131 activities as high as safely administrable (AHASA) for the treatment of children and adolescents with advanced differentiated thyroid cancer. *J Clin Endocrinol Metab,* 96**,** E1268-71.

VERBURG, F. A., MÄDER, U., LUSTER, M., HÄNSCHEID, H. & REINERS, C. 2015. Determinants of successful ablation and complete remission after total thyroidectomy and 131I therapy of paediatric differentiated thyroid cancer. *European Journal of Nuclear Medicine and Molecular Imaging,* 42**,** 1390-1398.

VERGAMINI, L. B., FRAZIER, A. L., ABRANTES, F. L., RIBEIRO, K. B. & RODRIGUEZ-GALINDO, C. 2014. Increase in the incidence of differentiated thyroid carcinoma in children, adolescents, and young adults: a population-based study. *J Pediatr,* 164**,** 1481-5.

VERLOOP, H., LOUWERENS, M., SCHOONES, J. W., KIEVIT, J., SMIT, J. W. & DEKKERS, O. M. 2012. Risk of hypothyroidism following hemithyroidectomy: systematic review and meta-analysis of prognostic studies. *J Clin Endocrinol Metab,* 97**,** 2243-55.

VRIENS, M. R., MOSES, W., WENG, J., PENG, M., GRIFFIN, A., BLEYER, A., POLLOCK, B. H., INDELICATO, D. J., HWANG, J. & KEBEBEW, E. 2011. Clinical and molecular features of papillary thyroid cancer in adolescents and young adults. *Cancer,* 117**,** 259-67.

VRIENS, M. R., SUH, I., MOSES, W. & KEBEBEW, E. 2009. Clinical features and genetic predisposition to hereditary nonmedullary thyroid cancer. *Thyroid,* 19**,** 1343-9.

WADA, N., SUGINO, K., MIMURA, T., NAGAHAMA, M., KITAGAWA, W., SHIBUYA, H., OHKUWA, K., NAKAYAMA, H., HIRAKAWA, S., RINO, Y., MASUDA, M. & ITO, K. 2009. Pediatric differentiated thyroid carcinoma in stage I: risk factor analysis for disease free survival. *BMC Cancer,* 9**,** 306.

WALLACE, W. H. 2011. Oncofertility and preservation of reproductive capacity in children and young adults. *Cancer,* 117**,** 2301-10.

WELCH-DINAUER, C. A., TUTTLE, R. M., ROBIE, D. K., MCCLELLAN, D. R., RITA, L., ADAIR, C., FRANCIS, G. L. & HE, E. 1998. Clinical features associated with metastasis and recurrence of differentiated thyroid cancer in children , adolescents and young adults. *Clinical Endocrinology,* 49**,** 619-628.

WELCH, A., MCCLELLAN, R., FRANCIS, G. L., TUTTLE, R. M. & ROBIE, D. K. 1999. Extensive surgery improves recurrence-free survival for children and young patients with class I papillary thyroid carcinoma. *Journal of Pediatric Surgery,* 34**,** 1799-1804.

WOOD, J. H., PARTRICK, D. A., BARHAM, H. P., BENSARD, D. D., TRAVERS, S. H., BRUNY, J. L. & MCINTYRE, R. C., JR. 2011. Pediatric thyroidectomy: a collaborative surgical approach. *J Pediatr Surg,* 46**,** 823-8.

XU, L., LIU, Q., LIU, Y. & PANG, H. 2016. Parameters Influencing Curative Effect of 131I Therapy on Pediatric Differentiated Thyroid Carcinoma: A Retrospective Study. *Medical science monitor : international medical journal of experimental and clinical research,* 22**,** 3079-85.

YAMASHITA, S. & SAENKO, V. 2007. Mechanisms of Disease: molecular genetics of childhood thyroid cancers. *Nat Clin Pract Endocrinol Metab,* 3**,** 422-9.

ZIMMERMAN, D., HAY, I. D., GOUGH, I. R., GOELLNER, J. R., RYAN, J. J., GRANT, C. S. & MCCONAHEY, W. M. 1988. Papillary thyroid carcinoma in children and adults: long-term follow-up of 1039 patients conservatively treated at one institution during three decades. *Surgery,* 104**,** 1157-66.

ZIVALJEVIC, V., TAUSANOVIC, K., SIPETIC, S., PAUNOVIC, I., DIKLIC, A., KOVACEVIC, B., STOJANOVIC, D., ZIVIC, R., STANOJEVIC, B. & KALEZIC, N. 2013. A case-control study of papillary thyroid cancer in children and adolescents. *Eur J Cancer Prev,* 22**,** 561-5.
